# Identification of Zebrafish Calcium Toolkit Genes and Their Expression in the Brain

**DOI:** 10.3390/genes10030230

**Published:** 2019-03-18

**Authors:** Iga Wasilewska, Rishikesh Kumar Gupta, Oksana Palchevska, Jacek Kuźnicki

**Affiliations:** 1 International Institute of Molecular and Cell Biology in Warsaw, Trojdena 4, 02-109 Warsaw, Poland; iwasilewska@iimcb.gov.pl (I.W.); rkgupta@iimcb.gov.pl (R.K.G.); opalchevska@iimcb.gov.pl (O.P.); 2 Postgraduate School of Molecular Medicine, Warsaw Medical University, 61 Żwirki i Wigury St., 02-091 Warsaw, Poland

**Keywords:** zebrafish, calcium toolkit, calcium homeostasis, calcium signaling, neurons, brain, bioinformatics, RNA-sequencing, gene ontology, RT-PCR

## Abstract

Zebrafish are well-suited for in vivo calcium imaging because of the transparency of their larvae and the ability to express calcium probes in various cell subtypes. This model organism has been used extensively to study brain development, neuronal function, and network activity. However, only a few studies have investigated calcium homeostasis and signaling in zebrafish neurons, and little is known about the proteins that are involved in these processes. Using bioinformatics analysis and available databases, the present study identified 491 genes of the zebrafish Calcium Toolkit (CaTK). Using RNA-sequencing, we then evaluated the expression of these genes in the adult zebrafish brain and found 380 hits that belonged to the CaTK. Based on quantitative real-time polymerase chain reaction arrays, we estimated the relative mRNA levels in the brain of CaTK genes at two developmental stages. In both 5 dpf larvae and adult zebrafish, the highest relative expression was observed for *tmbim4*, which encodes a Golgi membrane protein. The present data on CaTK genes will contribute to future applications of zebrafish as a model for in vivo and in vitro studies of Ca^2+^ signaling.

## 1. Introduction

Calcium ions (Ca^2+^) are essential for the regulation of intracellular processes and communication between cells. As a ubiquitous secondary messenger, they are involved in multiple signaling cascades [1]. To control and respond to such Ca^2+^ signals, cells require sophisticated machinery, namely the Calcium Toolkit (CaTK) that consists of Ca^2+^-binding and Ca^2+^-transporting proteins, including channels, pumps, and exchangers in plasma and organellar membranes [1,2]. Ca^2+^ concentrations inside cells are kept at a very low level of approx. 100 nM [3]. Ca^2+^ signals occur through an influx from the external environment or from intracellular storages, such as the endoplasmic reticulum (ER). The generation of such signals depletes Ca^2+^ storages. To maintain cellular Ca^2+^ homeostasis, these storages need to be refilled. The process that allows the maintenance of high Ca^2+^ concentrations inside the ER while Ca^2+^ flux and leakage occur is called store-operated calcium entry (SOCE) [4]. The proteins that are involved in this process include stromal interaction molecules (STIMs; i.e., sensors of Ca^2+^ levels inside the ER) and ORAI or transient receptor potential (TRP) channels [5,6,7,8,9] that enable Ca^2+^ influx into the cell. When ER Ca^2+^ levels decrease, STIMs undergo conformational changes, oligomerize, and bind to ORAI/TRP to activate them. These complexes can be detected as puncta-like structures at the plasma membrane (PM) [10]. Ca^2+^ that enters the cytosol is then pumped into the ER via sarcoplasmic/endoplasmic reticulum Ca^2+^ adenosine triphosphatases (SERCAs) [11].

Ca^2+^ homeostasis and signaling are especially important in neurons because they are involved in crucial processes, such as neurogenesis, neurotransmission, and synaptic plasticity [9,12]. Compared with non-excitable cells, neuronal CaTK is much more complex. This complexity is needed for the spatial and temporal regulation of Ca^2+^ that underlies proper neuronal function. These Ca^2+^-dependent processes are disrupted during aging and under many pathological conditions, including neurodegenerative disorders [13,14,15,16,17]. Neurons possess efficient Ca^2+^ influx pathways, including multiple voltage-gated calcium channels (VGCCs) and receptor-operated channels (ROCs) [18,19]. A neuronal SOCE (nSOCE)-like process has been identified [12,20]. Transcripts for SOC channels, including ORAI1, ORAI2, and ORAI3, and members of TRP subfamily C (TRPCs) were shown to be present in the mammalian brain [21,22]. Both STIM1 and STIM2 were also detected in the mammalian brain. STIM2 is expressed in human brain at levels that are comparable to STIM1. STIM2 is the predominant isoform in the murine central nervous system, especially in the hippocampus [23,24]. Ca^2+^ that enters neuronal cells through nSOCE appears to orchestrate various processes, such as gene expression, spine morphology, neuronal excitation, axonal growth, and the proliferation of progenitor cells (for review, see References [25,26,27,28]). However, analyses of nSOCE are challenging because of the existence of a plethora of players that maintain neuronal calcium homeostasis [29].

Zebrafish (*Danio rerio*) has been used extensively to study brain development, activity, and function and has emerged as a prominent model of disease states [30,31]. Zebrafish are a powerful model for investigating Ca^2+^-related processes in vivo because of their transparent larvae and ability to express Ca^2+^ probes that can be targeted to various cellular organelles [32]. Many recent studies have used Ca^2+^-sensing probes that are expressed in zebrafish brain [33,34,35,36]. However, the molecular mechanisms of Ca^2+^ signaling were not addressed directly, and the proteins that are involved were not identified. Only a few components of Ca^2+^-signaling machinery in zebrafish brain have been described to date [37,38], and a thorough analysis of CaTK genes in zebrafish has not been performed. In the present study, we collected information about members of the zebrafish CaTK and determined the levels of expression of genes in larval heads and the adult brain.

## 2. Materials and Methods

### 2.1. Animal Maintenance

Wildtype (AB line) zebrafish were used in the study. All of the animals were maintained according to previously described methods [39] in the Zebrafish Core Facility (ZCF) that is a licensed breeding and research facility (PL14656251—registry of the District Veterinary Inspectorate in Warsaw; 064 and 051—registry of the Ministry of Science and Higher Education) at the International Institute of Molecular and Cell Biology in Warsaw. All experiments with larvae and adult fish were performed in accordance to the European Communities Council Directive (63/2010/EEC). Adult zebrafish and larvae were kept in E3 medium (2.48 mM NaCl, 0.09 mM KCl, 0.164 mM CaCl_2_·2H_2_O, and 0.428 mM MgCl_2_·6H_2_O) at 28.5 °C.

### 2.2. Zebrafish Calcium Toolkit

To identify genes that belong to the zebrafish CaTK, we searched the Gene database of the National Center for Biotechnology Information (NCBI) [40] using various keywords for Ca^2+^ channels and Ca^2+^-binding proteins based on the literature (see Supplementary Materials). The members that were identified were further validated for their presence in zebrafish based on the Zebrafish Information Network (ZFIN) database, which is highly curated and maintained by the University of Oregon (Portland, OR, USA), is available online, and is extensively used by researchers worldwide [41].

Gene Ontology (GO) terms (biological process and molecular function) in Table S3 in the Supplementary Materials were taken from the Gene database of the NCBI. The data on zebrafish mutant phenotypes or expression were collected from the ZFIN database. The GO analysis was performed using the Protein ANalysis THrough Evolutionary Relationships (PANTHER) database (version 14.0) classification system. The genes were uploaded into PANTHER [42] to identify PANTHER-classified genes that are related to the GO terms. The *Danio rerio* genome was used as a reference gene list, which allowed the identification of cellular components, molecular functions, and related pathways from the GO terms.

### 2.3. RNA-Sequencing

Adult zebrafish were anesthetized with MS-222 (tricaine methanesulfonate), and the brains were dissected. The total RNA was extracted using TRI Reagent (Invitrogen, catalog no. AM9738) according to a published protocol [43], digested with DNase I, and purified with the RNA Clean and Concentrator Kit (ZYMO Research, catalog no. R1013) according to the manufacturer’s instructions. The sequencing procedure was performed using Illumina methodology. The preparation of the cDNA libraries and sequencing by Next-Generation Sequencing (NGS NextSeq 500) (run type: paired-end sequencing, read length: 1 × 76 bp) were performed in cooperation with the Core Facility at the International Institute of Molecular and Cell Biology. This resulted in approx. 120–150 million reads per sample with a 76 bp length. The reads were extracted in FASTQ format and used for the subsequent analysis. The reads were then aligned to the zebrafish Refseq genome assembly (GRCz11_genomic.fa) annotated genes using the Ensembl annotation file GRCz11_genomic.gff.

### 2.4. Real-Time Polymerase Chain Reaction Arrays of CaTK

Adult, 1-year-old zebrafish were anesthetized with MS-222, and the brains were dissected. The material from six fish was mixed for one RNA sample and homogenized in Qiazol (Qiagen, catalog no. 79306). The RNA from zebrafish larvae was prepared using the same protocol with the exception that 50 heads were dissected from the larvae with needles at 5 days postfertilization (dpf) and pooled as one sample. The RNA quality was verified by measuring the absorbance at 260, 280, and 230 nm. Only samples with A260/280 nm and A230/280 nm > 1.8 were used for the analysis. The RNA templates (1000 ng) were used to synthesize first-strand cDNA using iScript reverse transcription supermix (Bio-Rad, catalog no. 1708840). Real-time polymerase chain reaction (RT-PCR) was performed in duplicate using SsoAdvanced Universal SYBR Green Supermix (Bio-Rad, catalog no. 1725274), and 96-well custom plates that contained primers for target and reference genes (Bio-Rad). cDNA (25 ng) was used for each reaction. *Actin β 1* (*actb1*), *glyceraldehyde-3-phosphate dehydrogenase* (*gapdh*), *tyrosine 3-monooxygenase/tryptophan 5-monooxygenase activation protein zeta polypeptide* (*yhwaz*), and *ribosomal protein S18* (*rps18*) were used as reference genes. The data were analyzed using Bio-Rad CFX Maestro 1.0 software. The stability of the reference genes was checked using the Reference Gene Selection Tool, and all of them were evaluated as at least acceptable. The expression levels were calculated using the ΔCq method with an assumption of equal efficiencies for all reactions, and the average normalized expression was calculated for each gene according to the following formula:

$${\mathrm{Normalized}\;\mathrm{expression}}_{\mathrm{GOI}}=\frac{RQ_{GOI}}{{\left(RQ_{actb1}\times RQ_{gapdh}\times RQ_{yhwaz}\times RQ_{rps18}\right)}^{\frac{1}{4}}}$$

where GOI is the gene of interest and *RQ* is the relative quantity, calculated as $RQ=2^{\left(-Cq_{GOI}\right)}$.

The expression levels in two developmental stages were compared using an analysis of variance (ANOVA) and Tukey’s Honestly Significant Difference (HSD) post hoc test. Changes in the expression are presented as a fold change ± SD using an expression in larvae as a reference value. The charts were prepared using GraphPad Prism 5.

### 2.5. Real-Time PCR of Components of SOCE Expression Analysis

Adult, 1-year-old zebrafish were anesthetized with MS-222 and dissected. Various organs (i.e., brain, eyes, skeletal muscles, gonads, liver, and intestines) were removed and washed with phosphate-buffered saline (PBS). The material from two fish was pooled together, and the RNA was isolated using Qiazol (Qiagen, catalog no. 79306) according to a published protocol [43]. Samples from the larval heads and trunks were obtained by separating these parts with needles and pooling tissue from fifty 5 dpf zebrafish together. The RNA templates (1000 ng for larvae and 500 ng for organs from adult fish) were used to synthesize first-strand cDNA using the SuperScript IV First-Strand Synthesis System (Invitrogen, catalog no. 18091050). RT-PCR was performed in duplicate using FastStart Essential DNA Green Master (Roche, catalog no. 06402712001) and 50 ng cDNA for larvae and 25 ng cDNA for adults. *Eukaryotic translation elongation factor 1 α 1, like 1 (eef1a1l1*) was used as a reference gene. The primer sequences are listed in Table S1 in the Supplementary Materials. The data were analyzed using LightCycler 96 SW 1.1 (Roche) and Microsoft Excel. The efficiency of the PCR reaction was estimated based on an analysis of the dilution curves and slope calculations (Table S1). The expression levels were calculated using the 2^(−ΔCq)^ method. The charts were prepared using GraphPad Prism5.

## 3. Results

### 3.1. Members of the Zebrafish Brain CaTK

**Figure 1:**
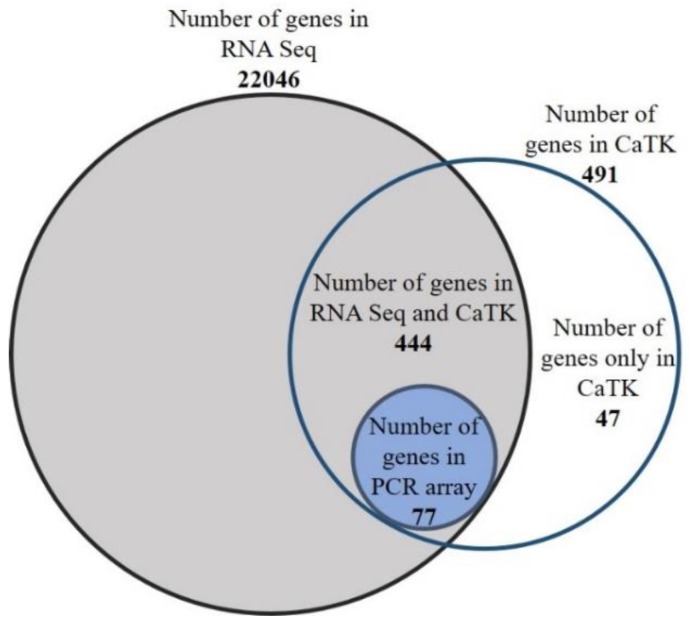
The scheme of the zebrafish Calcium Toolkit (CaTK): The expression of genes that were identified as members of the CaTK was examined by RNA-seq in the brain in adult zebrafish. Next, the mRNA levels of the selected genes were determined in zebrafish brain samples using RT-PCR arrays.

We identified 491 genes in zebrafish that were annotated in the Gene database of NCBI, which were members of the CaTK (Table S2 in Supplementary Materials). Approximately 4% of these genes were specific to zebrafish only. Next, we determined whether transcripts of these genes could be detected in the zebrafish brain. Using RNA-sequencing (RNA-seq) analysis, we detected transcripts for 22,046 genes in the samples that were obtained from adult zebrafish brains (Table S4). We found 444 CaTK hits of 491 predicted members among them (Figure 1, Table S3). For 77 genes which the expression patterns of are not yet established in zebrafish, we assessed the mRNA levels in larval heads and adult brains semiquantitatively using RT-PCR arrays (Figure 5).

**Figure 2:**
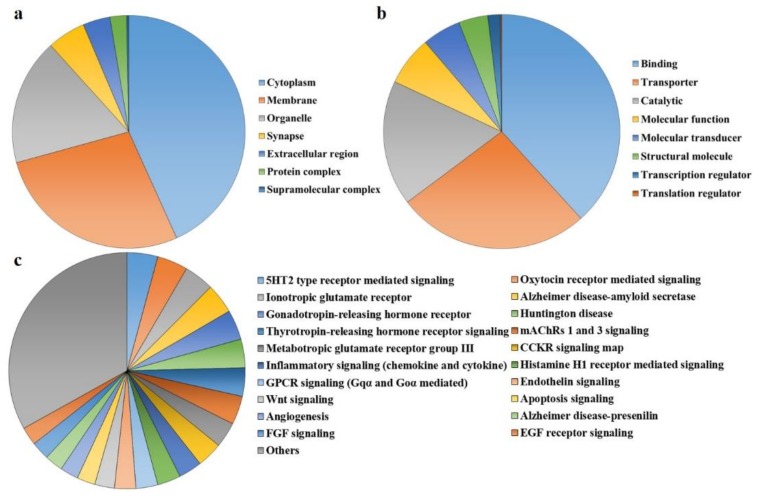
Protein ANalysis THrough Evolutionary Relationships (PANTHER) Gene Ontology (GO) annotation analysis [42]: The distribution of GO terms was categorized based on PANTHER GO-Slim. (**a**) The Cellular Component GO terms (components of cells or extracellular) with 393 component hits, (**b**) the Molecular Function GO terms (basic activities of a gene product at the molecular level, such as binding or catalysis) with 473 function hits, and (**c**) the Molecular Pathway GO terms (collection of molecular events or operation, with a strict definition of the beginning and end) with 592 pathway hits (429 genes in each annotation).

We next performed a GO annotation analysis of 444 brain CaTK genes using the PANTHER classification system and zebrafish genome as a reference gene list. A majority of the identified transcripts that are shown in Figure 2 encoded approx. 45% cytosolic proteins, approx. 25% membrane proteins, and nearly 20% organelle proteins. This classification roughly overlapped with the plausible annotated function: binding, transporter activity, and catalytic activity, respectively. The CaTK genes were classified into several signal transduction and metabolic pathways. Interestingly, approximately 10% of the genes were related to neurodegenerative diseases and belong to pathways that are associated with the pathogenesis of Alzheimer’s disease and Huntington’s disease. Among the most enriched pathways, we also found signaling via important neurotransmitters (serotonin, glutamate, acetylcholine, and histamine) and hormones that are released from hypothalamic neurons (oxytocin, thyrotropin-releasing hormone, and gonadotropin-releasing hormone).

As observed in the GO annotation analysis, the highest number of genes encodes proteins with binding abilities. We found the mRNA of genes that encode well-described Ca^2+^-binding proteins, such as calbindin, calretinin, parvalbumin, calmodulin, calumenin, and S100 Ca^2+^-binding proteins. Most of these proteins are localized in the cytosol, but some are present in the PM (e.g., neuronal Ca^2+^ sensors), ER (e.g., calreticulin and calnexin), and extracellular matrix (e.g., cell growth regulator with EF-hand domain and fibulin).

As expected, several active genes in the zebrafish brain encode Ca^2+^ channels that are localized in the cell membrane. They consist of a glutamate ionotropic *N*-methyl-D-aspartate (NMDA) receptor, (with especially high level of *grin1a*, *grin1b, grin2aa*, and *grin2bb*)*,* α-amino-3-hydroxy-5-methyl-4-isoxazolepropionic acid (AMPA) (with especially high levels of *gria2b, gria3a*, and *gria4a*) receptor subunits, subunits of VGCCs (*cacna*, *cacnb*, and *cacng* enriched with *cacna1aa*, *cacna1ab*, *cacna1c*, and *cacna1g*), TRP channels (*trpa*, *trpc*, *trpm,* and *trpv* enriched with *trpm7* and *trpc1*), and pore-forming subunits of CRAC channels (*orai1b* and *orai2*). Another large group encoded sodium/calcium exchangers (solute carrier family 8 (*scl8*) and family 24 (*scl24*) members). We also detected the transcripts for channels that are permeable to K^+^, the activity of which is Ca^2+^-dependent in the brain samples. They included K^+^ large conductance (*kcnm*s) and intermediate/small conductance (*kcnn*s) Ca^2+^-activated channels and potassium channel subfamily T (*kcnt*).

We also found the mRNA of genes that encode proteins that are responsible for Ca^2+^ release and transport across the ER membrane, such as inositol 1,4,5-trisphosphate (IP_3_) receptors (*itpr1a*, *itpr1b*, and *itpr2*), ryanodine receptors (*ryr1a*, *ryr1b*, *ryr2a*, and *ryr3*), and Serca. The highest mRNA level was observed for *ryr2a*, but *ryr1b* and *itpr1b* also reached high expression levels. We detected several mitochondrial Ca^2+^ transporters, including members of the solute carrier family 25 (*slc25a*), leucine zipper and EF-hand containing transmembrane proteins (*letm1* and *letm2*), components of the mitochondrial uniporter complex that consists of mitochondrial calcium uniporter (*mcu*) that forms a Ca^2+^ permeable pore, and three regulatory subunits (*micu1*, *micu2*, *micu3a*, and *micu3b*). *Micu1* and *letm2* were enriched in the brain tissue.

A similar percentage of genes that encode transport proteins in the CaTK comprises those with catalytic activity. Most of them are responsible for protein phosphorylation, such as calcium binding protein 39 (*cab39*), protein kinase C (*prkc*s, with the highest level of *prkcbb*), and serum/glucocorticoid regulated kinase (*sgk*s, with the highest level of *sgk1*).

A much smaller subgroup is involved in the regulation of transcription. This includes cyclic adenosine monophosphate response element binding proteins (*crebbpa* and *crebbpb*) and cyclic adenosine monophosphate response element binding protein-regulated transcription coactivators (*creb*s), cell cycle/division and apoptosis regulators (*ccar1* and *ccar2*), and amyloid β precursor binding family B members (*apbb*).

In addition to the mRNAs of *orai1a* and *orai2*, the expression of other genes that encode proteins that are involved in SOCE was detected in the zebrafish brain, including the Ca^2+^-binding Stim proteins and TRP Ca^2+^ channels (*trpc3*, *trpc6*) and SOCE-regulating proteins (calcium release activated channel regulator (*cracr*), SOCE-associated regulatory factor (*saraf*), and sigma non-opioid intracellular receptor (*sigmar1*)).

However, the RNA-seq analysis did not detect mRNAs of some genes that were identified in the zebrafish CaTK. There were no transcripts for several annexins, parvalbumins, Ca^2+^ voltage-gated channel, and TRP channel subunits. Voltage-dependent Ca^2+^ channels are multi-subunit complexes composed of α-1, β, α-2/delta, and γ subunits. Here, we detected most of the transcripts for genes that encode pore-forming α subunits. However, the transcript for *cacna1ea* was not detected despite known *Cacna1e* abundant expression in the rodent brain. α-2/delta, β, and γ are auxiliary subunits, that are important for the assembly and membrane localization of the complex, can modulate calcium currents and channel activation as well inactivation kinetics. Apart from *cacnb1* and *cacng3a*, all genes encoding those subunits were expressed in zebrafish brains. Among TRPC channels, neither transcripts for *trpc5b* nor *trpc7a* were found to be expressed in zebrafish brain and the transcript for *trpm1a* was also absent. Also, no transcripts for most of the calcium homeostasis modulators were detected by RNA-seq in the brain samples.

### 3.2. Expression of SOCE Components in Zebrafish

**Figure 3:**
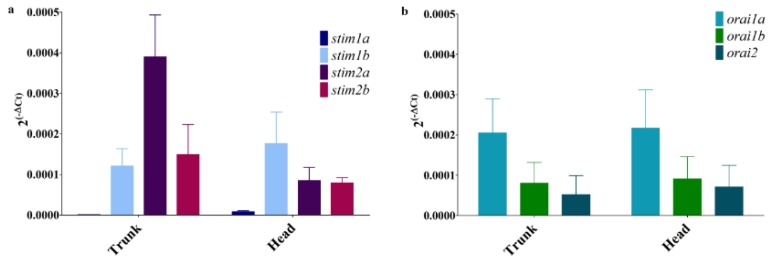
The mRNA levels of *stim*s (**a**) and *orais* (**b**) in zebrafish larval heads and trunks, estimated by RT-PCR: The data are presented as expression levels ± SD, calculated using the 2^(−ΔCq)^ method normalized to *eef1a1l1* (*n* = 3–4).

RNA-seq revealed low levels of mRNA encoding Stim proteins. To determine their expression pattern in the zebrafish, we applied another methodology and performed a precise analysis of a full set of zebrafish SOCE transcripts using RT-PCR. We first checked their mRNA levels in 5 dpf larvae. Surprisingly, we detected the expression of genes for all isoforms of *stim*s and *orai*s (Figure 3). The *stim1a* transcript was detectable at a very low level. The *stim1b* transcript was expressed predominately in the head, and the *stim2a* transcript was expressed predominately in the trunk. *Stim2b* expression was maintained at similar moderate levels in both the head and trunk of the larvae (Figure 3a). All three *orai* mRNA transcripts were present in the zebrafish head and trunk, with the highest relative expression of *orai1a* in both parts (Figure 3b).

**Figure 4:**
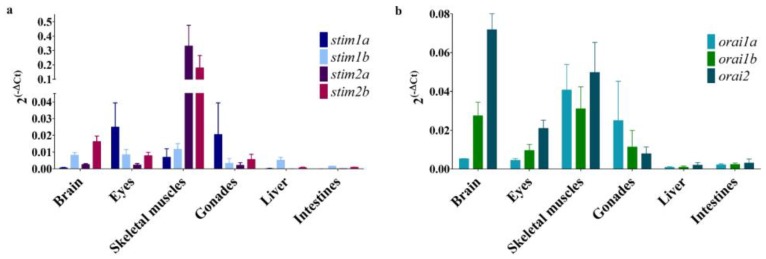
The mRNA levels of *stim*s (**a**) and *orai*s (**b**) in various tissues in 8-month-old zebrafish, estimated by RT-PCR: The data are presented as expression levels ± SD, calculated as 2^(−ΔCq)^ normalized to *eef1a1l1* (*n* = 3–4).

The mRNA levels of *stim*s and *orai*s were also estimated by RT-PCR in the brain in 8-month-old fish and in several other organs for comparison purposes (Figure 4). The level of mRNA that encode isoforms of these genes varied, depending on the tissue. In the brain, the highest relative expression was found for *stim2b* and *stim1b*. The relative expression of s*tim2a* was low, and the *stim1a* relative expression was very low. That is inconsistent with the RNA-seq results, which showed the highest level of *stim1a*, rather low levels of *stim2b* and *stim1b* and a very low amount of *stim2a* mRNA in the brain samples (Table S3). Differences between the RNA-Seq and RT-PCR results for some percentage of genes were previously reported [44], especially in the case of lowly expressed genes. Noteworthily, cDNA samples used in these two experiments were obtained using slightly different methods that also could affect the obtained results. Consistent with the relatively high *stim2a* level in the larval trunk, the high enrichment of *stim2a* was found in skeletal muscles. *Stim1a* was relatively highly expressed in the eyes and gonads, and the *stim1b* relative expression was high in the liver. In the liver and intestine, *stim* isoforms were expressed at very low levels (Figure 4a).

We detected a high relative level of *orai2* mRNA in the zebrafish brain, where it reached the highest relative level of expression and dominated over *orai1a* and *orai1b*. A similar expression pattern (i.e., with the highest *orai2* transcript level) was also detected in the eyes. Similar to *stim*s, a low *orai* isoforms expression was observed in the liver and intestine (Figure 4b).

### 3.3. Analysis of Expression Patterns of Previously Uncharacterized Members of the CaTK in Zebrafish Larval Head and Adult Brain

We next analyzed the relative expression of selected genes of the CaTK using RT-PCR. Of the 380 genes, we chose 77 (63 had unknown expression patterns and 14 had known expression patterns to validate the efficiency of the assay). Table S3 shows the CaTK reference genes which the expressions of were found in the ZFIN database [41].

**Figure 5:**
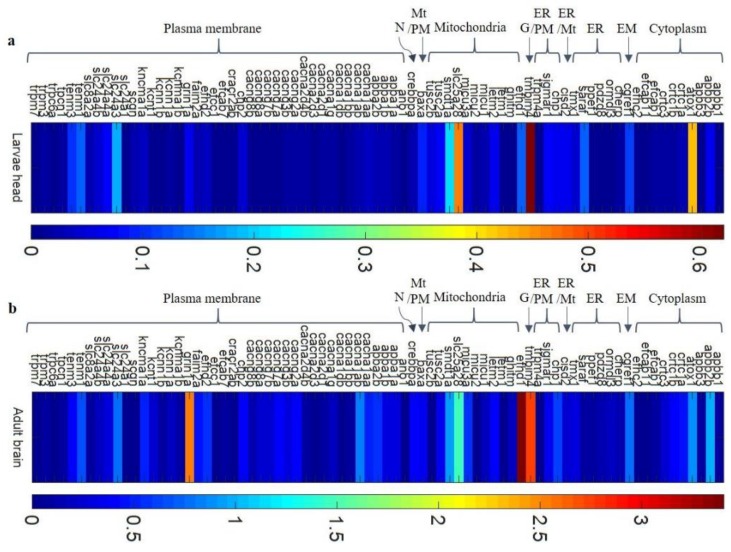
A heat map of the normalized expression levels of zebrafish brain CaTK genes, estimated by RT-PCR arrays: The expression levels of 77 CaTK genes in zebrafish brain tissue, including 14 genes for validation (*baxa*, *cacna1ab*, *cacng2a*, *chp1*, *crebbpa*, *crtc3*, *grin1a*, *kcnn1b*, *micu1*, *slc24a4a*, *tenm3*, and *trpm3*)*,* were normalized to the 4 reference genes. The expression levels were calculated using the ΔCq method with an assumption of equal efficiencies for all reactions. The relative mRNA levels are color-coded: dark blue (low) to red (high). Notice the different scales that are used for the results from larvae and adults. The RNA samples were obtained from larval heads (**a**) and from the brains of adult fish (**b**). *n* = 3. N, nucleus; EM, extracellular matrix; G, Golgi apparatus; Mt, mitochondria; PM, plasma membrane; and ER, endoplasmic reticulum.

We assessed the relative mRNA levels of 77 genes in the heads of 5 dpf larvae and in the brains of adult zebrafish (Figure 5). To compare the relative levels of different genes, we made an assumption that all of them were amplified with equal efficiency. At both stages, the highest relative expression was observed for *efhd1*, *slc25a28*, *apbb2b*, *cacna1ab*, *tmbim4*, *atox1*, and *cgref1*. This observation is roughly consistent with the RNA-seq results, that showed a high expression of a majority of these genes (*efhd1*, *apbb2b*, *cacna1ab*, and *tmbim4*). The expression of all of these genes is observed in the human brain [45]. In 5 dpf zebrafish larvae, the highest relative expression was observed for *tmbim4* (which encodes Golgi membrane protein) and *slc25a28* (which encodes the mitochondrial transmembrane transporter). In the adult zebrafish brain, the highest relative expression was observed for *efhd1* (which encodes mitochondrial Ca^2+^-binding protein) and *grin1a* (which encodes a subunit of the NMDA receptor NR1.1). The expression of the latter gene has been previously reported in the zebrafish brain [46]. Also, mRNA for *baxa*, *slc24a4a*, and *tenm3* were detected by us (on relatively low levels), and those genes were shown to be expressed in parts of zebrafish brain [47,48,49], thus indicating that our assay enables the detection of transcripts present in the brain.

**Figure 6:**
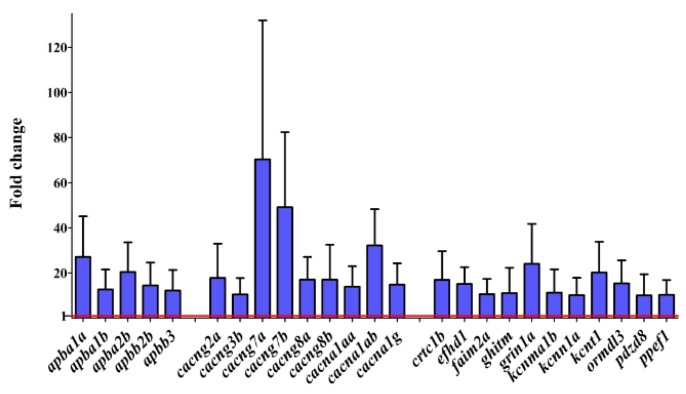
The changes in the expression of selected CaTK genes between mature fish and 5dpf larvae, quantified by RT-PCR arrays: Only genes with a fold change >10 and *p* < 0.05 are shown (Tukey’s Honestly Significant Difference test). The normalized expression in larvae was used as a reference value (= 1, marked as a red line). The data are presented as a fold change ± SD, normalized to the expression level in 5 dpf larvae (*n* = 3).

Approximately 30% of the genes had a stable level of expression. The expression of a majority of the genes (70%) significantly increased in the mature fish. The highest fold change (>10-fold change in expression at the larval stage) was observed for several genes that encode VGCCs (*cacna1aa*, *cacna1ab*, and *cacna1g*), an NMDA channel subunit (*grin1a*), and transmembrane regulators of Ca^2+^ channels (*cacng2a*, *cacng3b*, *cacng7a*, *cacng7b*, *cacng8a*, and *cacng8b*). The expression of a few K^+^ channels (*kcnma1b*, *kcnn1a,* and *kcnt1*) significantly increased during development (Figure 6). Moreover, we detected higher levels of transcripts for members of the amyloid β precursor protein-binding families A and B (*apba1a*, *apba1b*, *apba2b*, *apbb2b*, and *apbb3*). Increases in the mRNA levels of *ormdl3*, *pdzd8*, *efhd1*, *ghitm*, *faim2a*, *crtc1b,* and *ppef1* were also observed in the adult brain (Figure 6).

Only two genes, *cracr2ab* and *trpm4a*, presented an opposite effect, in which their mRNA levels dropped in adult fish (14- and three-fold, respectively). Cracr2ab is a Ca^2+^-binding protein that regulates Ca^2+^ transport by modulating SOCE. Trpm4a is a cation channel, the activity of which increases as intracellular Ca^2+^ concentrations increase. Both of these genes were shown to be expressed in the human brain throughout development, although at very low levels [45].

## 4. Discussion

Ca^2+^-mediated signaling regulates numerous cellular processes and involves a vast number of proteins that form a complex network of interactions. To make this possible, Ca^2+^ signals differ in speed, amplitude, and spatial patterning, with a broad repertoire of Ca^2+^-sensing, Ca^2+^-buffering, and Ca^2+^-transport proteins, comprising the CaTK [1]. This comprehensive machinery is especially broad in neuronal cells. Neurons possess numerous Ca^2+^ channels in different cellular compartments where they may have different functions, such as NMDA channels [50]. There is ample crosstalk between various signaling pathways (e.g., Ca^2+^ influx via SOCE refills the ER and may also regulate transcription [28]), cellular organelles (e.g., ER-mitochondria tethering [51]), and cellular systems (e.g., inflammation that affects central nervous system function [52]). To better understand these mechanisms and interactions of Ca^2+^-dependent processes in neurons, detailed analyses of the Ca^2+^-dependent players are needed.

The present study elucidated the zebrafish CaTK, further demonstrating that this animal model is useful for studying the molecular mechanisms of Ca^2+^ signaling and homeostasis. We found that 96% of zebrafish CaTK genes have mammalian orthologues that encode protein machinery for the aforementioned processes, thus allowing analyses of the role of Ca^2+^-dependent processes in fish that are relevant to mammals and the development of translational models of human diseases. We identified the expression patterns of numerous CaTK genes in the zebrafish brain and changes in their mRNA expression between two developmental stages (i.e., in 5 dpf larvae and the adult brain).

The genes we assigned to the zebrafish brain CaTK belong to various signaling pathways. Among the most enriched genes are those that signal important central nervous system neurotransmitters and neurohormones [53,54,55]. Approximately 7% of the genes are involved in various glutamatergic signaling pathways, including both ionotropic and metabotropic glutamate receptor (mGluR) pathways I, II, and III. Such an enrichment could be expected because glutamate is one of the most abundant neurotransmitters in the zebrafish central nervous system [53]. This indicates that many of the secretion and signal transduction processes depend on Ca^2+^ signaling [1,2], and zebrafish are a good animal model for studying them.

A large number of active CaTK genes in the brain in adult zebrafish encodes Ca^2+^ channels in the cell membrane. Genes that are involved in glutamatergic transmission were enriched, but we also identified genes that encode subunits of VGCCs and Trp and Orai channels. We also found genes that encode proteins that are responsible for Ca^2+^ transport in the ER (e.g., Serca and IP_3_ and ryanodine receptors), several mitochondrial Ca^2+^ transporters (e.g., Scl25 and Letm, components of the mitochondrial uniporter complex), and Ca^2+^-binding proteins (e.g., Calbindin1, Calbindin2 (Calretinin), Calreticulin, and Calnexin) [12,56,57,58]. The expression of all four isoforms of *stims* (*stim1a*, *stim1b*, *stim2a*, and *stim2b*) was detected in the brain of adult zebrafish. In mammals, different Stim1-to-Stim2 ratios are observed, depending on the specific tissue. In mice, Stim1 has a higher level of expression than Stim2, except in the brain where the Stim2 transcript levels dominate over Stim1 [24]. In humans, based on the RNA-seq data from the Expression Atlas [23], more uniform STIM1 and STIM2 expressions are observed, and the STIM1 expression predominates. In zebrafish, a high relative level of Stim1b and Stim2b expressions was observed in the brain, and both Stim2 isoforms predominated in muscles. This is in contrast to mammalian cells, in which the STIM1 splice variant STIM1L exists and is highly expressed in muscle tissue [59]. However, a similar sequence is not found in zebrafish, based on Ensembl (release 94) [60]. Based on Expression Atlas RNA-seq data, ORAI1 is the dominate ORAI channel in most human tissues. However, ORAI2 is highly enriched in the brain, the expression of which predominates over ORAI1 and ORAI3. A similar pattern was observed in mouse tissue [22] and in the zebrafish brain, as shown in the present work. The existence of nSOCE has been questioned [29], but further studies of nSOCE machinery in zebrafish might help resolve this issue.

RT-PCR arrays allowed us to validate the expression patterns for 63 CaTK genes, the expression patterns of which were not yet established in zebrafish. We compared their levels of expression in 5 dpf larval heads with the adult brain. Generally, approximately 70% of the genes had a higher expression in the adult brain than in the larvae. This could be related to the method of preparation, in which samples that were obtained from larval heads were only enriched in neuronal tissue and were contaminated with muscles and skin. However, this change may be the result of brain maturation, based on genes which the expression of increases in the adult brain.

The highest increase in expression was observed for genes that encode channels in the cell membrane and their regulators. These genes represented over half of the most differentially expressed genes. That indicates their importance in brain maturation and adult brain function. Among these, several *cacng* genes that encode transmembrane AMPA receptor regulatory proteins (TARPs) were found. TARPs modulate the gating properties of AMPA receptors and affect their trafficking and subcellular localization. Six classic TRAPs that are encoded by *cacng* genes are found in the mammalian central nervous system: γ2, γ3, γ4, γ5, γ7, and γ8 [61]. In the zebrafish brain, we detected transcripts for *cacng1a*, *cacng1b*, *cacng2a*, *cacng3b*, *cacng4a*, *cacng4b*, *cacng5a*, *cacng5b*, *cacng5a*, *cacng5b*, *cacng7a*, *cacng7b*, *cacng8a,* and *cacng8b*. In larval heads, the mRNA levels of most of them (except *cacng4b* and *cacng5b)* were at a very low level and significantly increased in the adult brain. A similar tendency was found in the human brain for *CACNG3*, whereas *CACGN2*, *CACGN7*, and *CACGN8* presented rather stable levels during maturation [45]. Increases in the mRNA levels of genes that encode pore-forming subunits of VGCCs (*cacna1aa*, *cacna1ab*, *cacna1ba*, *cacna1bb*, *cacna1c*, *cacna1da*, *cacna1db*, *cacna1eb*, *cacna1fa*, *cacna1fb, cacna1g*, *cacna1ha*, *cacna1hb*, and *cacna1i*) were also noted. *Cacna1g* encodes T-type, low-voltage activated calcium channels, the expression of which also increases during mouse development [62], reaches the highest level during childhood in the human brain [45], and is activated by the Lef1/β-catenin complex in thalamic neurons [63]. We also found an increase in the expression of the NMDA receptor subunit encoding gene *grin1a* and APP-binding family A and B genes. Most of these genes that encode the AMPA, NMDA, and CACN channels are associated with neurological diseases, such as epilepsy [64] and Alzheimer’s disease [65], thus making them a potential treatment target. Increases in the expression of members of the amyloid β precursor protein-binding families A and B were also found. These are neuronal adapter proteins that are involved in amyloid β production and play a role in the pathogenesis of Alzheimer’s disease [66]. Although zebrafish have been extensively studied as a model organism for neurodegenerative diseases and brain aging [31], the link between these studies and Ca^2+^ needs to be strengthened. The aforementioned genes are potential targets for further research on neurodegeneration with regards to the regulation of Ca^2+^.

The GO term analysis in the present study showed increases in the mRNA levels of proteins that are involved in important metabolic pathways in the mature zebrafish brain (e.g., the transcript for *ormdl3* that encodes a protein that is involved in sphingolipid synthesis [67] and *pdzd8*). Pdzd8 participates in lipid transport, the formation of ER-mitochondrion contacts, and the regulation of Ca^2+^ dynamics in mammalian neurons [68]. Additionally, the expression of *ghitm1* (which encodes a mitochondrial protein) and *faim2a* (which encodes a plasma membrane protein) increased in the mature brain, similar to observations in human brain samples [45]. However, little is known about the roles of these two proteins in the central nervous system. The expression of the protein serine/threonine phosphatase *ppef1* and transcriptional coactivator *crtc1b*, which present Ca^2+^-dependent activity, also increased in the adult zebrafish brain, but such increases were not observed for their orthologues in the human brain [45].

The transcripts of the following genes that encode proteins without well-defined functions were detected for the first time in the zebrafish brain: *atox1*, *calhm2*, *cgref1*, *cherp*, *tusc2*, and genes that encode proteins with EF-hand Ca^2+^-binding domains (e.g., *efcab1*, *efcab7*, *efcab11*, *efcc1*, *efhc2*, *efhd1*, *efhd2*, *necab1*, *necab2*, and *ppef1*). The *efhd1* gene seems to be of high interest. Although it already had a relatively high level of expression in 5 dpf larvae, its expression increased approx. 15-fold in the adult brain. A similar pattern was observed in the human brain [45]. EFHD1 has been shown to be involved in mitoflash activation in HeLa cells [69].

Very high relative mRNA levels of *tmbim4* and *slc25a28* were found in the zebrafish brain*,* with no major changes in expression during maturation. TMBIM4 is located in the Golgi apparatus and ER membrane and was shown to be ubiquitously expressed in mouse tissues [70]. TMBIM4 overexpression reduces Ca^2+^ release from internal stores [70,71], but its function in neuronal cells is unknown. SLC25a28 (mitoferrin-2) is a mitochondrial iron transporter. Disruptions of iron homeostasis may be involved in dopaminergic neuron degeneration in Parkinson’s disease [72].

Our research supports the use of zebrafish for studying Ca^2+^ homeostasis and signaling. The transparency of its larvae, the rapid development of the nervous system, and the ease of genetic manipulation make zebrafish useful for many types of research. Zebrafish embryos can be genetically manipulated relatively easily for transient or stable protein expression, knockouts, or other types of mutations [39]. Functional conservation among vertebrates (approx. 70% of human genes have at least one zebrafish orthologue) makes zebrafish an outstanding model for examining the molecular mechanisms of human diseases [73], assaying gene activity, and discovering novel compounds for the treatment of disease. CRISPR/Cas9 technology is well-established for zebrafish, and crispants can be created to introduce mutations of specific genes [30]. This creates the possibility of generating fish models of human diseases. Moreover, Ca^2+^ probes can be introduced to target the cytosol, ER, mitochondria, and lysosomes, thus permitting analyses of Ca^2+^ levels [32,74]. This provides many opportunities to implement various experimental approaches, including in vivo visualization and analyses of the dynamics of Ca^2+^ signaling.

## 5. Conclusions and Future Directions

The present CaTK data will be useful for designing experiments to dissect the functions of specific genes and to understand the molecular mechanisms of Ca^2+^ homeostasis and signaling in wildtype fish and models of neurological diseases. Ca^2+^ homeostasis and signaling are dysregulated in many neurological diseases [12,75]. The fact that zebrafish are amenable to in vivo experiments and the high conservation of CaTK members (96% as found in the present study) allow broad investigations of Ca^2+^ signaling at the systemic level. The genes that were identified in the present study will allow further studies of neurodevelopment, neuronal Ca^2+^ signaling, and neurodegeneration in zebrafish as a model organism.

## Supplementary Materials

The following are available online at https://www.mdpi.com/2073-4425/10/3/230/s1, Table S1: Primer sequences, Table S2: Zebrafish Calcium Toolkit, Table S3: Zebrafish brain Calcium Toolkit, and Table S4: RNA Seq data.
