# Supplementary material for: Identification of Zebrafish Calcium Toolkit Genes and Their Expression in the Brain
Source: Genes (Basel). 2019 Mar 18;10(3):230. doi: 10.3390/genes10030230 (PMC6471419; doi:10.3390/genes10030230)
Supplement: Supplementary file 1 [file genes-10-00230-s001.zip › Supplementary matrials-IW.pdf]

Supplementary Materials

Key-words used for identification of genes belonging to zebrafish CaTK:
calcium channels, receptors and proteins in ER, calcium ATPases in PM and ER, cytosolic calcium-binding proteins, calcium-dependent chaperones, calcium exchangers, transcription factors, mitochondria enzymes activated by calcium, calcium-sensing receptor, and calcium signaling regulatory proteins

Table S1

Primer name	Primer sequence	Slope	Efficiency [%]
eef1a1l1F	AAAATCGGTGGTGCTGGCAA	-3.2670	102
eef1a1l1R	GGAACGGTGTGATTGAGGGA		
stim1aF	TGAATTCGGATTGCCAGTCGT	-3.2005	105
stim1aR	TTCAAGTCCCTCTGCGAACC		
stim1bF	TGAGTTTTGAGGCCATCCGC	-3.0570	112
stim1bR	AACCCATCCGTCTCTGTCAC		
stim2aF	ATTACGGAGGCGGATCGATT	-3.0570	112
stim2aR	CCTCAATGCCTCCATCCTGA		
stim2bF	CTGGTGGAGTGGACGATCTT	-2.9835	116
stim2bR	CGTCAGAGGAGGTCGAATCA		
orai1aF	GTGCATTTTTACCGCTCGCT	-3.0041	115
orai1aR	TTGAAGAGGCATCTCCCCTC		
orai1bF	GCTGTAAGCAACGTGCACAA	-3.0190	114
orai1bR	TCCCGATGACGGTGGAAAAG		
orai2F	CGAGCTAGCCTGGGGTTTTT	-3.0500	113
orai2R	AGTCAACCGGCAGGAACTTG		

Table S2 Zebrafish Calcium Toolkit

gene	gene name	gene ID	chr	human orthologs	mouse orthologs
aif1l	allograft inflammatory factor 1-like	321033	5	*AIF1L*	*Aif1l*
akt1	v-akt murine thymoma viral oncogene homolog 1	101910198	17	*AKT1*	*Akt1*
akt2	v-akt murine thymoma viral oncogene homolog 2	378972	18	*AKT2*	*Akt2*
akt3b	v-akt murine thymoma viral oncogene homolog 3b	100149794	1	*AKT3*	*Akt3*
ano1	anoctamin 1, Ca^{2+} activated chloride channel	407698	7	*ANO1*	*Ano1*
anxa11a	annexin A11a	368215	13	*ANXA11*	*Anxa11*
anxa11b	annexin A11b	353365	12	*ANXA11*	*Anxa11*
anxa13	annexin A13	81880	24	*ANXA13*	*Anxa13-*
anxa13l	annexin A13, like	554118	2	*ANXA13*	*Anxa13-*
anxa1a	annexin A1a	334724	5	*ANXA1*	*Anxa1*
anxa1b	annexin A1b	353358	5	*ANXA1*	*Anxa1*
anxa1c	annexin A1c	494158	5	*ANXA1*	*Anxa1*
anxa2a	annexin A2a	325557	25	*ANXA2*	*Anxa2*
anxa2b	annexin A2b	799806	7	*ANXA2*	*Anxa2*
anxa3a	annexin A3a	447893	6	*ANXA3*	*Anxa3*
anxa3b	annexin A3b	492336	10	*ANXA3*	*Anxa3*
anxa4	annexin A4	353362	10	*ANXA4*	*Anxa4*

anxa5a	annexin A5a	557578	14	*ANXA5*	*Anxa5*
anxa5b	annexin A5b	337132	1	*ANXA5*	*Anxa5*
anxa6	annexin A6	353363	14	*ANXA6*	*Anxa6*
apba1a	amyloid beta (A4) precursor protein-binding, family A, member 1a	100003938	5	*APBA1*	*Apba1*
apba1b	amyloid beta (A4) precursor protein-binding, family A, member 1b	565507	10	*APBA1*	*Apba1*
apba2a	amyloid beta (A4) precursor protein-binding, family A, member 2a	100330365	25	*APBA2*	*Apba2*
apba2b	amyloid beta (A4) precursor protein-binding, family A, member 2b	327160	7	*APBA2*	*Apba2*
apbb1	amyloid beta (A4) precursor protein-binding, family B, member 1 (Fe65)	565164	10	*APBB1*	*Apbb1*
apbb1ip	amyloid beta (A4) precursor protein-binding, family B, member 1 interacting protein	393607	24	*APBB1IP*	*Apbb1ip*
apbb2b	amyloid beta (A4) precursor protein-binding, family B, member 2b	564990	1	*APBB2*	*Apbb2*
apbb3	amyloid beta (A4) precursor protein-binding, family B, member 3	101883158	21	*APBB3*	*Apbb3*
atox1	antioxidant 1 copper chaperone	558347	14	*ATOX1*	*Atox1*
atp2a1	ATPase sarcoplasmic/endoplasmic reticulum Ca^{2+} transporting 1	260440	3	*ATP2A1*	*Atp2a1*
atp2a1l	ATPase sarcoplasmic/endoplasmic reticulum Ca^{2+} transporting 1, like	494489	12	*ATP2A1*	*Atp2a1*
atp2a2a	ATPase sarcoplasmic/endoplasmic reticulum Ca^{2+} transporting 2a	393940	8	*ATP2A2*	*Atp2a2*
atp2a2b	ATPase sarcoplasmic/endoplasmic reticulum Ca^{2+} transporting 2b	568265	21	*ATP2A2*	*Atp2a2*
atp2a3	ATPase sarcoplasmic/endoplasmic reticulum Ca^{2+} transporting 3	568671	5	*ATP2A3*	*Atp2a3*
atp2b1a	ATPase plasma membrane Ca^{2+} transporting 1a	378746	4	*ATP2B1*	*Atp2b1*
atp2b1b	ATPase plasma membrane Ca^{2+} transporting 1b	561190	25	*ATP2B4*	*Atp2b4*
atp2b2	ATPase plasma membrane Ca^{2+} transporting 2	557430	11	*ATP2B*	*Atp2b2*
atp2b3a	ATPase, Ca^{2+} transporting, plasma membrane 3a	436745	8	*ATP2B3*	*Atp2b3*
atp2b3b	ATPase plasma membrane Ca^{2+} transporting 3b	558525	23	*ATP2B3*	*Atp2b3*
atp2b4	ATPase plasma membrane Ca^{2+} transporting 4	768304	11	*ATP2B4*	*Atp2b4*
atp2c1	ATPase secretory pathway Ca^{2+} transporting 1	559574	16	*ATP2C1*	*Atp2c1*
atp6v0cb	ATPase H^{+} transporting V0 subunit cb	325402	24	*ATP6V0C*	*Atp6v0c*
baxa	BCL2 associated X, apoptosis regulator a	58081	3	*BAX*	*Bax*
baxb	BCL2 associated X, apoptosis regulator b	503609	3	*BAX*	*Bax*
cab39	Ca^{2+} binding protein 39	415235	15	*CAB39*	*Cab39*
cab39l	Ca^{2+} binding protein 39-like	492361	1	*CAB39L*	*Cab39l*
cabp1a	Ca^{2+} binding protein 1a	449789	8	*CABP1*	*Cabp1*
cabp1b	Ca^{2+} binding protein 1b	436687	10	*CABP1*	*Cabp1*
cacna1aa	voltage-dependent, P/Q type, alpha 1A subunit, a	562059	3	*CACNA1A*	*Cacna1a*
cacna1ab	voltage-dependent, P/Q type, alpha 1A subunit, b	569528	11	*CACNA1A*	*Cacna1a*
cacna1ba	voltage-dependent, N type, alpha 1B subunit, a	555941	5	*CACNA1B*	*Cacna1b*
cacna1bb	voltage-dependent, N type, alpha 1B subunit, a	562796	21	*CACNA1B*	*Cacna1b*
cacna1c	voltage-dependent, L type, alpha 1C subunit	170581	4	*CACNA1C*	*Cacna1c*
cacna1da	voltage-dependent, L type, alpha 1D subunit, a	403310	11	*CACNA1D*	*Cacna1d*
cacna1db	voltage-dependent, L type, alpha 1D subunit, b	403311	8	*CACNA1D*	*Cacna1d*
cacna1ea	voltage-dependent, R type, alpha 1E subunit a	564274	8	*CACNA1E*	*Cacna1e*
cacna1eb	voltage-dependent, R type, alpha 1E subunit b	567702	2	*CACNA1E*	*Cacna1e*
cacna1fa	voltage-dependent, L type, alpha 1F subunit a	793708	8	*CACNA1F*	*Cacna1f*
cacna1fb	voltage-dependent, L type, alpha 1F subunit	559964	8	*CACNA1F*	*Cacna1f*
cacna1g	voltage-dependent, T type, alpha 1G subunit	563904	12	*CACNA1G*	*Cacna1g*
cacna1ha	voltage-dependent, T type, alpha 1H subunit a	560875	3	*CACNA1H*	*Cacna1h*
cacna1hb	voltage-dependent, T type, alpha 1H subunit b	100007317	1	*CACNA1H*	*Cacna1h*
cacna1i	voltage-dependent, T type, alpha 1I subunit	566351	3	*CACNA1I*	*Cacna1i*
cacna1sa	voltage-dependent, L type, alpha 1S subunit, a	570683	22	*CACNA1S*	*Cacna1s*
cacna1sb	voltage-dependent, L type, alpha 1S subunit, b	405791	8	*CACNA1S*	*Cacna1s*
cacna2d1a	voltage-dependent, alpha 2/delta subunit 1a	561434	4	*CACNA2D1*	*Cacna2d1*
cacna2d2a	voltage-dependent, alpha 2/delta subunit 2a	541376	6	*CACNA2D2*	*Cacna2d2*
cacna2d2b	voltage-dependent, alpha 2/delta subunit 2b	568759	22	*CACNA2D2*	*Cacna2d2*
cacna2d3	voltage dependent, alpha2/delta subunit 3	100148645	11	*CACNA2D3*	*Cacna2d3*

cacna2d3a	voltage-dependent, alpha 2/delta subunit 3a	100535196	8	*CACNA2D3*	*Cacna2d3*
cacna2d4a	voltage-dependent, alpha 2/delta subunit 4a	568225	4	*CACNA2D4*	*Cacna2d4*
cacna2d4b	voltage-dependent, alpha 2/delta subunit 4b	100150428	25	*CACNA2D4*	*Cacna2d4*
cacnb1	voltage-dependent, beta 1 subunit	436925	3	*CACNB1*	*Cacnb1*
cacnb2a	voltage-dependent, beta 2a	795492	7	*CACNB2*	*Cacnb2*
cacnb2b	voltage-dependent, beta 2b	558806	2	*CACNB2*	*Cacnb2*
cacnb3a	voltage-dependent, beta 3a	565158	23	*CACNB3*	*Cacnb3*
cacnb3b	voltage-dependent, beta 3b	559015	23	*CACNB3*	*Cacnb3*
cacnb4a	voltage-dependent, beta 4a subunit	562422	9	*CACNB4*	*Cacnb4*
cacnb4b	voltage-dependent, beta 4b subunit	678560	6	*CACNB4*	*Cacnb4*
cacng1a	voltage-dependent, gamma subunit 1a	322013	6	*CACNG1*	*Cacng1*
cacng1b	voltage-dependent, gamma subunit 1b	571193	3	*CACNG1*	*Cacng1*
cacng2a	voltage-dependent, gamma subunit 2a	393614	3	*CACNG2*	*Cacng2*
cacng3a	voltage-dependent, gamma subunit 3a	567438	1	*CACNG3*	*Cacng3*
cacng3b	voltage-dependent, gamma subunit 3b	564883	3	*CACNG3*	*Cacng3*
cacng4a	voltage-dependent, gamma subunit 4a	797554	6	*CACNG4*	*Cacng4*
cacng4b	voltage-dependent, gamma subunit 4b	794222	3	*CACNG4*	*Cacng4*
cacng5a	voltage-dependent, gamma subunit 5a	797409	6	*CACNG5*	*Cacng5*
cacng5b	voltage-dependent, gamma subunit 5b	100331116	3	*CACNG5*	*Cacng5*
cacng6a	voltage-dependent, gamma subunit 6a	100034580	19	*CACNG6*	*Cacng6*
cacng6b	voltage-dependent, gamma subunit 6b	100003220	16	*CACNG6*	*Cacng6*
cacng7a	voltage-dependent, gamma subunit 7a	100034540	19	*CACNG7*	*Cacng7*
cacng7b	voltage-dependent, gamma subunit 7b	100003278	16	*CACNG7*	*Cacng7*
cacng8a	voltage-dependent, gamma subunit 8a	550428	19	*CACNG8*	*Cacng8*
cacng8b	voltage-dependent, gamma subunit 8b	100003329	16	*CACNG8*	*Cacng8*
cadps2	Ca^{2}+-dependent secretion activator 2	407617	25	*CADPS2*	*Cadps2*
cadpsa	Ca^{2+}-dependent activator protein for secretion a	100333933	11	*CADPS*	*Cadps*
cadpsb	Ca^{2+}-dependent activator protein for secretion b	100001129	6	*CADPS*	*Cadps*
calb1	calbindin 1	100001650	16	*CALB1*	*Calb1*
calb2a	calbindin 2a	393691	18	*CALB2*	*Calb2*
calb2b	calbindin 2b	393684	7	*CALB2*	*Calb2*
calhm1	Ca^{2+} homeostasis modulator 1	100005176	13	*CALHM1*	*Calhm1*
calhm2	Ca^{2+} homeostasis modulator 2	368657	13	*CALHM2*	*Calhm2*
calhm3	Ca^{2+} homeostasis modulator 3	100005130	13	*CALHM3*	*Calhm3*
calhm5.1	Ca^{2+} homeostasis modulator family member 5, tandem duplicate 1	100037308	16	*CALHM5*	*Calhm5*
calhm5.2	Ca^{2+} homeostasis modulator family member 5, tandem duplicate 2	798204	16	*CALHM5*	*Calhm5*
calhm6	Ca^{2+} homeostasis modulator family member 6	780840	16	*FAM26F*	*Fam26f*
calm1a	calmodulin 1a	406660	17	*CALM1*	*Calm1*
calml4a	calmodulin-like 4a	560151	7	*CALML4*	*Calml4*
calml4b	calmodulin-like 4b	100321746	25	*CALML4*	*Calml4*
calr	calreticulin	325317	8	*CALR*	*Calr*
calr3a	calreticulin 3a	30248	22	*CALR3*	*Calr3*
calr3b	calreticulin 3b	321315	2	*CALR3*	*Calr3*
calua	calumenin a	415241	4	*CALU*	*Calu*
calub	calumenin b	394057	18	*CALU*	*Calu*
canx	calnexin	406757	14	*CANX*	*Canx*
capn10	calpain 10	570311	2	*CAPN10*	*Capn10*
capn12	calpain 12	799247	18	*CAPN12*	*Capn12*
capn15	calpain 15	100330198	24	*CAPN15*	*Capn15*
capn1a	calpain 1, (mu/I) large subunit a	393417	13	*CAPN1*	*Capn1*
capn1b	calpain 1, (mu/I) large subunit b	565106	22	*CAPN1*	*Capn1*
capn2a	calpain 2, (m/II) large subunit a	541374	13	*CAPN2*	*Capn2*

capn2b	calpain 2, (m/II) large subunit b	563053	22	*CAPN2*	*Capn2*
capn2l	calpain 2, (m/II) large subunit, like	550505	22	*CAPN2*	*Capn2*
capn3a	calpain 3a, (p94)	447832	17	*CAPN3*	*Capn3*
capn3b	calpain 3b	100034405	20	*CAPN3*	*Capn3*
capn5a	calpain 5a	563346	18	*CAPN5*	*Capn5*
capn5b	calpain 5b	100006332	21	*CAPN5*	*Capn5*
capn7	calpain 7	556792	22	*CAPN7*	*Capn7*
capn9	calpain 9	445107	20	*CAPN9*	*Capn9*
casr	Ca^{2+} sensing receptor	560607	5	*CASR*	*Casr*
cbarpb	voltage-dependent, beta subunit associated regulatory protein b	557421	22	*CBARP*	*Cbarp*
ccar1	cell division cycle and apoptosis regulator 1	568087	13	*CCAR1*	*Ccar1*
ccar2	cell cycle and apoptosis regulator 2	564155	10	*CCAR2*	*Ccar2*
ccbe1	collagen and Ca^{2+}binding EGF domains 1	555629	21	*CCBE1*	*Ccbe1*
cetn2	centrin, EF-hand protein, 2	100006257	14	*CETN2*	*Cetn2*
cetn3	centrin 3	552931	10	*CETN3*	*Cetn3*
cetn4	centrin 4	795310	14	*CETN4P*	*Cetn4*
cgref1	cell growth regulator with EF-hand domain 1	559576	17	*CGREF1*	*Cgref1*
cherp	Ca^{2+}homeostasis endoplasmic reticulum protein	337851	22	*CHERP*	*Cherp*
chp1	calcineurin-like EF-hand protein 1	325361	13	*CHP1*	*Chp1*
chp2	calcineurin-like EF-hand protein 2	327599	15	*CHP2*	*Chp2*
cisd2	CDGSH iron sulfur domain 2	393354	1	*CISD2*	*Cisd2*
clgn	calmegin	556495	1	*CLGN*	*Clgn*
clstn1	calsyntenin 1	777737	23	*CLSTN1*	*Clstn1*
clstn2	calsyntenin 2	100125749	2	*CLSTN2*	*Clstn2*
clstn3	calsyntenin 3	555627	16	*CLSTN3*	*Clstn3*
cracr2aa	Ca^{2+}release activated channel regulator 2Aa	100330395	18	*CRACR2A*	*Cracr2a*
cracr2ab	Ca^{2+}release activated channel regulator 2Ab	564347	4	*CRACR2A*	*Cracr2a*
cracr2b	Ca^{2+}release activated channel regulator 2B	790936	25	*CRACR2B*	*Cracr2b*
creb1a	cAMP responsive element binding protein 1a	573207	1	*CREB1*	*Creb1*
creb1b	cAMP responsive element binding protein 1b	550516	9	*CREB1*	*Creb1*
creb5a	cAMP responsive element binding protein 5a	101886947	19	*CREB5*	*Creb5*
creb5b	cAMP responsive element binding protein 5b	565910	16	*CREB5*	*Creb5*
crebl2	cAMP responsive element binding protein-like 2	436975	4	*CREBL2*	*Crebl2*
creb3l1	cAMP responsive element binding protein 3-like 1	338317	7	*CREB3L1*	*Creb3l1*
creb3l2	cAMP responsive element binding protein 3-like 2	791169	4	*CREB3L2*	*Creb3l2*
creb3l3a	cAMP responsive element binding protein 3-like 3a	553700	22	*CREB3L3*	*Creb3l3*
creb3l3b	cAMP responsive element binding protein 3-like 3b	100538072	2	*CREB3L3*	*Creb3l3*
creb3l4	cAMP responsive element binding protein 3-like 4	100333318	16	*CREB3L4*	*Creb3l4*
crebbpa	CREB binding protein a	566841	22	*CREBBP*	*Crebbp*
crebbpb	CREB binding protein b	567111	3	*CREBBP*	*Crebbp*
crebrf	creb3 regulatory factor	100149876	21	*CREBRF*	*Crebrf*
crebzf	CREB/ATF bZIP transcription factor	562093	10	*CREBZF*	*Crebzf*
creld2	cysteine-rich with EGF-like domains 2	393495	4	*CRELD2*	*Creld2*
crtc1a	CREB regulated transcription coactivator 1a	768293	2	*CRTC1*	*Crtc1*
crtc1b	CREB regulated transcription coactivator 1b	571344	11	*CRTC1*	*Crtc1*
crtc2	CREB regulated transcription coactivator 2	799532	19	*CRTC2*	*Crtc2*
crtc3	CREB regulated transcription coactivator 3	570024	7	*CRTC3*	*Crtc3*
efcab1	EF-hand Ca^{2+}binding domain 1	558421	19	*EFCAB1*	*Efcab1*
efcab11	EF-hand Ca^{2+}binding domain 11	550510	17	*EFCAB11*	*Efcab11*
efcab2	EF-hand Ca^{2+}binding domain 2	558031	17	*EFCAB2*	*Efcab2*
efcab6	EF-hand Ca^{2+}binding domain 6	492579	25	*EFCAB6*	*Efcab6*
efcab7	EF-hand Ca^{2+}binding domain 7	415210	6	*EFCAB7*	*Efcab7*

efcc1	EF-hand and coiled-coil domain containing 1	100333996	11	*EFCC1*	*Efcc1*
efemp1	EGF containing fibulin extracellular matrix protein 1	100332567	13	*EFEMP1*	*Efemp1*
efemp2a	EGF containing fibulin extracellular matrix protein 2a	494044	14	*EFEMP2*	*Efemp2*
efemp2b	EGF containing fibulin extracellular matrix protein 2b	572703	21	*EFEMP2*	*Efemp2*
efhb	EF-hand domain family, member B	571184	16	*EFHB*	*Efhb*
efhc1	EF-hand domain (C-terminal) containing 1	393942	20	*EFHC1*	*Efhc1*
efhc2	EF-hand domain (C-terminal) containing 2	567323	9	*EFHC2*	*Efhc2*
efhd1	EF-hand domain family, member D1	335521	15	*EFHD1*	*Efhd1*
efhd2	EF-hand domain family, member D2	571114	8	*EFHD2*	*Efhd2*
egfl7	EGF-like-domain, multiple 7	678640	21	*EGFL7*	*Egfl7*
ehd1a	EH-domain containing 1a	405810	14	*EHD1*	*Ehd1*
ehd1b	EH-domain containing 1b	447839	10	*EHD1*	*Ehd1*
ehd2a	EH-domain containing 2a	567055	5	*EHD2*	*Ehd2*
ehd2b	EH-domain containing 2b	337401	18	*EHD2*	*Ehd2*
ehd3	EH-domain containing 3	562949	20	*EHD3*	*Ehd3*
ehd4	EH-domain containing 4	569546	17	*EHD4*	*Ehd4*
eps15	epidermal growth factor receptor pathway substrate 15	795505	8	*EPS15*	*Eps15*
eps15l1a	epidermal growth factor receptor pathway substrate 15-like 1a	568170	22	*EPS15L1*	*Eps15l1*
eps15l1b	epidermal growth factor receptor pathway substrate 15-like 1b	100329948	2	*EPS15L1*	*Eps15l1*
faim2a	Fas apoptotic inhibitory molecule 2a	541391	2	*FAIM2*	*Faim2*
faim2b	Fas apoptotic inhibitory molecule 2b	100006044	11	*FAIM2*	*Faim2*
fbln1	fibulin 1	30240	25	*FBLN1*	*Fbln1*
fbln2	fibulin 2	553503	11	*FBLN2*	*Fbln2*
fbln5	fibulin 5	449806	13	*FBLN5*	*Fbln5*
fbln7	fibulin 7	100000083	13	*FBLN7*	*Fbln7*
fbn2a	fibrillin 2a	558217	10	*FBN2*	*Fbn2*
fkbp1aa	FKBP prolyl isomerase 1Aa	335335	8	*FKBP1A*	*Fkbp1a*
fkbp1ab	FKBP prolyl isomerase 1Ab	449552	23	*FKBP1A*	*Fkbp1a*
fkbp1b	FKBP prolyl isomerase 1B	393785	20	*FKBP1B*	*Fkbp1b*
fkbp2	FKBP prolyl isomerase 2	447939	21	*FKBP2*	*Fkbp2*
fkbp3	FKBP prolyl isomerase 3	368863	20	*FKBP3*	*Fkbp3*
fkbp4	FKBP prolyl isomerase 4	321795	18	*FKBP4*	*Fkbp4*
fkbp5	FKBP prolyl isomerase 5	368924	6	*FKBP5*	*Fkbp5*
fkbp6	FKBP prolyl isomerase 6	402839	5	*FKBP6*	*Fkbp6*
fkbp7	FKBP prolyl isomerase 7	368498	9	*FKBP7*	*Fkbp7*
fkbp8	FKBP prolyl isomerase 8	393858	22	*FKBP8*	*Fkbp8*
fkbp9	FKBP prolyl isomerase 9	445126	19	*FKBP9*	*Fkbp9*
fkbp10a	FKBP prolyl isomerase 10a	799903	19	*FKBP10*	*Fkbp10*
fkbp10b	FKBP prolyl isomerase 10b	324381	12	*FKBP10*	*Fkbp10*
fkbp11	FKBP prolyl isomerase 11	368823	6	*FKBP11*	*Fkbp11*
fkbp14	FKBP prolyl isomerase 14	445077	16	*FKBP14*	*Fkbp14*
fkbpl	FKBP prolyl isomerase like	100149636	23	*FKBPL*	*Fkbpl*
ghitm	growth hormone inducible transmembrane protein	393563	12	*GHITM*	*Ghitm*
gpat3	glycerol-3-phosphate acyltransferase 3	436958	21	*GPAT3*	*Gpat3*
gpat4	glycerol-3-phosphate acyltransferase 4	678522	10	*GPAT4*	*Gpat4*
gria1a	glutamate receptor, ionotropic, AMPA 1a	798689	14	*GRIA1*	*Gria1*
gria1b	glutamate receptor, ionotropic, AMPA 1b	403044	21	*GRIA1*	*Gria1*
gria2a	glutamate receptor, ionotropic, AMPA 2a	170450	1	*GRIA2*	*Gria2*
gria2b	glutamate receptor, ionotropic, AMPA 2b	170451	14	*GRIA2*	*Gria2*
gria3a	glutamate receptor, ionotropic, AMPA 3a	170452	5	*GRIA3*	*Gria3*
gria3b	glutamate receptor, ionotropic, AMPA 3b	368416	14	*GRIA3*	*Gria3*
gria4a	glutamate receptor, ionotropic, AMPA 4a	407735	15	*GRIA4*	*Gria4*

gria4b	glutamate receptor, ionotropic, AMPA 4b	336069	21	*GRIA4*	*Gria4*
grin1a	glutamate receptor, ionotropic, N-methyl D-aspartate 1a	767745	21	*GRIN1*	*Grin1*
grin1b	glutamate receptor, ionotropic, N-methyl D-aspartate 1b	100005675	5	*GRIN1*	*Grin1*
grin2aa	glutamate receptor, ionotropic, N-methyl D-aspartate 2A, a	563297	3	*GRIN2A*	*Grin2a*
grin2ab	glutamate receptor, ionotropic, N-methyl D-aspartate 2A, b	570493	1	*GRIN2A*	*Grin2a*
grin2bb	glutamate receptor, ionotropic, N-methyl D-aspartate 2B, genome duplicate b	559976	1	*GRIN2B*	*Grin2b*
grin2cb	glutamate receptor, ionotropic, N-methyl D-aspartate 2Cb	100333648	12	*GRIN2C*	*Grin2c*
grin2da	glutamate receptor, ionotropic, N-methyl D-aspartate 2D, a	449864	19	*GRIN2D*	*Grin2d*
grin3a	glutamate receptor, ionotropic, N-methyl-D-aspartate 3A	564832	10	*GRIN3A*	*Grin3a*
grin3ba	glutamate receptor, ionotropic, N-methyl-D-aspartate 3Ba	566411	2	*GRIN3B*	*Grin3b*
grin3bb	glutamate receptor, ionotropic, N-methyl-D-aspartate 3Bb	100333101	11	*GRIN3B*	*Grin3b*
grinaa	glutamate receptor, ionotropic, N-methyl D-aspartate-associated protein 1a	724005	19	*GRINA*	*Grina*
grinab	glutamate receptor, ionotropic, N-methyl D-aspartate-associated protein 1b	394183	16	*GRINA*	*Grina*
hgfb	hepatocyte growth factor b	100333596	18	*HGF*	*Hgf*
hp	haptoglobin	566096	7	*HP*	*Hp*
hpca	hippocalcin	393696	19	*HPCA*	*Hpca*
hpcal1	hippocalcin-like 1	394139	20	*HPCAL1*	*Hpcal1*
hpcal4	hippocalcin-like 4	564909	19	*HPCAL4*	*Hpcal4*
hrc	histidine rich Ca^{2+}binding protein	553296	12	*HRC*	*Hrc*
itpr1a	inositol 1,4,5-trisphosphate receptor, type 1a	567611	6	*ITPR1*	*Itpr1*
itpr1b	inositol 1,4,5-trisphosphate receptor, type 1b	100149388	11	*ITPR1*	*Itpr1*
itpr2	inositol 1,4,5-trisphosphate receptor, type 2	562585	18	*ITPR2*	*Itpr2*
itpr3	inositol 1,4,5-trisphosphate receptor, type 3	794666	8	*ITPR3*	*Itpr3*
itsn1	intersectin 1 (SH3 domain protein)	368504	1	*ITSN1*	*Itsn1*
itsn2a	intersectin 2a	566412	20	*ITSN2*	*Itsn2*
itsn2b	intersectin 2b	445153	17	*ITSN2*	*Itsn2*
kcnip1a	Kv channel interacting protein 1 a	100008068	-	*KCNIP1*	*Kcnip1*
kcnip1b	Kv channel interacting protein 1 b	494089	10	*KCNIP1*	*Kcnip1*
kcnip2	Kv channel interacting protein 2	100003947	22	*KCNIP2*	*Kcnip2*
kcnip3a	Kv channel interacting protein 3a, calsenilin	393792	8	*KCNIP3*	*Kcnip3*
kcnip3b	Kv channel interacting protein 3b, calsenilin	393755	10	*KCNIP3*	*Kcnip3*
kcnma1a	potassium large conductance Ca^{2+}-activated channel, subfamily M, alpha member 1a	568554	13	*KCNMA1*	*Kcnma1*
kcnma1b	potassium large conductance Ca^{2+}-activated channel, subfamily M, alpha member 1b	798583	12	*KCNMA1*	*Kcnma1*
kcnmb2	potassium large conductance Ca^{2+}-activated channel, subfamily M, beta member 2	541398	2	*KCNMB2*	*Kcnmb2*
kcnn1a	potassium intermediate/small conductance Ca^{2+}-activated channel, subfamily N, member 1a	563352	2	*KCNN1*	*Kcnn1*
kcnn1b	potassium intermediate/small conductance Ca^{2+}-activated channel, subfamily N, member 1b	570401	8	*KCNN1*	*Kcnn1*
kcnn3	potassium intermediate/small conductance Ca^{2+}-activated channel, subfamily N, member 3	570138	16	*KCNN3*	*Kcnn3*
kcnn4	potassium intermediate/small conductance Ca^{2+}-activated channel, subfamily N, member 4	568363	16	*KCNN4*	*Kcnn4*
kcnt1	potassium channel, subfamily T, member 1	100004419	21	*KCNT1*	*Kcnt1*
kcnt2	potassium channel, subfamily T, member 2	568327	2	*KCNT2*	*Kcnt2*
lats1	large tumor suppressor kinase 1	553164	20	*LATS1*	*Lats1*
lats2	large tumor suppressor kinase 2	567674	9	*LATS2*	*Lats2*
letm1	leucine zipper-EF-hand containing transmembrane protein 1	570745	13	*LETM1*	*Letm1*
letm2	leucine zipper-EF-hand containing transmembrane protein 2	100006201	10	*LETM2*	*Letm2*
letmd1	LETM1 domain containing 1	567014	23	*LETMD1*	*Letmd1*

lpcat1	lysophosphatidylcholine acyltransferase 1	555969	19	*LPCAT1*	*Lpcat1*
lpcat2	lysophosphatidylcholine acyltransferase 2	553683	7	*LPCAT2*	*Lpcat2*
lpcat3	lysophosphatidylcholine acyltransferase 2	555566	16	*LPCAT3*	*Lpcat3*
lpcat4	lysophosphatidylcholine acyltransferase 4	327566	7	*LPCAT4*	*Lpcat4*
ltbp1	latent transforming growth factor beta binding protein 1	562072	17	*LTBP1*	*Ltbp1*
ltbp3	latent transforming growth factor beta binding protein 3	562912	5	*LTBP3*	*Ltbp3*
ltbp4	latent transforming growth factor beta binding protein 4	100333499	18	*LTBP4*	*Ltbp4*
mast1a	microtubule associated serine/threonine kinase 1a	796739	1	*MAST1*	*Mast1*
mast1b	microtubule associated serine/threonine kinase 1b	796144	3	*MAST1*	*Mast1*
mast2	microtubule associated serine/threonine kinase 2	100001267	6	*MAST2*	*Mast2*
mast3a	microtubule associated serine/threonine kinase 3a	100534685	2	*MAST3*	*Mast3*
mast3b	microtubule associated serine/threonine kinase 3b	100535762	11	*MAST3*	*Mast3*
mast4	microtubule associated serine/threonine kinase family member 4	100149322	5	*MAST4*	*Mast4*
mastl	microtubule associated serine/threonine kinase-like	445215	24	*MASTL*	*Mastl*
mcu	mitochondrial Ca^{2+}uniporter	768182	13	*MCU*	*Mcu*
mcur1 (zgc:165532)	mitochondrial Ca^{2+}uniporter regulator 1	550408	7	*MCUR1*	*Mcur1*
micu1	mitochondrial Ca^{2+}uptake 1	561210	13	*MICU1*	*Micu1*
micu2	mitochondrial Ca^{2+}uptake 2	548335	5	*MICU1*	*Micu1*
micu3a	mitochondrial Ca^{2+}uptake family, member 3a	559552	1	*MICU3*	*Micu3*
micu3b	mitochondrial Ca^{2+}uptake family, member 3a	100536173	14	*MICU3*	*Micu3*
myl10	myosin, light chain 10, regulatory	550569	10	*MYL10*	*Myl10*
myl2a	myosin, light chain 2a, regulatory, cardiac, slow	100537244	5	*MYL2*	*Myl2*
myl2b	myosin, light chain 2b, regulatory, cardiac, slow	677750	8	*MYL2*	*Myl2*
myl6	myosin, light chain 6, alkali, smooth muscle and non-muscle	798632	6	*MYL6*	*Myl6*
myl7	myosin, light chain 7, regulatory	30592	8	*MYL7*	*Myl7*
myl9a	myosin, light chain 9a, regulatory	450006	23	*MYL9*	*Myl9*
myl9b	myosin, light chain 9b, regulatory	406493	6	*MYL9*	*Myl9*
mylpfa	myosin light chain, phosphorylatable, fast skeletal muscle a	30429	3	*MYLPF*	*Mylpf*
mylpfb	myosin light chain, phosphorylatable, fast skeletal muscle b	447930	12	*MYLPF*	*Mylpf*
ncs1a	neuronal Ca^{2+}sensor 1a	393437	5	*NCS1*	*Ncs1*
ncs1b	neuronal Ca^{2+}sensor 1b	553174	8	*NCS1*	*Ncs1*
necab1	N-terminal EF-hand Ca^{2+}binding protein 1	550546	19	*NECAB1*	*Necab1*
necab2	N-terminal EF-hand Ca^{2+}binding protein 2	565258	18	*NECAB2*	*Necab2*
necab3	N-terminal EF-hand Ca^{2+}binding protein 3	101887165	6	*NECAB3*	*Necab3*
nell2a	neural EGFL like 2a	790930	25	*NELL2*	*Nell2*
nell2b	neural EGFL like 2b	336082	4	*NELL2*	*Nell2*
nid2a	nidogen 2a (osteonidogen)	322921	17	*NID2*	*Nid2*
nid2b	nidogen 2b (osteonidogen)	570436	20	*NID2*	*Nid2*
nox5	NADPH oxidase, EF-hand Ca^{2+}binding domain 5	100149800	25	*NOX5*	-
nucb1	nucleobindin 1	751753	3	*NUCB1*	*Nucb1*
nucb2a	nucleobindin 2a	373078	7	*NUCB2*	*Nucb2*
nucb2b	nucleobindin 2b	326050	18	*NUCB2*	*Nucb2*
orai1a	ORAI Ca^{2+}release-activated Ca^{2+}modulator 1a	566671	8	*ORAI1*	*Orai1*
orai1b	ORAI Ca^{2+}release-activated Ca^{2+}modulator 1b	402892	10	*ORAI1*	*Orai1*
orai2	ORAI Ca^{2+}release-activated Ca^{2+}modulator 2	555612	5	*ORAI2*	*Orai2*
ormdl1	ORMDL sphingolipid biosynthesis regulator 1	368632	9	*ORMDL1*	*Ormdl1*
ormdl2	ORMDL sphingolipid biosynthesis regulator 2	677753	23	*ORMDL2*	*Ormdl2*
ormdl3	ORMDL sphingolipid biosynthesis regulator 3	450067	12	*ORMDL3*	*Ormdl3*
pdpk1a	3-phosphoinositide dependent protein kinase 1a	768202	3	*PDPK1*	*Pdpk1*
pdpk1b	3-phosphoinositide dependent protein kinase 1b	403000	24	*PDPK1*	*Pdpk1*
pdzd8	PDZ domain containing 8	561279	17	*PDZD8*	*Pdzd8*

pkn1a	protein kinase N1a	560840	1	*PKN1*	*Pkn1*
pkn1b	protein kinase N1b	100007897	3	*PKN1*	*Pkn1*
pkn2	protein kinase N2	571965	2	*PKN2*	*Pkn2*
pkn3	protein kinase N3	323182	5	*PKN3*	*Pkn3*
plcb3	phospholipase C, beta 3 (phosphatidylinositol-specific)	100001532	7	*PLCB3*	*Plcb3*
plcg1	phospholipase C, gamma 1	373867	23	*PLCG1*	*Plcg1*
plcg2	phospholipase C, gamma 2	561747	18	*PLCG2*	*Plcg2*
plch1	phospholipase C, eta 1	566479	18	*PLCH1*	*Plch1*
plch2a	phospholipase C, eta 2a	100334231	8	*PLCH2*	*Plch2*
plch2b	phospholipase C, eta 2b	560919	11	*PLCH2*	*Plch2*
plcl1	phospholipase C like 1	563105	9	*PLCL1*	*Plcl1*
plcl2	phospholipase C like 2	100334276	16	*PLCL2*	*Plcl2*
ppef1	protein phosphatase, EF-hand Ca^{2+}binding domain 1	559132	11	*PPEF1*	*Ppef1*
ppef2b	protein phosphatase with EF-hand domain 2b	100537436	21	*PPEF2*	*Ppef2*
ppp1caa	protein phosphatase 1, catalytic subunit, alpha isozyme a	407980	3	*PPP1CA*	*Ppp1ca*
ppp1cab	protein phosphatase 1, catalytic subunit, alpha isozyme b	327301	12	*PPP1CA*	*Ppp1ca*
ppp1cb	protein phosphatase 1, catalytic subunit, beta isozyme	100003223	17	*PPP1CB*	*Ppp1cb*
ppp1cbl	protein phosphatase 1, catalytic subunit, beta isoform, like	334597	7	*PPP1CB*	*Ppp1cb*
ppp1cc	protein phosphatase 1, catalytic subunit, gamma isozyme	571753	5	*PPP1CC*	*Ppp1cc*
prkcba	protein kinase C, beta a	100006675	1	*PRKCB*	*Prkcb*
prkcbb	protein kinase C, beta b	393953	3	*PRKCB*	*Prkcb*
prkcda	protein kinase C, delta a	334571	6	*PRKCD*	*Prkcd*
prkcdb	protein kinase C, delta b	100150844	11	*PRKCD*	*Prkcd*
prkcea	protein kinase C, epsilon a	100005075	12	*PRKCE*	*Prkce*
prkceb	protein kinase C, epsilon b	100332055	13	*PRKCE*	*Prkce*
prkcg	protein kinase C, gamma	100003514	16	*PRKCG*	*Prkcg*
prkcha	protein kinase C, eta, a	100144561	13	*PRKCH*	*Prkch*
prkchb	protein kinase C, eta, b	556591	20	*PRKCH*	*Prkch*
prkci	protein kinase C, iota	117507	2	*PRKCI*	*Prkci*
prkcq	protein kinase C, theta	555521	4	*PRKCQ*	*Prkcq*
prkcz	protein kinase C, zeta	555737	8	*PRKCZ*	*Prkcz*
psen1	presenilin 1	30221	17	*PSEN1*	*Psen1*
psen2	presenilin 2	58026	1	*PSEN2*	*Psen2*
pvalb1	parvalbumin 1	402805	3	-	-
pvalb2	parvalbumin 2	58028	12	-	-
pvalb3	parvalbumin 3	100000771	12	-	-
pvalb4	parvalbumin 4	337731	3	-	-
pvalb5	parvalbumin 5	335623	3	-	-
pvalb6	parvalbumin 6	402806	3	*PVALB*	*Pvalb*
pvalb7	parvalbumin 7	402807	22	*PVALB*	*Pvalb*
pvalb8	parvalbumin 8	360208	3	-	-
pvalb9	parvalbumin 9	360209	12	-	-
rcn1	reticulocalbin 1, EF-hand Ca^{2+}binding domain	562671	7	*RCN1*	*Rcn1*
rcn2	reticulocalbin 2	571340	18	*RCN2*	*Rcn2*
rcn3	reticulocalbin 3, EF-hand Ca^{2+}binding domain	415248	3	*RCN3*	*Rcn3*
reps1	RALBP1 associated Eps domain containing 1	564535	20	*REPS1*	*Reps1*
reps2	RALBP1 associated Eps domain containing 2	101882936	23	*REPS2*	*Reps2*
ryr1a	ryanodine receptor 1a (skeletal)	798565	10	*RYR1*	*Ryr1*
ryr1b	ryanodine receptor 1b (skeletal)	570245	18	*RYR1*	*Ryr1*
ryr2a	ryanodine receptor 2a (cardiac)	100126126	12	*RYR2*	*Ryr2*
ryr2b	ryanodine receptor 2b (cardiac)	568463	17	*RYR2*	*Ryr2*
ryr3	ryanodine receptor 3	561350	20	*RYR3*	*Ryr3*

s100a1	S100 Ca^{2+}binding protein A1	448856	16	*S100A1*	*S100a1*
s100a10a	S100 Ca^{2+}binding protein A10a	449788	19	*S100A10*	*S100a10*
s100a10b	S100 Ca^{2+}binding protein A10b	406276	16	*S100A10*	*S100a10*
s100a11	S100 Ca^{2+}binding protein A11	100307070	19	*S100A11*	*S100a11*
s100b	S100 Ca^{2+}binding protein, beta (neural)	436825	22	*S100B*	*S100b*
s100s	S100 Ca^{2+}binding protein S	445481	19	-	-
s100t	S100 Ca^{2+}binding protein T	541368	16	-	-
s100u	S100 Ca^{2+}binding protein U	563060	19	-	-
s100v2	S100 Ca^{2+}binding protein V2	336965	19	-	-
s100w	S100 Ca^{2+}binding protein W	569958	16	-	-
s100z	S100 Ca^{2+}binding protein Z	554089	21	*S100Z*	*S100z*
saraf	store-operated Ca^{2+}entry-associated regulatory factor	767649	1	*SARAF*	*Saraf*
scgn	secretagogin, EF-hand Ca^{2+}binding protein	573010	16	*SCGN*	*Scgn*
sgca	sarcoglycan, alpha	557170	12	*SGCA*	*Sgca*
sgcb	sarcoglycan, beta (dystrophin-associated glycoprotein)	559788	20	*SGCB*	*Sgcb*
sgcd	sarcoglycan, delta (dystrophin-associated glycoprotein)	324961	21	*SGCD*	*Sgcd*
sgce	sarcoglycan, epsilon	368230	19	*SGCE*	*Sgce*
sgcg	sarcoglycan, gamma	445292	15	*SGCG*	*Sgcg*
sgcz	sarcoglycan, zeta	100334126	1	*SGCZ*	*Sgcz*
sgk1	serum/glucocorticoid regulated kinase 1	324140	23	*SGK1*	*SGK1*
sgk2a	serum/glucocorticoid regulated kinase 2a	570956	23	*SGK2*	*Sgk2*
sgk2b	serum/glucocorticoid regulated kinase 2b	559050	16	*SGK2*	*Sgk2*
sgk3	serum/glucocorticoid regulated kinase family, member 3	564523	24	*SGK3*	*Sgk3*
sigmar1	sigma non-opioid intracellular receptor 1	393952	10	*SIGMAR1*	*Sigmar1*
slc24a1	solute carrier family 24 (sodium/potassium/Ca^{2+}exchanger), member 1	558299	18	*SLC24A1*	*Slc24a1*
slc24a2	solute carrier family 24 (sodium/potassium/Ca^{2+}exchanger), member 2	751686	1	*SLC24A2*	*Slc24a2*
slc24a3	solute carrier family 24 (sodium/potassium/Ca^{2+}exchanger), member 3	557196	13	*SLC24A3*	*Slc24a3*
slc24a4a	solute carrier family 24 (sodium/potassium/Ca^{2+}exchanger), member 4a	550467	17	*SLC24A4*	*Slc24a4*
slc24a4b	solute carrier family 24 (sodium/potassium/Ca^{2+}exchanger), member 4b	100334346	20	*SLC24A4*	*Slc24a4*
slc24a5	solute carrier family 24 (sodium/potassium/Ca^{2+}exchanger), member 5	570312	18	*SLC24A5*	*Slc24a5*
slc24a6a	solute carrier family 24 (sodium/potassium/Ca^{2+}exchanger), member 6a	561209	14	-	-
slc25a12	solute carrier family 25 (aspartate/glutamate carrier), member 12	337675	6	*SLC25A12*	*Slc25a12*
slc25a19	solute carrier family 25 (mitochondrial thiamine pyrophosphate carrier), member 19	403024	3	*SLC25A19*	*Slc25a19*
slc25a23a	solute carrier family 25 (mitochondrial carrier; phosphate carrier), member 23a	561939	1	*SLC25A23*	*Slc25a23*
slc25a23b	solute carrier family 25 (mitochondrial carrier; phosphate carrier), member 23b	561087	3	*SLC25A23*	*Slc25a23*
slc25a24	solute carrier family 25 (mitochondrial carrier; phosphate carrier), member 24	447867	20	*SLC25A24*	*Slc25a2-*
slc25a24l	solute carrier family 25 (mitochondrial carrier; phosphate carrier), member 24, like	678557	2	*SLC25A25*	*Slc25a25*
slc25a25a	solute carrier family 25 (mitochondrial carrier; phosphate carrier), member 25a	406541	8	*SLC25A25*	*Slc25a25*
slc25a25b	solute carrier family 25 (mitochondrial carrier; phosphate carrier), member 25b	569227	5	*SLC25A25*	*Slc25a25*
slc25a28	solute carrier family 25 (mitochondrial iron transporter), member 28	100535877	13	*SLC25A28*	*Slc25a28*
slc25a37	solute carrier family 25 (mitochondrial iron transporter), member 37	387000	8	*SLC25A37*	*Slc25a37*
slc25a39	solute carrier family 25, member 39	393458	3	*SLC25A39*	*Slc25a39*
slc25a42	solute carrier family 25, member 42	751743	2	*SLC25A42*	*Slc25a42*
slc25a43	solute carrier family 25, member 43	796731	14	*SLC25A43*	*Slc25a43*
slc8a1a	solute carrier family 8 (sodium/Ca^{2+}exchanger), member 1a	553489	11	*SLC8A1*	*Slc8a1*
slc8a1b	solute carrier family 8 (sodium/Ca^{2+}exchanger), member 1b	797199	17	*SLC8A1*	*Slc8a1*
slc8a2a	solute carrier family 8 (sodium/Ca^{2+}exchanger), member 2a	569480	5	*SLC8A2*	*Slc8a2*

slc8a2b	solute carrier family 8 (sodium/Ca^{2+}exchanger), member 2b	567212	15	*SLC8A2*	*Slc8a2*
slc8a3	solute carrier family 8 (sodium/Ca^{2+}exchanger), member 3	560561	13	*SLC8A3*	*Slc8a3*
slc8a4a	solute carrier family 8 (sodium/Ca^{2+}exchanger), member 4a	568512	21	-	-
slc8a4b	solute carrier family 8 (sodium/Ca^{2+}exchanger), member 4b	557499	7	-	-
slc8b1	solute carrier family 8 (sodium/lithium/Ca^{2+}exchanger), member B1	560601	7	*SLC8B1*	*Slc8b1*
smdt1b	single-pass membrane protein with aspartate-rich tail 1b	100000862	1	*SMDT1*	*Smdt1*
smoc1	SPARC related modular Ca^{2+}binding 1	100529120	17	*SMOC1*	*Smoc1*
smoc2	SPARC related modular Ca^{2+}binding 2	266988	13	*SMOC2*	*SMOC2*
srl	sarcalumenin	449670	3	*SRL*	*Srl*
ssr1	signal sequence receptor, alpha	373100	24	*SSR1*	*Ssr1*
stim1a	stromal interaction molecule 1a	556360	15	*STIM1*	*Stim1*
stim1b	stromal interaction molecule 1b	100005700	21	*STIM1*	*Stim1*
stim2a	stromal interaction molecule 2a	100334881	1	*STIM2*	*Stim2*
stim2b	stromal interaction molecule 2b	563901	7	*STIM2*	*Stim2*
stimate	STIM activating enhance	393439	8	*STIMATE*	*Stimate*
stk32a	serine/threonine kinase 32A	100330817	21	*STK32A*	*Stk32a*
stk38a	serine/threonine kinase 38a	406765	8	*STK38*	*Stk38*
stk38b	serine/threonine kinase 38b	100332206	22	*STK38*	*Stk38*
stk38l	serine/threonine kinase 38 like	393957	4	*STK38L*	*Stk38l*
tdgf1	teratocarcinoma-derived growth factor 1	30304	10	*TDGF1*	*Tdgf1*
tenm1	teneurin transmembrane protein 1	563094	5	*TENM1*	*Tenm1*
tenm3	teneurin transmembrane protein 3	30155	1	-	-
tenm4	teneurin transmembrane protein 4	30156	15	*TENM4*	*Tenm4*
thbd	thrombomodulin	108180329	20	*THBD*	*Thbd*
tmbim1a	transmembrane BAX inhibitor motif containing 1a	449819	6	*TMBIM1*	*Tmbim1*
tmbim4	transmembrane BAX inhibitor motif containing 4	406412	4	*TMBIM4*	*Tmbim4*
tmx1	thioredoxin-related transmembrane protein 1	323607	13	*TMX1*	*Tmx1*
tnnc1a	troponin C type 1a (slow)	353247	23	*TNNC1*	*Tnnc1*
tnnc1b	troponin C type 1b (slow)	415175	23	*TNNC1*	*Tnnc1*
tpcn1	two pore segment channel 1	567534	5	*TPCN1*	*Tpcn1*
tpcn2	two pore segment channel 2	777614	7	*TPCN2*	*Tpcn2*
tpcn3	two pore segment channel 3	557460	1	-	-
trpa1a	transient receptor potential cation channel, subfamily A, member 1a	474351	2	*TRPA1*	*Trpa1*
trpa1b	transient receptor potential cation channel, subfamily A, member 1b	474353	24	*TRPA1*	*Trpa1*
trpc1	transient receptor potential cation channel, subfamily C, member 1	570841	24	*TRPC1*	*Trpc1*
trpc2a	transient receptor potential cation channel subfamily C member 2a	559914	15	-	*Trpc2*
trpc2b	transient receptor potential cation channel subfamily C member 2b	562120	21	*TRPC2*	*Trpc2*
trpc3	transient receptor potential cation channel, subfamily C, member 3	101887082	14	*TRPC3*	*Trpc3*
trpc4a	transient receptor potential cation channel, subfamily C, member 4a	102725537	10	*TRPC4*	*Trpc4*
trpc4b	transient receptor potential cation channel, subfamily C, member 4b	102725536	15	*TRPC4*	*Trpc4*
trpc5a	transient receptor potential cation channel, subfamily C, member 5a	557456	1	*TRPC5*	*Trpc5*
trpc5b	transient receptor potential cation channel, subfamily C, member 5b	559586	21	*TRPC5*	*Trpc5*
trpc6a	transient receptor potential cation channel, subfamily C, member 6a	563989	21	*TRPC6*	*Trpc6*
trpc6b	transient receptor potential cation channel, subfamily C, member 6b	102725535	18	*TRPC6*	*Trpc6*
trpc7a	transient receptor potential cation channel, subfamily C, member 7a	567566	10	*TRPC7*	*Trpc7*
trpc7b	transient receptor potential cation channel, subfamily C, member 7b	102725540	14	*TRPC7*	*Trpc7*
trpm1a	transient receptor potential cation channel, subfamily M, member 1a	791196	7	*TRPM1*	*Trpm1*
trpm1b	transient receptor potential cation channel, subfamily M, member 1b	569946	25	*TRPM1*	*Trpm1*
trpm2	transient receptor potential cation channel, subfamily M, member 2	799412	9	*TRPM2*	*Trpm2*
trpm3	transient receptor potential cation channel, subfamily M, member 3	565807	5	*TRPM3*	*Trpm3*
trpm4a	transient receptor potential cation channel, subfamily M, member 4a	100004946	3	*TRPM4*	*Trpm4*
trpm4b.1	transient receptor potential cation channel, subfamily M, member 4b, tandem duplicate 1	102659287	12	*TRPM4*	*Trpm4*

trpm4b.2	transient receptor potential cation channel, subfamily M, member 4b, tandem duplicate 2	799647	12	*TRPM4*	*Trpm4*
trpm5	transient receptor potential cation channel, subfamily M, member 5	557722	7	*TRPM5*	*Trpm5*
trpm6	transient receptor potential cation channel, subfamily M, member 6	100149353	5	*TRPM6*	*Trpm6*
trpm7	transient receptor potential cation channel, subfamily M, member 7	280653	18	*TRPM7*	*Trpm7*
trpn1	transient receptor potential cation channel, subfamily N, member 1	368273	19	-	-
trpv1	transient receptor potential cation channel, subfamily V, member 1	561195	5	*TRPV1*	*Trpv1*
trpv4	transient receptor potential cation channel, subfamily V, member 4	557850	5	*TRPV4*	*Trpv4*
trpv6	transient receptor potential cation channel, subfamily V, member 6	100000322	16	*TRPV6*	*Trpv6*
ttc9b	tetratricopeptide repeat domain 9B	559732	18	*TTC9B*	*Ttc9b*
ttc9c	tetratricopeptide repeat domain 9C	393235	14	*TTC9C*	*Ttc9c*
tusc2a	tumor suppressor 2, mitochondrial Ca^{2+}regulator a	768156	6	*TUSC2*	*Tusc2*
tusc2b	tumor suppressor 2, mitochondrial Ca^{2+}regulator b	436675	22	*TUSC2*	*Tusc2*
vdac1	voltage-dependent anion channel 1	334582	21	*VDAC1*	*Vdac1*
vdac2	voltage-dependent anion channel 2	322126	13	*VDAC2*	*Vdac2*
vdac3	voltage-dependent anion channel 2	406529	8	*VDAC3*	*Vdac3*
vwde	von Willebrand factor D and EGF domains	767804	15	*VWDE*	*Vwde*
wdr74	WD repeat domain 74	558996	10	*WDR74*	*Wdr74*
wfs1a	Wolfram syndrome 1a (wolframin)	566876	1	*WFS1*	*Wfs1*
wif1	wnt inhibitory factor 1	30476	4	*WIF1*	*Wif1*

Table S3 Zebrafish brain Calcium Toolkit

The list of 444 genes from zebrafish CaTK detected by RNA-Seq in brain samples. Expression data and zebrafish mutant phenotype sections contain references to appropriate publications if available. Adult brain and larvae head expression contain data regarding the relative level of mRNA of 77 genes (in bold) estimated using RT-PCR arrays expressed as VERY LOW, LOW, MEDIUM or HIGH (this dataset was submitted to the Gene Expression Omnibus database). “-“ stands for no data available. The genes in bold and underlined are those used to validate the RT-PCR assay. RPM (Reads Per Million Reads) values obtain from RNA-seq experiment are placed in the brackets.

gene symbol	human ortholog	location	biological process	molecular function	zebrafish mutant phenotype	expression data (RPM)	
						adult brain expression	larvae head expression
aif1l	*AIF1L*	C	actin filament bundle assembly, ruffle assembly	actin filament binding, Ca^{2+} binding	-	**(44.38)** [1]	
akt1	*AKT1*	PM/C/N	intracellular signal transduction, negative regulation of neuron differentiation, protein phosphorylation	ATP binding, protein serine/threonine kinase activity, transferase activity	malformed brain, edematous pericardium, bent trunk, decreased number of neuroblast [2]	**(5.84)** [2]	
akt2	*AKT2*	PM/C/N	bone development, glucose homeostasis, intracellular signal transduction, negative regulation of apoptotic process, peptidyl-serine phosphorylation, positive regulation of glucose import, protein kinase B signaling		decreased body length and weight, abnormal fins development, increased amount of blood glucose, decreased rate of glucose import, decreased *ins* and *apoa4a* expression, increased *mlxipl* expression, increased apoptosis [3,4]	**(57.93)** [4]	
akt3b	*AKT3*	PM/C/N	protein phosphorylation		-	**(39.5)**	
ano1	*ANO1*	PM	cellular response to heat, chloride transmembrane transport, positive regulation of insulin secretion involved in cellular response to glucose stimulus	protein dimerization activity	-	**(50.59)**	
anxa11a	*ANXA11*	C	cytokinetic process, phagocytosis, response to Ca^{2+}	S100 protein binding, Ca^{2+} binding, Ca^{2+}-dependent phospholipid binding, Ca^{2+}-dependent protein binding, phosphatidylethanolamine binding	-	**(09.03)** [1,5]	
anxa11b	*ANXA11*	C			-	**(42.17)** [1,5]	
anxa13	*ANXA13*	PM/N	positive regulation of Golgi to plasma membrane protein transport	Ca^{2+} binding, Ca^{2+}-dependent phospholipid binding, phosphatidylglycerol binding, phosphatidylserine binding	-	**(51.17)** [6]	
anxa13l	*ANXA13*	PM/N			-	**(23.89)** [7]	

anxa1a	*ANXA1*	EM/PM	DNA duplex unwinding, G protein-coupled receptor signaling pathway, coupled to cyclic nucleotide second messenger, actin cytoskeleton reorganization, alpha-beta T cell differentiation, arachidonic acid secretion, cell surface receptor signaling pathway, cellular response to glucocorticoid stimulus, cellular response to hydrogen peroxide, cellular response to vascular endothelial growth factor stimulus, endocrine pancreas development, fin regeneration, gliogenesis, granulocyte and monocyte chemotaxis, hepatocyte differentiation, inflammatory response, insulin secretion, keratinocyte differentiation, myoblast migration involved in skeletal muscle regeneration, negative regulation of T-helper 2 cell differentiation, negative regulation of exocytosis, negative regulation of interleukin-8 secretion, negative regulation of phospholipase A2 activity, neutrophil clearance, phagocytosis	DNA/DNA annealing activity, Ca^{2+} binding, Ca^{2+}-dependent phospholipid binding, Ca^{2+}-dependent protein binding, double-stranded DNA-dependent ATPase activity, phospholipase A2 inhibitor activity, protein homodimerization activity	-	[5,7] **(2.26)**
anxa1b	*ANXA1*	EM/PM			-	[5,7,8] **(0.27)**
anxa1c	*ANXA1*	EM/PM			-	[1] **(0.14)**
anxa2a	*ANXA2*	EM/PM	-	Ca^{2+} binding, Ca^{2+}-dependent phospholipid binding, cytoskeletal protein binding, phospholipase inhibitor activity	-	[5,7] **(21.12)**
anxa4	*ANXA4*	PM/N/C	-	Ca^{2+} binding, Ca^{2+}-dependent phospholipid binding	increased apoptotic process in the liver [9]	[9] **(3.35)**
anxa5a	*ANXA5*	EM/PM/C	negative regulation of coagulation, regulation of cell motility		-	[10,11] **(0.41)**
anxa5b	*ANXA5*	EM/PM/C			-	[1,12,13] **(2.26)**

anxa6	*ANXA6*	C	apoptotic signaling pathway, biomineral tissue development, Ca^{2+} import, growth plate cartilage chondrocyte differentiation, mitochondrial Ca^{2+} homeostasis, negative regulation of sequestering of Ca^{2+}, neural crest cell migration, plasma membrane repair, protein homooligomerization, regulation of muscle contraction	GTP binding, actin filament binding, Ca^{2+} channel activity, Ca^{2+} binding, Ca^{2+}-dependent phospholipid binding, Ca^{2+}-dependent protein binding, cholesterol binding, chondroitin sulfate binding, enzyme binding, heparin binding, ligand-gated ion channel activity, phosphatidylserine binding, protein homodimerization activity, protein-containing complex binding, signaling receptor activity	muscle cell myofibril damaged, muscle T-tubule misaligned, curved trunk [5]	**(9.46)**	[5,7]
apba1a	***APBA1***	PM	chemical synaptic transmission, protein-containing complex assembly	amyloid-beta binding	-	**LOW (56.07)**	**VERY LOW**
apba1b	***APBA1***	PM	chemical synaptic transmission	amyloid-beta binding	-	**LOW (94.17)**	**VERY LOW**
apba2a	*APBA2*	PM	chemical synaptic transmission	amyloid-beta binding	-	**(58.53)**	
apba2b	***APBA2***	PM	chemical synaptic transmission	amyloid-beta binding	-	**LOW (202.76)**	**VERY LOW**
apbb1	***APBB1***	C	regulation of transcription, DNA-templated	amyloid-beta binding, transcription factor binding	-	**VERY LOW (38.20)**	**VERY LOW**
apbb1ip	*APBB1IP*	C/PM	signal transduction	-	-	**(1.84)**	[7]
apbb2b	***APBB2***	C	regulation of transcription, DNA-templated	amyloid-beta binding, transcription factor binding	-	**MEDIUM (40.42)**	**LOW**
apbb3	***APBB3***	C	-	-	-	**VERY LOW (27.94)**	**VERY LOW**
atox1	***ATOX1***	C	cellular copper ion homeostasis, metal ion transport, response to oxidative stress	copper chaperone activity, metal ion binding	-	**MEDIUM (7.41)**	**HIGH**
atp2a1	*ATP2A1*	ER	Ca^{2+} transmembrane transport, cellular Ca^{2+} homeostasis, negative regulation of muscle contraction, response to mechanical stimulus	Ca^{2+} transporting ATPase activity, proton-exporting ATPase activity, phosphorylative mechanism	abnormal locomotion behavior, skeletal muscle contraction disrupted, thigmotaxis disrupted, fast muscle cell skeletal muscle myofibril disorganized, abnormal notochord, bent trunk, neuromuscular process controlling balance disrupted [14-16]	**(1.36)**	[1,17-19]
atp2a1l	*ATP2A1*	ER	Ca^{2+} transmembrane transport, cellular Ca^{2+} homeostasis	Ca^{2+} transporting ATPase activity, proton-exporting ATPase activity, phosphorylative mechanism	-	**(0.22)**	

atp2a2a	*ATP2A2*	ER	Ca^{2+} transmembrane transport, cellular Ca^{2+} homeostasis, heart looping, regulation of heart contraction	Ca^{2+} transporting ATPase activity, proton-exporting ATPase activity, phosphorylative mechanism	atrium and cardiac ventricule collapsed, decreased atrium and cardiac ventricule contractility, heart contraction decreased rate, heart looping arrested, pericardium edematous, sensory perception of touch disrupted [20]	[7,20] **(74.48)**
atp2a2b	*ATP2A2*	ER	Ca^{2+} transmembrane transport, cellular Ca^{2+} homeostasis		-	[6] **(153.59)**
atp2a3	*ATP2A3*	ER	Ca^{2+} transmembrane transport, cellular Ca^{2+} homeostasis		-	[21] **(188.56)**
atp2b1a	*ATP2B1*	PM	bone mineralization, Ca^{2+} transmembrane transport, cellular Ca^{2+} homeostasis, inner ear receptor cell development, otolith development, posterior lateral line neuromast development, regulation of cytosolic Ca^{2+} concentration, sensory perception of sound, tooth mineralization	Ca^{2+}transporting ATPase activity, PDZ domain binding	abnormal tooth mineralization, posterior lateral and otolith development, disrupted bone mineralization, abnormal sensory perception of sound, integument ionocyte morphology [22-24]	[1,7,17,25] **(207.85)**
atp2b1b	*ATP2B1*	PM	Ca^{2+} transmembrane transport, cellular Ca^{2+} homeostasis, neuron differentiation, regulation of cytosolic Ca^{2+} concentration		-	[25] **(4.76)**
atp2b2	*ATP2B*	PM			-	[25] **(365.62)**
atp2b3a	*ATP2B3*	PM			-	[7,25] **(415.37)**
atp2b3b	*ATP2B3*	PM			-	[25] **(19.51)**
atp2b4	*ATP2B4*	PM			-	[25] **(50.96)**
atp2c1	*ATP2C1*	G	Ca^{2+} transmembrane transport, cellular Ca^{2+} homeostasis, manganese ion homeostasis, manganese ion transport	Ca^{2+} transporting ATPase activity, manganese-transporting ATPase activity, proton-exporting ATPase activity, phosphorylative mechanism	-	[26] **(34.69)**
atp6v0cb	*ATP6V0C*	PM	ATP hydrolysis coupled proton transport, ion transport	proton-transporting ATPase activity, rotational mechanism, voltage-gated Ca^{2+} channel activity	disrupted voltage-gated Ca^{2+} channel activity [27]	[17,27,28] **(148.81)**

baxa	***BAX***	PM/Mt	extrinsic apoptotic signaling pathway in absence of ligand, intrinsic apoptotic signaling pathway in response to DNA damage, mitochondrial fusion, positive regulation of apoptotic process, positive regulation of cysteine-type endopeptidase activity involved in apoptotic process, release of cytochrome c from mitochondria, response to cadmium ion, response to methylmercury	channel activity, protein heterodimerization activity, protein homodimerization activity	-	**[29]** **LOW (15.69)**	**LOW**
baxb	*BAX*	PM/Mt	extrinsic apoptotic signaling pathway in absence of ligand, intrinsic apoptotic signaling pathway in response to DNA damage, mitochondrial fusion, positive regulation of apoptotic process, positive regulation of cysteine-type endopeptidase activity involved in apoptotic process, release of cytochrome c from mitochondria	channel activity, protein heterodimerization activity, protein homodimerization activity	-	[30] **(1.60)**	
cab39	*CAB39*	C	signal transduction by protein phosphorylation	protein kinase activator activity	-	**(22.53)**	
cab39l	*CAB39L*	C	signal transduction by protein phosphorylation	contributes_to protein serine/threonine kinase activity	-	**(72.69)**	
cabp1a	*CABP1*	PM	-	Ca^{2+} binding	-	[7] **(23.54)**	
cabp1b	*CABP1*	PM	-		-	[7,31] **(11.27)**	
cacna1aa	***CACNA1A***	PM	Ca^{2+} transmembrane transport, chemical synaptic transmission, regulation of ion transmembrane transport	voltage-gated Ca^{2+} channel activity	-	**[6]** **LOW (300.33)**	**VERY LOW**
cacna1ab	***CACNA1A***	PM	Ca^{2+} transmembrane transport, chemical synaptic transmission, neuromuscular synaptic transmission, regulation of ion transmembrane transport, swimming behavior, thigmotaxis	high voltage-gated Ca^{2+} channel activity, Ca^{2+} binding	abnormal high voltage-gated Ca^{2+} channel activity, decreased neuromuscular synaptic transmission, abnormal swimming behavior, disrupted thigmotaxis, decreased mobility [14,32,33]	**[32]** **MEDIUM (175.40)**	**VERY LOW**
cacna1ba	*CACNA1B*	PM	Ca^{2+} transmembrane transport, chemical synaptic transmission, regulation of ion transmembrane transport	voltage-gated Ca^{2+} channel activity	-	[34] **(84.54)**	
cacna1bb	***CACNA1B***	PM			-	**VERY LOW**	**VERY LOW**

cacna1c	***CACNA1C***	PM	Ca^{2+} transmembrane transport, embryonic cranial skeleton morphogenesis, heart looping, heart morphogenesis, kidney development, physiological cardiac muscle hypertrophy, regulation of heart contraction	high voltage-gated Ca^{2+} channel activity	decreased volume of cardiac muscle cells, abnormal cardiac ventricle morphology, arrested heart concrations, pronephric duct cystic and dilated, hydrocephalic brain, disrupted determination of heart left/right asymmetry, abnormal mandibular arch skeleton morphology, decreased size of Meckel's cartilage chondrocytes [35-38]	**(220.38)**	[35,39]
cacna1da	***CACNA1D***	PM	Ca^{2+} transmembrane transport, detection of mechanical stimulus involved in sensory perception of sound, neuromuscular process controlling balance, synapse organization		abnormal neuromast hair cell cellular response to mechanical stimulus, neuromast hair cell ribbon synapse morphology and organization, abnormal neuron-neuron synaptic transmission, disrupted posterior lateral line neuromast hair cell development, abnormal larval locomotory behavior [14,40,41]	**(93.97)**	[42-44]
cacna1db	***CACNA1D***	PM	Ca^{2+} transmembrane transport, regulation of ion transmembrane transport	high voltage-gated Ca^{2+} channel activity	-	**VERY LOW (30.97)**	**VERY LOW**
cacna1eb	*CACNA1E*	PM	Ca^{2+} transmembrane transport, chemical synaptic transmission	voltage-gated Ca^{2+} channel activity	-	**(71.33)**	
cacna1fa	*CACNA1F*	PM	T cell homeostasis, Ca^{2+} transmembrane transport, regulation of T cell receptor signaling pathway, synapse organization, visual perception	high voltage-gated Ca^{2+} channel activity	retinal outer plexiform layer decreased thickness, abnormal detection of light stimulus involved in visual perception, abnormal retinal cone cell chemical synaptic transmission, abnormal retinal cone cell cone cell pedicle morphology [45]	**(0.8)**	[45]
cacna1g	***CACNA1G***	PM	Ca^{2+} transmembrane transport, cardiac muscle cell action potential involved in contraction, membrane depolarization during action potential, positive regulation of Ca^{2+}-dependent exocytosis, regulation of heart rate by cardiac conduction	low voltage-gated Ca^{2+} channel activity	-	**LOW (137.38)**	**VERY LOW**
cacna1ha	*CACNA1H*	PM	Ca^{2+} transmembrane transport, membrane depolarization during action potential, neuronal action potential, positive regulation of Ca^{2+} -dependent exocytosis	low voltage-gated Ca^{2+} channel activity	-	**(74.71)**	[1,46]
cacna1hb	*CACNA1H*	PM			-	**(6.91)**	
cacna1i	*CACNA1I*	PM	Ca^{2+} transmembrane transport, flagellated sperm motility, membrane depolarization during action potential, neuronal action potential positive regulation of Ca^{2+} -dependent exocytosis		-	**(8.47)**	[42]

cacna1sa	*CACNA1S*	PM	Ca^{2+} transmembrane transport	high voltage-gated Ca^{2+} channel activity	-	[42] **(0.6)**	
cacna1sb	*CACNA1S*	PM		high voltage-gated Ca^{2+} channel activity, protein binding	-	**(0.52)**	
cacna2d1a	***CACNA2D1***	PM	-	voltage-gated Ca^{2+} channel activity	-	**VERY LOW (20.71)**	**VERY LOW**
cacna2d2a	*CACNA2D2*	PM	-		-	**(19.95)**	
cacna2d2b	*CACNA2D2*	PM	-		-	**(102.25)**	
cacna2d3	***CACNA2D3***	PM	-		-	**VERY LOW (10.11)**	**VERY LOW**
cacna2d3a	*CACNA2D3*	PM	-	-	-	**(10.09)**	
cacna2d4a	*CACNA2D4*	PM	-	voltage-gated Ca^{2+} channel activity	-	**(133.13)**	
cacna2d4b	***CACNA2D4***	PM	-		-	**VERY LOW (15.91)**	**VERY LOW**
cacnb2a	*CACNB2*	PM	Ca^{2+} transmembrane transport, chemical synaptic transmission, embryonic heart tube morphogenesis, neuromuscular junction development, regulation of voltage-gated Ca^{2+} channel activity	high voltage-gated Ca^{2+} channel activity	abnormal cardiac ventricle sarcomere organization, disrupted cell-cell adhesion mediated by cadherin, malformed heart, decreased contractility of the heart, abnormal blood circulation, decreased cell proliferation involved in heart morphogenesis [47]	[48,49] **(51.20)**	
cacnb2b	*CACNB2*	PM	Ca^{2+} transmembrane transport, chemical synaptic transmission, neuromuscular junction development, regulation of voltage-gated Ca^{2+} channel activity		-	[48,49] **(40.02)**	
cacnb3a	*CACNB3*	PM			-	[48,49] **(0.56)**	
cacnb3b	*CACNB3*	PM			-	**(4.18)**	
cacnb4a	*CACNB4*	PM			initiation of epiboly arrested, abnormal division and dispersal of yolk syncytial nuclei, blastoderm retraction, and death [48]	[48,49] **(15.19)**	
cacnb4b	*CACNB4*	PM	Ca^{2+} transmembrane transport, chemical synaptic transmission, epiboly involved in gastrulation with mouth forming second, neuromuscular junction development, regulation of voltage-gated Ca^{2+} channel activity			[48,49] **(35.52)**	
cacng1a	*CACNG1*	PM	Ca^{2+} transmembrane transport	channel regulator activity, voltage-gated Ca^{2+} channel activity	-	**(0.3)**	
cacng1b	*CACNG1*	PM			-	**(5.07)**	
cacng2a	***CACNG2***	PM	AMPA selective glutamate receptor signaling pathway, Ca^{2+} transmembrane transport, neurotransmitter receptor internalization, neurotransmitter receptor transport,		edematous heart, abnormal startle response and thigmotaxis, decreased signaling via AMPA receptor in Mauthner neuron [50]	**[7,50]** **LOW (86.02)**	**VERY LOW**
cacng3b	***CACNG3***	PM			-	**LOW (57.02)**	**VERY LOW**

cacng4a	*CACNG1*	PM	postsynaptic endosome to lysosome, postsynaptic neurotransmitter receptor diffusion trapping, regulation of AMPA receptor activity, regulation of ion transmembrane transport, transmission of nerve impulse		-	**(2.66)**	
cacng4b	*CACNG4*	PM			-	**(15.89)**	
cacng5a	*CACNG1*	PM			-	**(4.87)**	
cacng5b	*CACNG5b*	PM			-	**(7.56)**	
cacng6a	*CACNG1*	PM	-	-	-	**(0.1)**	
cacng6b	*CACNG1*	PM	Ca^{2+} transmembrane transport	voltage-gated Ca^{2+} channel activity	-	**(0.78)**	
cacng7a	***CACNG7***	PM	AMPA selective glutamate receptor signaling pathway, Ca^{2+} transmembrane transport, neurotransmitter receptor internalization, neurotransmitter receptor transport, postsynaptic endosome to lysosome, postsynaptic neurotransmitter receptor diffusion trapping, regulation of AMPA receptor activity, regulation of ion transmembrane transport, transmission of nerve impulse		-	**LOW (45.71)**	**VERY LOW**
cacng7b	***CACNG7***	PM				**LOW (36.46)**	**VERY LOW**
cacng8a	***CACNG8***	PM				**LOW (76.87)**	**VERY LOW**
cacng8b	***CACNG8***	PM				**VERY LOW (88.64)**	**VERY LOW**
cadps2	*CADPS2*	N	dense core granule exocytosis, synaptic vesicle exocytosis	-	-	[6,7,51,52] **(38.19)**	
cadpsa	*CADPS*	C		-	-	[6] **(53.08)**	
cadpsb	*CADPS*	C		-	-	**(340.0)**	
calb1	*CALB1*	C	Ca^{2+} homeostasis, regulation of cytosolic Ca^{2+} concentration, regulation of long-term synaptic potentiation, regulation of presynaptic cytosolic Ca^{2+} concentration	Ca^{2+} binding	-	**(20.68)**	
calb2a	*CALB2*	C			-	[7,17,53] **(8.3)**	
calb2b	*CALB2*	C			-	[53-57] **(24.22)**	
calhm1	*CALHM1*	PM	cation transport	Ca^{2+} activated cation channel activity, voltage-gated ion channel activity	-	**(0.19)**	
calhm2	*CALHM2*	PM	-	-	-	**(0.25)**	
calhm3	*CALHM3*	PM	-	-	-	**(0.08)**	
calm1a	*CALM1*	PM/C/N/ER	midbrain-hindbrain boundary morphogenesis	-	abnormal morphology of midbrain hindbrain boundary cells [58]	[1,46,58] **(171.83)**	
calml4a	*CALML4*	N/C	Ca^{2+}-mediated signaling	Ca^{2+} binding	-	**(0.51)**	
calml4b	*CALML4*	N/C			-	**(36.32)**	

calr	*CALR*	ER	protein folding	Ca^{2+} binding, unfolded protein binding	-	[7] **(62.72)**
calr3a	*CALR3*	ER			decreased amount of neuroblasts [59]	[7] **(46.31)**
calr3b	*CALR3*	ER			-	[1,60] **(21.19)**
calua	*CALU*	ER/G	-	Ca^{2+} binding	-	[7] **(20.44)**
calub	*CALU*	ER/G	-		-	[7] **(6.63)**
canx	*CANX*	ER	posterior lateral line development, protein folding	Ca^{2+} binding, unfolded protein binding	decreased amount of neuromasts, abnormal development of posterior lateral line primordium [59]	[59] **(141.41)**
capn10	*CAPN10*	C	cellular response to insulin stimulus, proteolysis	Ca^{2+}-dependent cysteine-type endopeptidase activity, hydrolase activity	-	**(21.91)**
capn12	*CAPN12*	C	keratinocyte differentiation, proteolysis	Ca^{2+} binding, Ca^{2+}-dependent cysteine-type endopeptidase activity, hydrolase activity	increased size of keratinocytes, disrupted keratinocyte differentiation, edematous pericardium, increased mortality [61]	**(0.72)**
capn15	*CAPN15*	C	proteolysis		-	**(47.36)**
capn1a	*CAPN1*	C	brain development, branchiomotor neuron axon guidance, proteolysis		disorganized branchiomotor neuron, disrupted axon guidance of branchiomotor neuron, disrupted neuron migration, disrupted brain development, disorganized brain microtubule cytoskeleton, hydrocephalic brain, decreased size of eye, edematous pericardium, aplastic swim bladder [62]	[63] **(34.26)**
capn1b	*CAPN1*	C	negative regulation of apoptotic process, proteolysis		-	[63] **(0.45)**
capn2a	*CAPN2*	C	proteolysis, regulation of cytoskeleton organization		-	[63] **(2.64)**
capn2b	*CAPN2*	C			-	[7,63] **(1.73)**
capn3a	*CAPN3*	C	proteolysis, sarcomere organization		-	[7,64] **(0.63)**
capn5a	*CAPN5*	C	proteolysis		-	**(2.57)**
capn5b	*CAPN5*	C			-	**(3.49)**
capn7	*CAPN7*	C			-	**(14.8)**

capn9	*CAPN9*	C	digestion, proteolysis	Ca^{2+} binding, Ca^{2+}-dependent cysteine-type endopeptidase activity, hydrolase activity	-	[1,7] **(0.23)**	
casr	*CASR*	PM	G protein-coupled receptor signaling pathway, Ca^{2+} homeostasis, signal transduction, skeletal system development	G protein-coupled receptor activity	abnormal auditory behavior, increased area of Mauthner neuron cell body, *stc1l* expression decreased, disrupted Ca^{2+} homeostasis, disrupted myotome development and morphology, disrupted notochord development and morphology, cranial vault dorso-ventrally flattened [65-68]	[65,66,69] **(0.15)**	
cbarpb	*CBARP*	PM	negative regulation of voltage-gated Ca^{2+} channel activity	ion channel binding	-	[6] **(50.03)**	
ccar1	*CCAR1*	N	regulation of transcription, DNA-templated	nuclear receptor transcription coactivator activity	-	**(6.73)**	
ccar2	*CCAR2*	N/Mt	positive regulation of apoptotic process, regulation of transcription, DNA-templated, RNA splicing	-	-	**(19.58)**	
ccbe1	*CCBE1*	EM	angiogenesis, blood vessel morphogenesis, lymph vessel development, lymphangiogenesis, multicellular organism development, sprouting angiogenesis, vascular endothelial growth factor signaling pathway, venous blood vessel morphogenesis, venous endothelial cell migration involved in lymph vessel development	Ca^{2+} binding	-	[70] **(1.84)**	
cetn2	*CETN2*	N	centriole replication, microtubule cytoskeleton organization, nucleotide-excision repair		-	[71] **(25.83)**	
cetn3	*CETN3*	N	-		-	**(17.22)**	
cetn4	*CETN4*	N	cilium assembly, interkinetic nuclear migration, microtubule cytoskeleton organization		hydrocephalic brain, decreased size of eyes, edematous pericardium, post-vent region increased curvature, cystic pronephrons, disrupted cilium assembly, disorganized pronephric duct, regulation of cell cycle disrupted, interkinetic nuclear migration disrupted, presumptive neural retina decreased thickness and microtubule cytoskeleton disorganized [72,73]	[1,74,75] **(7.98)**	
cgref1	***CGREF1***	EM	cell adhesion, cell cycle arrest, negative regulation of cell proliferation		-	**MEDIUM (13.95)**	**MEDIUM**
cherp	***CHERP***	ER/C	RNA processing, cellular Ca^{2+} homeostasis	RNA binding	-	**VERY LOW (41.45)**	**VERY LOW**

chp1	***CHP1***	PM/ER/ N/C	-	Ca^{2+} binding	disrupted axonogenesis, hypoplastic cerebellum, decreased size of the optic tectum [76]	**[7]** **MEDIUM (11.68)**	**LOW**
chp2	***CHP2***	PM/N	-		-	**LOW (2.73)**	**LOW**
cisd2	***CISD2***	ER/Mt	autophagy, autophagy of mitochondrion, multicellular organism aging	iron-sulfur cluster binding, metal ion binding, protein homodimerization activity	-	**LOW (8.46)**	**LOW**
clgn	*CLGN*	ER	protein folding	Ca^{2+} binding, unfolded protein binding	-	[7] **(97.69)**	
clstn1	*CLSTN1*	PM	axon arborization, axonal transport, establishment or maintenance of microtubule cytoskeleton polarity, homophilic cell adhesion via plasma membrane adhesion molecules, positive regulation of synapse assembly, positive regulation of synaptic transmission, regulation of collateral sprouting, retrograde transport, endosome to plasma membrane	Ca^{2+} binding	decreased branchiness of Rohon-Beard neuron axon and trigeminal ganglion axon, decreased anterograde axonal transport in Rohon-Beard neuron, mislocalised Rohon-Beard neuron early endosome, decreased retrograde transport in Rohon-Beard neuron [77]	[7,77-79] **(535.32)**	
clstn2	*CLSTN2*	PM	homophilic cell adhesion via plasma membrane adhesion molecules, positive regulation of synapse assembly, positive regulation of synaptic transmission		-	[78] **(9.63)**	
cracr2aa	*CRACR2A*	PM	-	-	-	**(4.2)**	
cracr2ab	***CRACR2A***	PM	-	GTPase activity, Ca^{2+} binding	-	**VERY LOW (1.61)**	**VERY LOW**
cracr2b	*CRACR2B*	PM	-	Ca^{2+} binding	-	**(0.21)**	
creb1a	*CREB1*	N	convergent extension involved in axis elongation, midbrain-hindbrain boundary development, regulation of transcription, DNA-templated, somitogenesis	DNA-binding transcription factor activity	-	[80] **(22.59)**	
creb1b	*CREB1*	N	regulation of transcription, DNA-templated	DNA-binding transcription factor activity	-	**(35.10)**	
creb5a	*CREB5*	N		DNA-binding transcription factor activity, metal ion binding	-	**(7.03)**	
creb5b	*CREB5*	N			-	**(2.25)**	
crebl2	*CREBL2*	N	cell differentiation, positive regulation of fat cell differentiation, positive regulation of glucose import, positive regulation of lipid biosynthetic process, positive regulation of transcription, DNA-templated	DNA-binding transcription factor activity	-	**(95.06)**	

creb3l1	*CREB3L1*	N	endoplasmic reticulum unfolded protein response, positive regulation of transcription by RNA polymerase II, regulation of transcription, DNA-templated		-	**(5.3)**	
creb3l2	*CREB3L2*	ER	chondrocyte differentiation, endoplasmic reticulum unfolded protein response, multicellular organism development, positive regulation of transcription by RNA polymerase II, regulation of ER to Golgi vesicle-mediated transport, regulation of extracellular matrix constituent secretion, regulation of transcription, DNA-templated,	DNA-binding transcription factor activity, cAMP response element binding	Ceratohyal, Meckel's and palatoquadrate cartilages malformed, abnormal chondroblast spatial pattern, head and trunk decreased length, chondrocyte endoplasmic reticulum distended, dereased collagen biosynthesis, abnormal collagen fibril organization, decreased endoplasmic reticulum to Golgi vesicle-mediated transport and extracellular matrix constituent secretion, melanocyte melanosome immature and disorganized [81]	[81] **(9.7)**	
creb3l3a	*CREB3L3*	ER/N	endoplasmic reticulum unfolded protein response, positive regulation of transcription by RNA polymerase II, regulation of transcription, DNA-templated		-	[7] **(0.22)**	
creb3l3b	*CREB3L3*	ER/N			-	**(2.03)**	
creb3l4	*CREB3L4*	N	-	-	-	**(25.86)**	
<u>crebbpa</u>	***<u>CREBBP</u>***	N	hematopoietic progenitor cell differentiation, histone acetylation, regulation of transcription, DNA-templated	histone acetyltransferase activity, metal ion binding, transcription coactivator activity, zinc ion binding	-	**<u>[82,83]</u>** **<u>LOW</u> (18.01)**	**<u>VERY LOW</u>**
crebbpb	*CREBBP*	N	histone acetylation, regulation of transcription, DNA-templated		-	[82,83] **(7.65)**	
crebrf	*CREBRF*	N	regulation of transcription, DNA-templated, response to unfolded protein	DNA-binding transcription factor activity	-	**(19.56)**	
crebzf	*CREBZF*	N	regulation of DNA-binding transcription factor activity		-	**(61.58)**	
crtc1a	***CRTC1***	C	positive regulation of CREB transcription factor activity, positive regulation of transcription by RNA polymerase II, protein homotetramerization	cAMP response element binding protein binding	-	**LOW (13.02)**	**VERY LOW**
crtc1b	***CRTC1***	C			-	**LOW (6.22)**	**VERY LOW**
crtc2	*CRTC2*	C			-	**(14.85)**	
<u>crtc3</u>	***<u>CRTC3</u>***	C	positive regulation of CREB transcription factor activity, positive regulation of transcription by RNA polymerase II, protein homotetramerization, response to hypoxia		-	**<u>[84]</u>** **<u>LOW</u> (36.41)**	**<u>VERY LOW</u>**
efcab1	***EFCAB1***	C	-	Ca^{2+} binding	-	**VERY LOW (1.25)**	**VERY LOW**
efcab11	***EFCAB11***	C	-		-	**VERY LOW (2.26)**	**VERY LOW**

efcab2	*EFCAB2*	C	-	Ca^{2+} binding	-	**(0.75)**	
efcab6	*EFCAB6*	N	-		-	**(3.1)**	
efcab7	***EFCAB7***	PM	-		-	**VERY LOW (14.98)**	**VERY LOW**
efcc1	***EFCC1***	PM	-		-	**VERY LOW (2.58)**	**VERY LOW**
efemp1	*EFEMP1*	EM	-	-	-	**(24.62)**	
efemp2a	*EFEMP2*	EM	elastic fiber assembly	Ca^{2+} binding	-	[6,7,85] **(2.77)**	
efemp2b	*EFEMP2*	EM			-	[1,60,86] **(89.22)**	
efhb	*EFHB*	N/C	-		-	**(1.33)**	
efhc1	*EFHC1*	PM	-	-	-	[7] **(2.72)**	
efhc2	***EFHC2***	C	-	Ca^{2+} binding	-	**VERY LOW (2.34)**	**VERY LOW**
efhd1	***EFHD1***	Mt	-		-	**HIGH (67.44)**	**MEDIUM**
efhd2	***EFHD2***	PM	-		-	**LOW (73.39)**	**LOW**
egfl7	*EGFL7*	EM	anatomical structure development, vasculogenesis	Ca^{2+} binding, signaling receptor binding	hemorrhagic brain, abnormal intersegmental vessel morphology, *emilin3a* and *tp53* expression increased, disrupted blood circulation, abnormal axial vasculature, mislocalised and unlumenized dorsal aorta, disrupted establishment or maintenance of epithelial cell apical/basal polarity, deformed posterior cardinal vein, increased amount and mislocalised blood vessel endothelial cell bicellular tight junction, disrupted migration of blood vessel endothelial cells [87-89]	[90,91] **(3.37)**	
ehd1a	*EHD1*	PM/EM	cilium assembly	GTP binding, Ca^{2+} binding	-	[92] **(26.72)**	
ehd2a	*EHD2*	PM/C	actin cytoskeleton organization, endocytic recycling		-	**(0.29)**	
ehd2b	*EHD2*	PM/EM /C			-	[7] **(11.29)**	
ehd3	*EHD3*	PM/N	cilium assembly, endocytic recycling		decreased amount of photoreceptors in the outer segment layer, decreased amount and abnormal morphology of neuromast hair cell cilium [92]	[92] **(25.46)**	

ehd4	*EHD4*	N	endocytic recycling	GTP binding, Ca^{2+} binding	-	**(7.62)** [7]	
eps15	*EPS15*	PM/C	-	Ca^{2+} binding	-	**(47.61)**	
eps15l1a	*EPS15L1*	PM/N/C	-		-	**(10.52)**	
eps15l1b	*EPS15L1*	PM/N/C	T cell differentiation in thymus	-	thymus *lck* and *rag1* expression decreased, T cell differentiation in thymus disrupted [93]	**(1.64)** [93]	
faim2a	***FAIM2***	PM	-	-	-	**LOW (18.35)**	**VERY LOW**
faim2b	*FAIM2*	PM	-	-	-	**(0.09)**	
fbln1	*FBLN1*	EM	extracellular matrix organization, embryonic medial fin morphogenesis, heart development, mesenchymal cell migration, multicellular organism development, skin morphogenesis	Ca^{2+} binding, peptidase activator activity	-	**(19.35)** [17,85]	
fbln2	*FBLN2*	EM	extracellular matrix organization	Ca^{2+} binding	-	**(3.68)** [7,85]	
fbln5	*FBLN5*	EM	elastic fiber assembly		-	**(2.95)** [85]	
fbln7	*FBLN7*	EM	-		-	**(2.82)** [94]	
fbn2a	*FBN2*	EM	-	Ca^{2+} binding, extracellular matrix structural constituent	-	**(4.12)**	
fkbp1aa	*FKBP1A*	C	chaperone-mediated protein folding, protein peptidyl-prolyl isomerization	isomerase activity, peptidyl-prolyl cis-trans isomerase activity	-	**(54.61)**	
fkbp1b	*FKBP1B*	C			-	**(21.76)**	
fkbp2	*FKBP2*	EM/ER	-		-	**(14.38)**	
fkbp3	*FKBP3*	N	-		-	**(18.5)**	
fkbp4	*FKBP4*	C	chaperone-mediated protein folding, protein peptidyl-prolyl isomerization	FK506 binding, heat shock protein binding, isomerase activity, peptidyl-prolyl cis-trans isomerase activity	abnormal neurogenesis, curved and decreased length of body [95]	**(105.76)** [95]	
fkbp5	*FKBP5*	C			-	**(24.23)** [96,97]	
fkbp6	*FKBP6*	C	-	peptidyl-prolyl cis-trans isomerase activity	-	**(0.07)**	
fkbp7	*FKBP7*	EM	-	Ca^{2+} binding, isomerase activity, peptidyl-prolyl cis-trans isomerase activity	-	**(1.67)**	
fkbp8	*FKBP8*	PM	-	isomerase activity, peptidyl-prolyl cis-trans isomerase activity	-	**(26.7)**	

fkbp9	*FKBP9*	ER	-	Ca^{2+} binding, isomerase activity, peptidyl-prolyl cis-trans isomerase activity	-	**(6.49)**	
fkbp10a	*FKBP10*	C	chaperone-mediated protein folding	Ca^{2+} binding, FK506 binding, isomerase activity, peptidyl-prolyl cis-trans isomerase activity	-	**(7.41)**	
fkbp10b	*FKBP10*	ER	-	Ca^{2+} binding, isomerase activity, peptidyl-prolyl cis-trans isomerase activity	-	[7,98] **(6.38)**	
fkbp14	*FKBP14*	ER	-		-	[7] **(1.04)**	
fkbpl	*FKBPL*	ER	vasculature development	-	vasculature development disrupted [99]	**(10.5)**	
ghitm	***GHITM***	Mt	-	-	-	**VERY LOW (44.49)**	**VERY LOW**
gpat4	*GPAT4*	PM	-	transferase activity, transferring acyl groups	-	**(33.2)**	
gria1b	*GRIA1*	PM			-	[100,101] **(2.16)**	
gria2a	*GRIA2*	PM	anterior lateral line development, embryonic cranial skeleton morphogenesis, ion transport, nervous system development, neural crest cell development, posterior lateral line system development	AMPA glutamate receptor activity, ionotropic glutamate receptor activity, signaling receptor activity	increased size of fourth and third ventricles, decreased size of head, increased apoptosis, decreased amount of neuromasts in lateral line, abnormal motor neurons, abnormal cranial cartilage, abnnormal migration of neural crest [102]	[6,100,101] **(11.02)**	
gria2b	*GRIA2*	PM	ion transport		-	[100,101] **(360.24)**	
gria3a	*GRIA3*	PM			increased mortality [28]	[100,101] **(207.38)**	
gria3b	*GRIA3*	PM			-	[6,100,101] **(25.47)**	
gria4a	*GRIA4*	PM			-	[100,101,103] **(347.99)**	
gria4b	*GRIA4*	PM			-	[6,100,101,104] **(14.66)**	
grin1a	***GRIN1***	PM	G protein-coupled receptor signaling pathway, ion transport	G protein-coupled receptor activity, NMDA glutamate receptor activity, ion channel activity, ionotropic glutamate receptor activity	-	**[105,106]** **HIGH (459.99)**	**LOW**

grin1b	*GRIN1*	PM	ion transport	NMDA glutamate receptor activity, ion channel activity, ionotropic glutamate receptor activity	-	[105,106] **(250.15)**
grin2aa	*GRIN2A*	PM	excitatory postsynaptic potential, ion transport, long-term synaptic potentiation		-	**(103.48)**
grin2ab	*GRIN2A*	PM			-	**(27.37)**
grin2bb	*GRIN2B*	PM			-	**(188.21)**
grin2cb	*GRIN2C*	PM			-	[107] **(0.68)**
grin2da	*GRIN2D*	PM			-	[7] **(43.53)**
grin3a	*GRIN3A*	PM	ion transport	ion channel activity, ionotropic glutamate receptor activity	-	**(17.36)**
grin3ba	*GRIN3B*	PM			-	**(7.03)**
grin3bb	*GRIN3B*	PM			-	**(3.71)**
grinaa	*GRINA*	PM	-	-	-	**(83.79)**
grinab	*GRINA*	PM	negative regulation of apoptotic process, negative regulation of neuron death	-	increased occurance of programmed cell death, abnormal organisation of central nervous system, abnormal head morphology [108]	[7,108] **(5.24)**
hp	*HP*	EM	proteolysis	hemoglobin binding, serine-type endopeptidase activity		[109] **(0.06)**
hpca	*HPCA*	C	-	Ca^{2+} binding	-	[7,21] **(23.04)**
hpcal1	*HPCAL*	C	-		-	**(33.44)**
hpcal4	*HPCAL4*	C	-		-	**(127.7)**
hrc	*HRC*	PM	-	-	-	[7] **(0.12)**
itpr1a	*ITPR1*	ER	Ca^{2+} transmembrane transport	Ca^{2+} channel activity, inositol 1,4,5 trisphosphate binding, inositol 1,4,5-trisphosphate-sensitive Ca^{2+}-release channel activity	-	**(0.25)**
itpr1b	*ITPR1*	ER			-	[21] **(160.88)**
itpr2	*ITPR2*	ER	Ca^{2+} transmembrane transport, release of sequestered Ca^{2+} into cytosol		-	**(22.79)**
itpr3	*ITPR3*	ER	Ca^{2+} transmembrane transport, release of sequestered Ca^{2+} into cytosol, embryonic skeletal system development		malformed notochord [110]	[111] **(0.16)**
itsn1	*ITSN1*	PM/C	intracellular signal transduction, regulation of Rho protein signal transduction	Ca^{2+} binding, Rho-guanyl-nucleotide exchange factor activity	-	**(106.01)**
itsn2a	*ITSN2*	EM/C	endocytosis, intracellular signal transduction, regulation of Rho protein signal transduction		-	**(1.63)**
itsn2b	*ITSN2*	EM/C	endocytosis, regulation of Rho protein signal transduction		-	**(9.26)**

kcnip1a	*KCNIP1*	C/N	cilium movement	-	determination of left/right symmetry disrupted, otolith morphology, ventricular system hydrocephalic, whole organism anterior-posterior axis curved, Kupffer's vesicle motile cilium decreased amount [112]	[112] **(11.46)**	
kcnip1b	*KCNIP1*	PM	-	Ca^{2+} binding	abnormal heart contraction [113]	[7] **(10.81)**	
kcnip2	*KCNIP2*	PM	-		-	**(1.6)**	
kcnip3a	*KCNIP3*	PM	-		-	[7] **(54.48)**	
kcnip3b	*KCNIP3*	PM	endocrine pancreas development		affected pancreatic endocrine development [114]	[7] **(3.5)**	
kcnma1a	***KCNMA1***	PM	potassium ion transmembrane transport, regulation of ion transmembrane transport, response to auditory stimulus	large conductance Ca^{2+}-activated potassium channel activity, metal ion binding, voltage-gated potassium channel activity	abnormal response to auditory stimulus, abnormal swimming behavior [115]	**LOW (214.51)**	**VERY LOW**
kcnma1b	***KCNMA1***	PM	potassium ion transport, regulation of membrane potential, response to auditory stimulus	large conductance Ca^{2+}-activated potassium channel activity, outward rectifier potassium channel activity		**VERY LOW (6.52)**	**VERY LOW**
kcnmb2	*KCNMB2*	PM	detection of Ca^{2+}, potassium ion transport	Ca^{2+}-activated potassium channel activity, potassium channel regulator activity	-	**(0.13)**	
kcnn1a	***KCNN1***	PM	potassium ion transmembrane transport	small conductance Ca^{2+}-activated potassium channel activity, calmodulin binding	-	**VERY LOW (105.96)**	**VERY LOW**
kcnn1b	***KCNN1***	PM			-	**[116]** **VERY LOW (12.47)**	**VERY LOW**
kcnn3	*KCNN3*	PM			-	**(56.58)**	
kcnn4	*KCNN4*	PM			-	**(1.17)**	
kcnt1	***KCNT1***	PM	potassium ion transport, regulation of membrane potential	intracellular sodium activated potassium channel activity, outward rectifier potassium channel activity	-	**LOW (57.4)**	**VERY LOW**
kcnt2	*KCNT2*	PM			-	**(1.47)**	
lats1	*LATS1*	C	G1/S transition of mitotic cell cycle, convergent extension involved in gastrulation, hippo	ATP binding, protein serine/threonine kinase	disrupted convergent extension involved in gastrulation [117]	[117] **(37.58)**	

lats2	*LATS2*	C/N	signaling, intracellular signal transduction, peptidyl-serine phosphorylation, positive regulation of apoptotic process, regulation of organ growth	activity, transferase activity		**(1.36)**	[117]
letm1	***LETM1***	Mt	Ca^{2+} export from the mitochondrion, Ca^{2+} transport, mitochondrial Ca^{2+} homeostasis, protein hexamerization, protein homooligomerization	Ca^{2+} binding, Ca^{2+}:proton antiporter activity, ribosome binding	-	**VERY LOW (1.81)**	**VERY LOW**
letm2	***LETM2***	Mt	-	ribosome binding	-	**LOW (34.24)**	**LOW**
letmd1	*LETMD1*	Mt	-		-	**(0.42)**	
lpcat1	*LPCAT1*	G/ER	lipid metabolic process, phospholipid biosynthetic process	1-acylglycerophosphocholine O-acyltransferase activity, Ca^{2+} binding, transferase activity, transferring acyl groups	-	**(4.37)**	
lpcat2	*LPCAT2*	G/ER			-	**(3.46)**	
lpcat3	*LPCAT3*	PM	-	-	-	**(3.41)**	
lpcat4	*LPCAT4*	PM	-	Ca^{2+} binding, transferase activity, transferring acyl groups	-	**(1.36)**	[7]
ltbp1	*LTBP1*	EM/ER	-	Ca^{2+} binding	-	**(1.02)**	
ltbp3	*LTBP3*	EM	cardiac ventricle morphogenesis, outflow tract morphogenesis, regulation of smooth muscle tissue development	-	decreased amount of intestine smooth muscle cells, disrupted migration of lateral plate mesoderm tissue, abnormal cardiac ventricle, abnomal heart looping, abnormal outflow tract morphogenesis, hypoplastic presumptive bulbus arteriosus, decreased signaling via transforming growth factor beta receptor pathway, malformed pharyngeal vasculature [118,119]	**(11.93)**	[120-123]
mast1a	*MAST1*	PM	cytoskeleton organization, intracellular signal transduction, peptidyl-serine phosphorylation	ATP binding, kinase activity, magnesium ion bindin, protein serine/threonine kinase activity, transferase activity	-	**(51.16)**	
mast1b	*MAST1*	PM	protein phosphorylation		-	**(74.33)**	[60]
mast2	*MAST2*	PM/C	cytoskeleton organization, intracellular signal transduction, peptidyl-serine phosphorylation, regulation of interleukin-12 biosynthetic process		-	**(37.64)**	
mast3a	*MAST3*	PM/C	protein phosphorylation	ATP binding, magnesium ion binding, protein serine/threonine kinase activity	-	**(7.12)**	

mast3b	*MAST3*	PM/C	intracellular signal transduction, peptidyl-serine phosphorylation	ATP binding, kinase activity, magnesium ion bindin, protein serine/threonine kinase activity, transferase activity	-	**(87.53)**	
mast4	*MAST4*	C		protein serine/threonine kinase activity	-	**(226.83)**	
mastl	*MASTL*	C/N	G2/M transition of mitotic cell cycle, cell cycle, cell division, cellular response to DNA damage stimulus, intracellular signal transduction, negative regulation of phosphoprotein phosphatase activity, peptidyl-serine phosphorylation, thrombocyte differentiation	ATP binding, protein phosphatase 2A binding, protein serine/threonine kinase activity, transferase activity	decreased amount and disrupted differentiation of thrombocytes [124]	[7] **(2.46)**	
mcu	*MCU*	Mt	actin filament organization, Ca^{2+} import into the mitochondrion, Ca^{2+}-mediated signaling, chordate embryonic development, convergent extension involved in gastrulation, glucose homeostasis, mitochondrial Ca^{2+} homeostasis, positive regulation of insulin secretion, protein complex oligomerization, regulation of heart contraction	Ca^{2+} channel activity, identical protein binding, uniporter activity	abnormal actin polymerization and depolymerization, disrupted anterior/posterior axis specification, cell migration involved in gastrulation, disrupoted mitochondrial Ca^{2+} homeostasis, bent notochord [125]	[126] **(3.55)**	
micu1	***MICU1***	Mt	Ca^{2+} import, mitochondrial Ca^{2+} transmembrane transport, positive regulation of mitochondrial Ca^{2+} concentration	Ca^{2+} binding	-	**[127]** **VERY LOW (71.5)**	**VERY LOW**
micu2	***MICU2***	Mt	-		-	**VERY LOW (5.77)**	**VERY LOW**
micu3a	***MICU3***	Mt	-		-	**LOW (9.8)**	**LOW**
micu3b	*MICU3*	Mt	mitochondrial Ca^{2+} transmembrane transport			**(3.33)**	
myl10	*MYL10*	Mt/C	-		-	[60,128,129] **(0.18)**	
myl2b	*MYL2*	EM/C	-		heart morphogenesis disrupted [130]	[128,131] **(0.07)**	
myl6	*MYL6*	EM/C	-		-	**(8.05)**	
myl7	*MYL7*	C	atrial cardiac myofibril assembly, cardiac muscle cell proliferation, cardiac muscle tissue development, positive regulation of heart contraction, sarcomere organization, ventricular cardiac muscle cell development, ventricular cardiac myofibril assembly		decreased length of cardiac muscle sarcomeres, increased size of cardiac ventricle, edematous pericardium, decreased contractility and volume of cardiac ventricle, disrupted heart contraction, abnormal cardiac muscle cell shape [128,132]	[133-141] **(0.28)**	

myl9a	*MYL9*	C	-	Ca^{2+} binding	-	[7,142] **(10.06)**	
myl9b	*MYL9*	C	-		-	[143] **(17.89)**	
mylpfb	*MYLPF*	C	-		-	[6,7,128] **(1.42)**	
ncs1a	*NCS1*	PM	semicircular canal development		-	[7,111,144] **(32.11)**	
ncs1b	*NCS1*	PM	-		-	[144] **(26.82)**	
necab1	*NECAB1*	C	regulation of amyloid precursor protein biosynthetic process		-	[7,145] **(6.98)**	
necab2	*NECAB2*	PM			-	[7,145] **(42.54)**	
necab3	*NECAB3*	C	regulation of amyloid precursor protein biosynthetic process		-	**(43.03)**	
nell2a	*NELL2*	EM	-		-	**(86.76)**	
nell2b	*NELL2*	EM	-		-	**(223.22)**	
nox5	*NOX5*	PM	neutrophil migration, oxidation-reduction process, superoxide anion generation	Ca^{2+} binding, oxidoreductase activity, superoxide-generating NADPH oxidase activity	-	[146] **(0.99)**	
nucb1	*NUCB1*	EM/ER	-	Ca^{2+} binding	-	**(40.69)**	
nucb2b	*NUCB2*	EM/G	response to food		-	[7,147] **(8.66)**	
orai1a	*ORAI1*	PM	store-operated Ca^{2+} entry	store-operated Ca^{2+} channel activity	-	RT-PCR **(1.84)**	
orai2	*ORAI2*	PM	store-operated Ca^{2+} entry	store-operated Ca^{2+} channel activity	-	RT-PCR **(11.23)**	
ormdl1	*ORMDL1*	ER	cellular sphingolipid homeostasis, negative regulation of ceramide biosynthetic process, negative regulation of sphingolipid biosynthetic process	-	-	**(17.11)**	
ormdl2	*ORMDL2*	ER		-	-	**(9.43)**	
ormdl3	***ORMDL3***	ER		-	-	**LOW (0.95)**	**VERY LOW**
pdpk1a	*PDPK1*	PM/N/C	intracellular signal transduction, peptidyl-serine phosphorylation	ATP binding, protein serine/threonine kinase activity	-	**(9.71)**	
pdpk1b	*PDPK1*	PM/N/C			-	**(67.88)**	
pdzd8	***PDZD8***	ER	intracellular signal transduction, lipid transport, mitochondrial Ca^{2+} homeostasis, mitochondrion-endoplasmic reticulum membrane tethering	lipid binding, metal ion binding	-	**VERY LOW (4.6)**	**VERY LOW**

pkn1a	*PKN1*	N/C	intracellular signal transduction, peptidyl-serine phosphorylation	ATP binding, GTP-Rho binding, protein serine/threonine kinase activity, transferase activity	-	**(15.94)**
pkn1b	*PKN1*	N/C			-	**(51.43)**
pkn2	*PKN2*	N/C	embryonic viscerocranium morphogenesis, heart development, intracellular signal transduction, peptidyl-serine phosphorylation		edematous brain, decreased size of cranial cartilage, eyes and head, decreased amount of melanocytes, curved trunk, edematous pericardium, decrease body length [148]	**(4.24)**
pkn3	*PKN3*	N	intracellular signal transduction, peptidyl-serine phosphorylation		-	**(1.66)**
plcb3	*PLCB3*	N/C	cartilage morphogenesis, embryonic viscerocranium morphogenesis, inositol trisphosphate biosynthetic process, intracellular signal transduction, lipid catabolic process, negative regulation of Ca^{2+} import, negative regulation of vascular permeability, phosphatidylinositol-mediated signaling, release of sequestered Ca^{2+} into cytosol	Ca^{2+} binding, calmodulin binding, hydrolase activity, phosphatidylinositol phospholipase C activity, protein binding	aplastic branchiostegal ray 3, mislocalised cartilage elements, aplastic swim bladder, disrupted morphogenesis of embryonic viscerocranium, abnormal opercle shape, abnormal pharyngeal arch 3-7 skeleton and splanchnocranium, decreased amount of osteoblasts, abnormal ceratohyal-branchiostegal ray joint morphology, disrupted cartilage morphogenesis [149-152]	[7,152] **(9.09)**
plcg1	*PLCG1*	PM	angiogenesis, artery morphogenesis, fin regeneration, granulocyte differentiation, hematopoietic stem cell differentiation, inositol trisphosphate biosynthetic process, intracellular signal transduction, lipid catabolic process, phosphatidylinositol-mediated signaling, positive regulation of epithelial cell migration, regulation of angioblast cell migration involved in selective angioblast sprouting, regulation of heart contraction, release of sequestered Ca^{2+} into cytosol, vasculogenesis	hydrolase activity, phosphatidylinositol phospholipase C activity	disrupted angiogenesis, decreased amount of dorsal aorta angiogenic sprouts, edematous pericardium, disrupted granulocyte and myeloid leukocyte differentiation, sprouting angiogenesis arrested, disrupted vasculogenesis, arrested blood circulation, aplastic dorsal aorta and posterior cardinal vein, disrupted heart contraction, decreased eye and head size, curved trunk [153-159]	[157,160,161] **(60.46)**
plcg2	*PLCG2*	PM	inositol trisphosphate biosynthetic process, intracellular signal transduction, lipid catabolic process, phosphatidylinositol-mediated signaling, phospholipid catabolic process, release of sequestered Ca^{2+} into cytosol	hydrolase activity, phosphatidylinositol phospholipase C activity	-	**(2.5)**
plch1	*PLCH1*	PM/C	intracellular signal transduction, lipid catabolic process, phosphatidylinositol-mediated signaling	Ca^{2+} binding, Ca^{2+}-dependent phospholipase C activity, hydrolase activity, phosphoric diester hydrolase activity	-	**(35.14)**

plch2a	*PLCH2*	PM	inositol trisphosphate biosynthetic process, intracellular signal transduction, lipid catabolic process, phosphatidylinositol-mediated signaling, release of sequestered Ca^{2+} into cytosol	Ca^{2+} binding, hydrolase activity, phosphatidylinositol phospholipase C activity, phosphoric diester hydrolase activity	-	**(103.25)**	
plcl1	*PLCL1*	C	intracellular signal transduction, lipid catabolic process, regulation of synaptic transmission, GABAergic	GABA receptor binding, hydrolase activity, inositol 1,4,5 trisphosphate binding, phosphatidylinositol phospholipase C activity, phosphoric diester hydrolase activity	-	[162] **(122.90)**	
plcl2	*PLCL2*	PM			-	**(36.18)**	
ppef1	***PPEF1***	C	detection of stimulus involved in sensory perception	Ca^{2+} binding, hydrolase activity, iron ion binding, manganese ion binding, protein serine/threonine phosphatase activity	-	**VERY LOW (30.71)**	**VERY LOW**
ppef2b	*PPEF1*	C	-	-	-	**(10.67)**	
ppp1cab	*PPP1CA*	C/N			edematous pericardium, disrupted blood circulation, disrupted migration of blood vessel endothelial cells, disrupted branching morphogenesis of an epithelial tube, distended hindbrain, decreased functionality of intersegmental vessel, edematous yolk [163,164]	**(30.58)**	
ppp1cb	*PPP1CB*	C/N	convergent extension involved in axis elongation, liver development	hydrolase activity, metal ion binding, phosphoprotein phosphatase activity, protein serine/threonine phosphatase activity, protein heterodimerization activity	whole organism anterior-posterior axis curved and decreased length, disrupted liver development [165,166]	[166] **(151.15)**	
ppp1cbl	*PPP1CB*	C/N	circadian regulation of gene expression, convergent extension involved in axis elongation, regulation of circadian rhythm			**(55.66)**	
ppp1cc	*PPP1CC*	Mt/C/N	angiogenesis	-	blood circulation disrupted, blood vessel endothelial cell migration disrupted [163]	**(58.02)**	
prkcba	*PRKCB*	PM/EM /C/N	intracellular signal transduction, peptidyl-serine phosphorylation, protein phosphorylation	kinase activity, metal ion binding, protein kinase C activity, protein serine/threonine kinase activity, transferase activity, zinc ion binding	-	[7,167] **(30.74)**	

prkcbb	*PRKCB*	PM/C/N	B cell activation, B cell receptor signaling pathway, adaptive immune response, apoptotic process, blood coagulation, chromatin organization, embryonic hemopoiesis, intracellular signal transduction, peptidyl-serine phosphorylation, positive regulation of I-kappaB kinase/NF-kappaB signaling, regulation of transcription by RNA polymerase II	ATP binding, androgen receptor binding, chromatin binding, histone binding, histone kinase activity (H3-T6 specific), metal ion binding, nuclear receptor transcription coactivator activity, protein kinase C activity, protein serine/threonine kinase activity, transferase activity, zinc ion binding	decreased blood coagulation, fibrin clot formation, abnormal thrombocyte cellular adhesivity, disrupted embryonic hemopoiesis, decreased blood circulation, decreased eye size [168,169]	[167,170,171] **(221.76)**
prkcda	*PRKCD*	C/N	apoptotic process, cell cycle, intracellular signal transduction, peptidyl-serine phosphorylation	ATP binding, kinase activity, metal ion binding, protein serine/threonine kinase activity, transferase activity	-	[7] **(73.10)**
prkcea	*PRKCE*	PM/ER/C	intracellular signal transduction, peptidyl-serine phosphorylation		-	[170] **(141.31)**
prkceb	*PRKCE*	PM/ER/C	glomerular filtration, glomerular visceral epithelial cell development, intracellular signal transduction, peptidyl-serine phosphorylation		decreased life span, glomerular filtration disrupted, pericardium edematous, post-vent region curved, abnormal pronephric glomerulus podocytes foot morphology, decreased length, yolk syncytial layer edematous [172]	**(0.77)**
prkcg	*PRKCG*	PM/C	intracellular signal transduction, peptidyl-serine phosphorylation, receptor recycling, synapse maturation	ATP binding, kinase activity, metal ion binding, protein serine/threonine kinase activity, transferase activity, zinc ion binding	arrested hatching, disrupted mechanosensory behavior, disrupted synapse maturation [173]	[170,174] **(65.55)**
prkcha	*PRKCH*	PM/EM/C	intracellular signal transduction, peptidyl-serine phosphorylation	ATP binding, metal ion binding, protein kinase C activity, protein serine/threonine kinase activity, transferase activity	-	**(0.92)**
prkchb	*PRKCH*	PM/EM/C			-	**(0.57)**

prkci	*PRKCI*	EM/C/N	adherens junction maintenance, brain development, cell projection assembly, cell-cell junction maintenance, digestive tract mesoderm development, dorsal motor nucleus of vagus nerve development, embryonic heart tube development, establishment of mitotic spindle orientation, establishment or maintenance of polarity of embryonic epithelium, generation of neurons, heart development, intracellular signal transduction, mesodermal cell migration, multicellular organism development, neural tube formation, neuroblast proliferation, nuclear migration	ATP binding, metal ion binding, protein kinase C activity, protein serine/threonine kinase activity, transferase activity	abnormal peridermal cell actin-based cell projections, abnormal glomerular visceral epithelial cell migration, mislocalised pronephric podocytes, malformed pronephric proximal convoluted tubule, abnormal photoreceptor cell morphogenesis, abnormal spinal cord glia cells, increased size of central canal, abnormal ceratobranchial 5 bone, disrupted development of dorsal motor nucleus of vagus nerve, disrupted maintenance of epithelial cells apical/basal polarity, neuroepithelial cell cell-cell junction broken, abnormal cornea structure, disrupted brain development, cardiac ventricle and atrium dilated, disrupted heart morphogenesis, abnormal liver morphology, disrupted development of neuroblasts, abnormal pigmented epithelial cells shape, abnormal mitotic cell cycle, abnormal retina structure, decreased eye size, edematous pericardium, disrupted eye pigmentation, delayed melanocyte differentiation, disrupted blood circulation [153,175-185]	[176,178,180,186-190] **(73.75)**
prkcq	*PRKCQ*	PM/C	intracellular signal transduction, peptidyl-serine phosphorylation		-	[170] **(9.24)**
prkcz	*PRKCZ*	PM/EM /C	dermal bone morphogenesis, generation of neurons, intracellular signal transduction, nuclear migration, peptidyl-serine phosphorylation, photoreceptor cell outer segment organization, pronephric nephron tubule epithelial cell differentiation, regulation of cell migration involved in sprouting angiogenesis, retina morphogenesis in camera-type eye		caudal fin principal ray deformed, abnormal mouth position, abnormal scale spatial pattern, dorsal longitudinal anastomotic vessel aplastic, intersegmental vessel malformed [110,191]	[170,176,192-195] **(20.34)**

psen1	*PSEN1*	G/ER/PM	Notch signaling pathway, amyloid-beta formation, brain development, canonical Wnt signaling pathway, lymphangiogenesis, melanocyte differentiation, membrane protein ectodomain proteolysis, optomotor response, positive regulation of catalytic activity, proteolysis, regulation of endopeptidase activity, response to hypoxia, somite development, swimming behavior	aspartic-type endopeptidase activity, hydrolase activity	abnormal blood accumulation pharyngeal arch 3-7, brain edematous, disrupted brain development, fourth and tectal ventricle increased size, hydrocephalic hindbrain and midbrain, abnormal eye morphology, edematous pericardium, abnormal post-vent morphology, abnormal optomotor response, decreased expression of *baxa* and increase expression of *neurog1*, decreased swimming distance, canonical Wnt and Notch signaling pathway disrupted, lymphangiogenesis disrupted, thoracic duct absent, decreased amount of melanocytes, neural crest cell development disrupted [196-199]	[17,200] **(28.14)**
psen2	*PSEN2*	G/ER/PM	Notch receptor processing, Notch signaling pathway, amyloid-beta formation, brain development, Ca^{2+} transport, melanocyte differentiation, membrane protein ectodomain proteolysis, neurogenesis, protein processing, proteolysis, somite development		increased size of fourth and tectal ventricles, hydrocephalic hindbrain and midbrain, decreased amount of melanocytes, decreased amount of neural crest cells [201]	[202-205] **(14.82)**
pvalb1	-	C	regulation of cytosolic Ca^{2+} concentration	Ca^{2+} binding	-	[6] **(1.09)**
pvalb4	-	C			-	**(0.24)**
pvalb5	-	C/N	-		-	[7,206] **(0.17)**
pvalb7	*PVALB*	C			-	[7,21,56,207,208] **(7.39)**
pvalb8	-	C			-	[7,209,210] **(2.58)**
rcn1	*RCN1*	ER			-	[7] **(13.74)**
reps1	*REPS1*	PM/C	receptor-mediated endocytosis		-	**(38.37)**
reps2	*REPS2*	PM/C	-		-	[211] **(10.36)**

ryr1a	*RYR1*	ER	Ca^{2+} transmembrane transport, cellular Ca^{2+} homeostasis, release of sequestered Ca^{2+} into cytosol	Ca^{2+} channel activity, Ca^{2+} binding, Ca^{2+}-induced Ca^{2+} release activity, protein binding, ryanodine-sensitive Ca^{2+}release channel activity	decreased amount of slow muscle cells [212]	[129,213,214] **(1.89)**
ryr1b	*RYR1*	ER	Ca^{2+} transmembrane transport, cellular Ca^{2+} homeostasis, cellular response to oxidative stress, fast-twitch skeletal muscle fiber contraction, larval locomotory behavior, release of sequestered Ca^{2+} into cytosol		increased protein oxidation, decreased larval locomotory behavior, swollen skeletal muscle longitudinal sarcoplasmic reticulum, disorganized skeletal muscle myofibril, abnormal fast-twitch skeletal muscle fiber contraction, decreased intensity of muscle contraction, decreased activity of voltage-gated Ca^{2+} channel [215-218]	[214,217] **(165.49)**
ryr2a	*RYR2*	ER	Ca^{2+} transport	Ca^{2+} binding	-	[214] **(308.43)**
ryr2b	*RYR2*	ER	Ca^{2+} transmembrane transport, cellular Ca^{2+} homeostasis, release of sequestered Ca^{2+} into cytosol	Ca^{2+} channel activity, Ca^{2+} binding, Ca^{2+}-induced Ca^{2+} release, ryanodine-sensitive Ca^{2+}-release channel activity	-	[204,214] **(0.05)**
ryr3	*RYR3*	ER		Ca^{2+} channel activity, Ca^{2+} binding, protein binding, Ca^{2+}-induced Ca^{2+} release, ryanodine-sensitive Ca^{2+}-release channel activity	decreased amount of slow muscle cells, disrupted determination of left/right asymmetry in lateral mesoderm, decreased activity of calmodulin-dependent protein kinase [212,219]	[6,214,220] **(71.83)**
s100a1	*S100A1*	C	regulation of heart contraction	Ca^{2+} binding	-	[221,222] **(0.51)**
s100a10b	*S100A10*	C	regulation of cell proliferation	Ca^{2+} binding, Ca^{2+}-dependent protein binding	-	[7,222] **(11.33)**
s100a11	*S100A11*	C	-	Ca^{2+} binding, transition metal ion binding	-	[222] **(0.6)**
s100b	*S100B*	C	-	Ca^{2+} binding, zinc ion binding	-	[17,222] **(86.99)**
s100s	-	C	-	Ca^{2+} binding, transition metal ion binding	-	[222] **(1.11)**
s100t	-	C	-		-	[7,222] **(0.06)**
s100u	-	C	-	Ca^{2+} binding, copper ion binding	-	**(4.59)**
s100v2	-	C	-		-	[17] **(8.58)**
s100w	*S100W*	C	regulation of cell proliferation	Ca^{2+} binding, Ca^{2+}-dependent protein binding	-	**(0.1)**
s100z	*S100Z*	C	-	Ca^{2+} binding	-	[7,222] **(4.09)**

saraf	***SARAF***	ER	Ca^{2+} transport, regulation of store-operated Ca^{2+} entry	-	-	**VERY LOW (12.61)**	**MEDIUM**
scgn	***SCGN***	C	brain development, regulation of long-term, synaptic potentiation, regulation of presynaptic cytosolic Ca^{2+} concentration	Ca^{2+} binding	brain decreased size and edematous, pericardium edematous [223]	**VERY LOW (0.05)**	**VERY LOW**
sgca	*SGCA*	PM	-		-	[7] **(1.15)**	
sgcb	*SGCB*	PM	-	-	-	[224] **(68.52)**	
sgcd	*SGCD*	PM	cardiac muscle tissue development, determination of left/right symmetry, heart contraction, somatic muscle development, swim bladder inflation	-	disrupted blood circulation, decreased heart concractility, edematous pericardium [225]	[204,225-227] **(11.57)**	
sgce	*SGCE*	PM	-	Ca^{2+} binding	-	[7] **(90.98)**	
sgcg	*SGCG*	PM	cardiac muscle tissue development, heart contraction	-	-	[7,226,228] **(0.27)**	
sgcz	*SGCZ*	PM	-	-	-	**(5.62)**	
sgk1	*SGK1*	C/ER/N	apoptotic process, inflammatory response, intracellular signal transduction, peptidyl-serine phosphorylation	ATP binding, potassium channel regulator activity, protein serine/threonine kinase activity, transferase activity	-	[1,7,229,230] **(22.38)**	
sgk2a	*SGK2*	N/C			-	[211] **(1.24)**	
sgk2b	*SGK2*	N/C			-	**(1.5)**	
sgk3	*SGK3*	C			-	**(17.12)**	
sigmar1	***SIGMAR1***	ER/PM/N/C	lipid transport, response to wounding	-	-	**LOW (17.2)**	**LOW**
slc24a1	***SLC24A1***	PM	Ca^{2+} transmembrane transport, cellular Ca^{2+} homeostasis, long-term synaptic depression, long-term synaptic potentiation	Ca^{2+} channel activity, Ca^{2+}, potassium:sodium antiporter activity	-	**VERY LOW (0.75)**	**VERY LOW**
slc24a2	*SLC24A2*	PM	Ca^{2+} transmembrane transport, cellular Ca^{2+} homeostasis, long-term synaptic depression, long-term synaptic potentiation, visual perception		-	**(5.03)**	
slc24a3	***SLC24A3***	PM	Ca^{2+} transmembrane transport, cellular Ca^{2+} homeostasis		-	**MEDIUM (61.86)**	**MEDIUM**
slc24a4a	***SLC24A4***	PM			-	**[7]** **LOW (16.8)**	**LOW**
slc24a4b	***SLC24A4***	PM			-	**LOW (13.48)**	**LOW**

slc24a5	*SLC24A5*	PM	developmental pigmentation, ion transport, melanocyte differentiation, melanocyte migration, regulation of melanin biosynthetic process	Ca^{2+} channel activity, Ca^{2+}, potassium:sodium antiporter activity	decreased pigmentation [231]	[232] **(0.42)**	
slc25a12	SLC25A12	Mt	L-glutamate transmembrane transport, aspartate transmembrane transport, malate-aspartate shuttle	L-aspartate transmembrane transporter activity, L-glutamate transmembrane transporter activity, Ca^{2+} binding	-	[1] **(66.76)**	
slc25a19	*SLC25A19*	PM	thiamine pyrophosphate transmembrane transport	thiamine transmembrane transporter activity	-	**(4.91)**	
slc25a23a	*SLC25A23*	PM	transmembrane transport	ATP transmembrane transporter activity, Ca^{2+} binding	-	**(33.0)**	
slc25a23b	*SLC25A23*	PM			-	**(16.76)**	
slc25a24	*SLC25A24*	Mt	cellular response to Ca^{2+}, cellular response to oxidative stress, mitochondrial transport, regulation of cell death		-	[7] **(4.97)**	
slc25a24l	*SLC25A25*	Mt	transmembrane transport		eye decreased size, gut and liver hypoplastic, head circular and decreased size, mandibular arch skeleton decreased size, edematous pericardium [153]	**(3.44)**	
slc25a25a	SLC25A25	Mt			-	[111] **(3.99)**	
slc25a25b	SLC25A25	Mt		Ca^{2+} binding, transmembrane transporter activity	-	**(4.17)**	
slc25a28	***SLC25A28***	Mt	ion transport, iron import into the mitochondrion, iron ion homeostasis	iron ion transmembrane transporter activity	-	**HIGH (0.05)**	**HIGH**
slc25a37	*SLC25A37*	Mt	embryonic hemopoiesis, erythrocyte development, iron import into the mitochondrion, iron ion homeostasis		decreased amount of erythroid lineage cells, intestinal epithelium hyperplastic [233-235]	[235] **(1.19)**	
slc25a39	*SLC25A39*	Mt	heme biosynthetic process, manganese ion transport, transmembrane transport	metallochaperone activity, transmembrane transporter activity	disrupted heme biosynthetic process, decreased amount of nucleate erythrocytes, abnormal nucleate erythrocyte hemoglobin complex composition [236]	[236] **(20.97)**	
slc25a42	*SLC25A42*	Mt	mitochondrial membrane organization, transmembrane transport	ADP transmembrane transporter activity, AMP transmembrane transporter activity, ATP transmembrane transporter activity, coenzyme A transmembrane transporter activity	disrupted hatching, curved trunk, swollen skeletal muscle mitochondrial membrane [237]	**(7.41)**	

slc25a43	*SLC25A43*	Mt	transmembrane transport	transmembrane transporter activity	-	**(7.07)**	
slc8a1a	*SLC8A1*	PM	Ca^{2+} transport, cell communication, cellular Ca^{2+} homeostasis, regulation of heart contraction, sarcomere organization		abnormal atrium morphology, abnormal cardiac muscle cells shape and length, cardiac muscle cell myofibrils disorganized, heart trim63a expression increased, heart contraction asynchronous and decreased rate [127,238]	[20,204,239] **(14.88)**	
slc8a1b	*SLC8A1*	PM		Ca^{2+}:sodium antiporter activity	-	[25,240] **(6.81)**	
<u>slc8a2a</u>	***<u>SLC8A2</u>***	PM	Ca^{2+} transport, cell communication, transmembrane transport		-	**<u>[25]</u>** **<u>VERY LOW</u>**	**<u>VERY LOW</u>**
slc8a2b	*SLC8A2*	PM			-	[25] **(50.67)**	
slc8a3	*SLC8A3*	PM			-	[25] **(18.6)**	
slc8a4a	-	PM	Ca^{2+} transport, cell communication, determination of left/right symmetry, fluid transport, heart contraction, heart looping, regulation of cytosolic Ca^{2+} concentration, skeletal muscle tissue development, transmembrane transport	Ca^{2+}:sodium antiporter activity	decrease rate of heart contraction, edematous pericardium, abnormal mechanosensory behavior, disrupted determination of left/right symmetry, disrupted development of digestive truck, heart tube and liver, abnormal Kupffer's vesicle cilium movement [241,242]	[25,243] **(73.66)**	
slc8a4b	-	PM	Ca^{2+} transport, cell communication, transmembrane transport		-	[25] **(19.66)**	
slc8b1	*SLC8B1*	PM	transmembrane transport	-	-	**(2.1)**	
smdt1b	***SMDT1***	Mt	Ca^{2+} import into the mitochondrion, mitochondrial Ca^{2+} homeostasis	-	-	**MEDIUM (13.29)**	**MEDIUM**
smoc1	*SMOC1*	EM	eye development, optic cup morphogenesis involved in camera-type eye development	Ca^{2+} binding, extracellular matrix binding	decreased eye size, optic fissure open, immature retina, abnormal forebrain morphology [244]	[244] **(0.9)**	
smoc2	*SMOC2*	EM	embryonic viscerocranium morphogenesis, myeloid cell development, odontogenesis, regulation of BMP signaling pathway	Ca^{2+} binding	decreased signaling via BMP pathway, abnormal blood circulation, curved caudal fin, abnormal somites shape, abnormal hemopoiesis process and myeloid cells development, abnormal cartilage morphology, absent eyes, decreased head size, disrupted bone mineralization [245-247]	[1,245-247] **(14.05)**	
srl	*SRL*	ER	-	GTP binding	-	[7,18,248] **(0.87)**	
ssr1	*SSR1*	ER	cellular response to estrogen stimulus	-	-	[1,249,250] **(40.77)**	

stim1a	*STIM1*	ER	activation of store-operated Ca^{2+} channel activity, cellular Ca^{2+} homeostasis, regulation of Ca^{2+} transport, store-operated Ca^{2+} entry	Ca^{2+} channel regulator activity, Ca^{2+} binding	-	**(106.68)**
stim1b	*STIM1*	ER			-	**(1.85)**
stim2a	*STIM2*	ER	-	Ca^{2+} channel regulator activity	-	**(0.14)**
stim2b	*STIM2*	ER	activation of store-operated Ca^{2+} channel activity, cellular Ca^{2+} homeostasis, regulation of Ca^{2+} transport, store-operated Ca^{2+} entry	Ca^{2+} channel regulator activity, Ca^{2+} binding	-	**(2.68)**
stk32a	*STK32*	N/C	intracellular signal transduction, peptidyl-serine phosphorylation	ATP binding, protein serine/threonine kinase activity, transferase activity	-	**(5.4)**
stk38a	*STK38*	N/C			-	**(6.05)**
stk38b	*STK38*	N/C			-	**(1.23)**
stk38l	*STK38L*	C			-	[7] **(35.95)**
tdgf1	*TDGF1*	EM	BMP signaling pathway, adrenal gland development, anterior/posterior pattern specification, brain morphogenesis, cardioblast migration to the midline involved in heart field formation, central nervous system development, convergent extension involved in gastrulation, convergent extension involved in nephron morphogenesis, determination of left/right symmetry, digestive tract development, dorsal/ventral pattern formation, ectoderm and endoderm development, floor plate formation, forebrain development, germ cell migration, heart development, hypoblast development, hypothalamus development, induction of positive chemotaxis	activin receptor binding, nodal binding	*eaf2* expression decreased, presumptive endoderm and hypoblast *sox32* expression absent, margin *lft1* expression absent, *tbxta* and *aldh1a2* expression decreased, germ ring and margin dorsal region *fscn1a* expression absent, axial hypoblast and shield *gsc* expression and forerunner cell group *rbp7a* expression absent, midbrain posterior region *rbp7a* expression increased, neuroectoderm anterior region *sox2* expression decreased, neuroectoderm posterior region *hoxb1b* expression decreased, abnormal mesendoderm development, decreased nodal signaling pathway, epiboly decreased duration, eyes fused, abnormal mesoderm and prechordal plate morphology, whole organism altered number of cells, abnormal neural tube closure and pineal complex shape, abnormal neural tube morphology, abnormal neural plate morphogenesis, germ ring poorly differentiated, pronephric glomerulus morphogenesis abnormal, abnormal cardioblast migration, dorsal/ventral pattern formation disrupted, cell-cell adhesion involved in mesendodermal cell migration disrupted, forebrain development disrupted, endoderm formation disrupted, abnormal endocrine pancreas development [251-266]	[1,267-269] **(0.05)**

tenm1	***TENM1***	PM/N	heterophilic cell-cell adhesion via plasma membrane cell adhesion molecules, neuron development, signal transduction	cell adhesion molecule binding, protein heterodimerization activity, protein homodimerization activity	-	**MEDIUM (191.97)**	**MEDIUM**
						**[1,60,271,272]**	
tenm3	***TENM3***	PM/N	cell adhesion, cell differentiation, dendrite guidance, heterophilic cell-cell adhesion via plasma membrane cell adhesion molecules, homophilic cell adhesion via plasma membrane adhesion molecules, neuron development, positive regulation of neuron projection development, retinal ganglion cell axon guidance, signal transduction, visual perception		mislocalised retinal ganglion cell dendrites, abnormal pigment accumulation [270]	**LOW (217.92)**	**LOW**
tenm4	*TENM4*	C/PM/N	axon guidance, cell differentiation, central nervous system myelin formation, cell-cell adhesion via plasma membrane cell adhesion molecules, multicellular organism development, neuron development, positive regulation of gastrulation, positive regulation of neuron projection development, positive regulation of oligodendrocyte differentiation, signal transduction	catalytic activity, cell adhesion molecule binding, protein heterodimerization activity, protein homodimerization activity	axon guidance disrupted, abnormal motor neuron axon branchiness, peripheral nervous system axonogenesis involved in innervation disrupted [273]	**(95.52)**	[271]
thbd	*THBD*	PM	-	Ca^{2+} binding, transmembrane signaling receptor activity	-	**(1.85)**	
tmbim1a	*TMBIM1*	PM	-	-	-	**(29.26)**	
tmbim4	***TMBIM4***	G	-	-	-	**HIGH (53.1)**	**HIGH**
tmx1	***TMX1***	ER	cell redox homeostasis	-	-	**LOW (12.95)**	**LOW**
tnnc1a	*TNNC1*	C	cardiac muscle contraction, cardiac ventricle development, response to activity, skeletal muscle contraction	actin filament binding, Ca^{2+} binding, Ca^{2+}-dependent protein binding	abnormal atrium size, abnormal blood circulation, disrupted cardiac ventricle development , edematous pericardium, disorganised cardiac muscle cells myofibrils and ventricular myocardium sarcomeres, disrupted cardiac conduction and heart contraction [132,274]	**(0.16)**	[274-278]
tnnc1b	*TNNC1*	C	cardiac muscle contraction, regulation of muscle contraction, skeletal muscle contraction		-	**(0.17)**	[7,129,276,278-281]

tpcn1	***TPCN1***	PM	ion transport, transmembrane transport	NAADP-sensitive Ca^{2+} release channel activity, voltage-gated Ca^{2+} channel activity	-	**VERY LOW (14.87)**	**VERY LOW**
tpcn2	*TPCN2*	PM	Ca^{2+} transmembrane transport, Ca^{2+}-mediated signaling, regulation of ion transmembrane transport, regulation of myotome development, skeletal myofibril assembly, smooth muscle contraction		asynchronous CaP motoneuron release of sequestered Ca^{2+} into cytosol, blood cell accumulation in the heart, eye, head and trunk decreased pigmentation, myotome decreased width, slow muscle cell skeletal muscle myofibril disorganized [282,283]	[284] **(0.54)**	
trpa1a	*TRPA1*	PM	detection of chemical stimulus, detection of mechanical stimulus involved in sensory perception, ion transmembrane transport	Ca^{2+} release channel activity, ligand-gated ion channel activity, mechanosensitive ion channel activity	-	[285] **(14.89)**	
trpa1b	*TRPA1*	PM			abnormal chemosensory behavior [286]	[285,287] **(0.69)**	
trpc1	*TRPC1*	PM	angiogenesis, Ca^{2+} transmembrane transport, filopodium assembly, manganese ion transport, regulation of cytosolic Ca^{2+} concentration, vasculogenesis	Ca^{2+} channel activity, inositol 1,4,5 trisphosphate binding, store-operated Ca^{2+} channel activity	eye decreased size, intersegmental vein decreased size, post-vent region increased curvature, abnormal vasculogenesis paedomorphic growth, abnormal filopodium assembly paedomorphic growth [288]	[111,289-291] **(57.22)**	
trpc2a	*TRPC2*	PM	Ca^{2+} transmembrane transport	Ca^{2+} channel activity	-	[290,292] **(0.39)**	
trpc2b	*TRPC2*	PM	Ca^{2+} transmembrane transport, manganese ion transport, regulation of cytosolic Ca^{2+} concentration, single fertilization	Ca^{2+} channel activity, inositol 1,4,5 trisphosphate binding, store-operated Ca^{2+} channel activity	-	[290,293,294] **(0.25)**	
trpc3	*TRPC3*	PM	Ca^{2+} transmembrane transport, manganese ion transport, regulation of cytosolic Ca^{2+} concentration, single fertilization	Ca^{2+} channel activity, inositol 1,4,5 trisphosphate binding, store-operated Ca^{2+} channel activity	-	[290] **(18.57)**	
trpc4a	*TRPC4*	PM	Ca^{2+} transmembrane transport	Ca^{2+} channel activity	-	[290] **(20.97)**	
trpc4b	*TRPC4*	PM	Ca^{2+} transmembrane transport, manganese ion transport, regulation of cytosolic Ca^{2+} concentration, protein homotetramerization	Ca^{2+} channel activity, inositol 1,4,5 trisphosphate binding, store-operated Ca^{2+} channel activity	-	[290] **(11.42)**	
trpc5a	*TRPC5*	PM	Ca^{2+} transmembrane transport, manganese ion transport, regulation of cytosolic Ca^{2+} concentration		-	[111,290] **(3.07)**	
trpc6a	***TRPC6***	PM	Ca^{2+} transmembrane transport, manganese ion transport, regulation of cytosolic Ca^{2+} concentration, single fertilization		-	**VERY LOW (0.91)**	**VERY LOW**
trpc6b	*TRPC6*	PM				[290] **(13.28)**	

trpc7b	*TRPC7*	PM	Ca^{2+} transmembrane transport	Ca^{2+} channel activity	-	[290]<br>**(4.29)**	
trpm1b	*TRPM1*	PM	G protein-coupled glutamate receptor signaling pathway, cellular response to light stimulus, protein tetramerization	cation channel activity	-	[295]<br>**(0.4)**	
trpm2	*TRPM2*	PM	cation transport, ion transmembrane transport, response to oxidative stress	ADP-ribose diphosphatase activity, cation channel activity, hydrolase activity	-	[295]<br>**(2.85)**	
trpm3	***TRPM3***	PM	cation transport, detection of temperature stimulus, ion transmembrane transport, protein tetramerization	Ca^{2+} activated cation channel activity	-	**[295]**<br>**VERY LOW (102.34)**	**VERY LOW**
trpm4a	***TRPM4***	PM/ER/G	cardiac conduction, cation transport, ion transmembrane transport, regulation of T cell cytokine production, regulation of membrane potential, vasoconstriction	Ca^{2+} activated cation channel activity	-	**VERY LOW (1.06)**	**VERY LOW**
trpm4b.1	*TRPM4*	PM			-	**(0.12)**	
trpm4b.2	*TRPM4*	PM			-	**(0.1)**	
trpm5	*TRPM5*	PM	Ca^{2+} transmembrane transport, sensory perception of taste		-	[289,295,296]<br>**(0.49)**	
trpm6	*TRPM6*	PM	cation transport, protein phosphorylation, protein tetramerization	ATP binding, cation channel activity, protein serine/threonine kinase activity	-	[297]<br>**(1.49)**	
trpm7	***TRPM7***	PM	cation transport, cellular magnesium ion homeostasis, developmental pigmentation, divalent inorganic cation homeostasis, exocrine pancreas development, ion transmembrane transport, magnesium ion homeostasis, melanin metabolic process, melanocyte differentiation, ossification, protein phosphorylation, protein tetramerization, regulation of epithelial cell proliferation, regulation of heart rate, regulation of mitotic cell cycle, regulation of neurotransmitter secretion, renal system process, sensory perception of taste, skeletal system development		disrupted regulation of melanocytes membrane potential, abnormal swimming behawior, decreased amount of dopaminergic neurons, increased amount of pretectum serotonergic neurons, decreased heart contraction rate, decreased amount of melanocytes, arrested thigmotaxis, decreased pigmentation, decreased size of acinar cells, hypoplastic exocrine pancreas, disrupted mitotic cell cycle, abnormal calicum and magnesium ions homeostasis, obstracted pronephric duct, decreased functionallity of kidneys [298-303]	**[1,295,301,304,305]**<br>**VERY LOW (84.68)**	**VERY LOW**
trpn1	*TRPN1*	PM	Ca^{2+} transmembrane transport, detection of mechanical stimulus involved in sensory perception of sound	Ca^{2+} channel activity, mechanosensitive ion channel activity	-	[289]<br>**(2.25)**	

trpv1	*TRPV1*	PM	ion transport, response to heat, thermosensory behavior	Ca^{2+} channel activity	-	[287,289,306] **(1.55)**	
trpv4	*TRPV4*	PM	atrioventricular valve development, cellular response to oscillatory fluid shear stress, ion transport, response to osmotic stress		atrioventricular canal endocardium EGFP expression decreased, abnormal atrioventricular canal and valve morphogenesis, atrioventricular canal endocardium endothelial cell decreased amount, hydrocephalic brain [307,308]	[289,309-311] **(1.24)**	
trpv6	*TRPV6*	PM	Ca^{2+} homeostasis, Ca^{2+} import, hypotonic salinity response, positive regulation of transcription, DNA-templated, response to Ca^{2+}	Ca^{2+} channel activity	bone mineralization and ossification decreased, abnormal postcranial axial skeleton cartilaginous [312]	[69,289,313,314] **(1.22)**	
ttc9b	*TTC9B*	Mt/C	-	-	-	**(17.38)**	
ttc9c	*TTC9C*	N	cilium assembly, determination of heart left/right asymmetry, otolith morphogenesis	-	curved trunk, hydrocephalic brain, disrupted determination of heart left/right asymmetry disrupted, abnormal otolith morphology, ventricular system dilated, abnormal cilium movement, decreased amount and length of Kupffer's vesicle cilium, pronephric duct motile cilium disorganized [204]	**(7.71)**	
tusc2a	***TUSC2***	Mt	-	-	-	**MEDIUM (11.61)**	**LOW**
tusc2b	***TUSC2***	Mt	-	-	-	**VERY LOW (22.61)**	**LOW**
vdac1	*VDAC1*	Mt	anion transmembrane transport	voltage-gated anion channel activity	-	[315] **(96.08)**	
vdac2	*VDAC2*	Mt	anion transmembrane transport, Ca^{2+} import into the mitochondrion, fin regeneration, regulation of heart contraction,		-	[17,127] **(100.88)**	
vdac3	*VDAC3*	Mt	anion transmembrane transport, fin regeneration, regulation of cilium assembly		-	[7] **(86.1)**	
vwde	*VWDE*	EM	-	signaling receptor binding	-	**(0.16)**	
wfs1a	*WFS1*	ER	Ca^{2+} homeostasis	-	-	[7] **(1.91)**	
wif1	*WIF1*	EM	negative regulation of canonical Wnt signaling pathway, swim bladder development	signaling receptor binding	increased signaling via canonical Wnt pathway, abnormal anterior swim bladder, immature somites, decreased trunk length, abnormal pancreas morphology, disrupted smooth muscles development [316]	[6,316-321] **(3.09)**	

1. Thisse, B.; Pflumio, S.; Fürthauer, M.; Loppin, B.; Heyer, V.; Degrave, A.; Woehl, R.; Lux, A.; Steffan, T.; Charbonnier, X.Q., et al. Expression of the zebrafish genome during embryogenesis. (NIH R01 RR15402). ZFIN Direct Data Submission: 2001.
2. Cheng, Y.C.; Hsieh, F.Y.; Chiang, M.C.; Scotting, P.J.; Shih, H.Y.; Lin, S.J.; Wu, H.L.; Lee, H.T. Akt1 mediates neuronal differentiation in zebrafish via a reciprocal interaction with notch signaling. *PloS one* **2013**, *8*, e54262, doi:10.1371/journal.pone.0054262.
3. Jensen, P.J.; Gunter, L.B.; Carayannopoulos, M.O. Akt2 modulates glucose availability and downstream apoptotic pathways during development. *The Journal of biological chemistry* **2010**, *285*, 17673-17680, doi:10.1074/jbc.M109.079343.
4. Zhang, D.; Wang, J.; Zhou, C.; Xiao, W. Zebrafish akt2 is essential for survival, growth, bone development, and glucose homeostasis. *Mechanisms of development* **2017**, *143*, 42-52, doi:10.1016/j.mod.2017.01.004.
5. Roostalu, U.; Strahle, U. In vivo imaging of molecular interactions at damaged sarcolemma. *Developmental cell* **2012**, *22*, 515-529, doi:10.1016/j.devcel.2011.12.008.
6. Thisse, C.; Thisse, B. High Throughput Expression Analysis of ZF-Models Consortium Clones. ZFIN Direct Data Submission.: 2005.
7. Thisse, B.; Thisse, C. Fast Release Clones: A High Throughput Expression Analysis. ZFIN Direct Data Submission: 2004.
8. Takamiya, M.; Weger, B.D.; Schindler, S.; Beil, T.; Yang, L.; Armant, O.; Ferg, M.; Schlunck, G.; Reinhard, T.; Dickmeis, T., et al. Molecular description of eye defects in the zebrafish Pax6b mutant, sunrise, reveals a Pax6b-dependent genetic network in the developing anterior chamber. *PloS one* **2015**, *10*, e0117645, doi:10.1371/journal.pone.0117645.
9. Zhang, D.; Golubkov, V.S.; Han, W.; Correa, R.G.; Zhou, Y.; Lee, S.; Strongin, A.Y.; Dong, P.D. Identification of Annexin A4 as a hepatopancreas factor involved in liver cell survival. *Developmental biology* **2014**, *395*, 96-110, doi:10.1016/j.ydbio.2014.08.025.
10. Korzh, S.; Winata, C.L.; Zheng, W.; Yang, S.; Yin, A.; Ingham, P.; Korzh, V.; Gong, Z. The interaction of epithelial Ihha and mesenchymal Fgf10 in zebrafish esophageal and swimbladder development. *Developmental biology* **2011**, *359*, 262-276, doi:10.1016/j.ydbio.2011.08.024.
11. Erickson, T.; Nicolson, T. Identification of sensory hair-cell transcripts by thiouracil-tagging in zebrafish. *BMC genomics* **2015**, *16*, 842, doi:10.1186/s12864-015-2072-5.
12. Teoh, P.H.; Shu-Chien, A.C.; Chan, W.K. Pbx1 is essential for growth of zebrafish swim bladder. *Developmental dynamics : an official publication of the American Association of Anatomists* **2010**, *239*, 865-874, doi:10.1002/dvdy.22221.
13. Solomon, K.S.; Fritz, A. Concerted action of two dlx paralogs in sensory placode formation. *Development* **2002**, *129*, 3127-3136.
14. Granato, M.; van Eeden, F.J.; Schach, U.; Trowe, T.; Brand, M.; Furutani-Seiki, M.; Haffter, P.; Hammerschmidt, M.; Heisenberg, C.P.; Jiang, Y.J., et al. Genes controlling and mediating locomotion behavior of the zebrafish embryo and larva. *Development (Cambridge, England)* **1996**, *123*, 399-413.
15. Hirata, H.; Saint-Amant, L.; Waterbury, J.; Cui, W.; Zhou, W.; Li, Q.; Goldman, D.; Granato, M.; Kuwada, J.Y. accordion, a zebrafish behavioral mutant, has a muscle relaxation defect due to a mutation in the ATPase Ca2+ pump SERCA1. *Development* **2004**, *131*, 5457-5468, doi:10.1242/dev.01410.
16. Friedrich, T.; Lambert, A.M.; Masino, M.A.; Downes, G.B. Mutation of zebrafish dihydrolipoamide branched-chain transacylase E2 results in motor dysfunction and models maple syrup urine disease. *Disease models & mechanisms* **2012**, *5*, 248-258, doi:10.1242/dmm.008383.
17. Rauch, G.J.; Lyons, D.A.; Middendorf, I.; Friedlander, B.; Arana, N.; Reyes, T.; Talbot, W.S. Submission and Curation of Gene Expression Data. ZFIN Direct Data Submission: 2003.
18. Maves, L.; Waskiewicz, A.J.; Paul, B.; Cao, Y.; Tyler, A.; Moens, C.B.; Tapscott, S.J. Pbx homeodomain proteins direct Myod activity to promote fast-muscle differentiation. *Development* **2007**, *134*, 3371-3382, doi:10.1242/dev.003905.
19. Lin, C.Y.; Chen, J.S.; Loo, M.R.; Hsiao, C.C.; Chang, W.Y.; Tsai, H.J. MicroRNA-3906 regulates fast muscle differentiation through modulating the target gene homer-1b in zebrafish embryos. *PloS one* **2013**, *8*, e70187, doi:10.1371/journal.pone.0070187.
20. Ebert, A.M.; Hume, G.L.; Warren, K.S.; Cook, N.P.; Burns, C.G.; Mohideen, M.A.; Siegal, G.; Yelon, D.; Fishman, M.C.; Garrity, D.M. Calcium extrusion is critical for cardiac morphogenesis and rhythm in embryonic zebrafish hearts. *Proceedings of the National Academy of Sciences of the United States of America* **2005**, *102*, 17705-17710, doi:10.1073/pnas.0502683102.
21. Takeuchi, M.; Yamaguchi, S.; Sakakibara, Y.; Hayashi, T.; Matsuda, K.; Hara, Y.; Tanegashima, C.; Shimizu, T.; Kuraku, S.; Hibi, M. Gene expression profiling of granule cells and Purkinje cells in the zebrafish cerebellum. *The Journal of comparative neurology* **2017**, *525*, 1558-1585, doi:10.1002/cne.24114.
22. Cruz, S.; Shiao, J.C.; Liao, B.K.; Huang, C.J.; Hwang, P.P. Plasma membrane calcium ATPase required for semicircular canal formation and otolith growth in the zebrafish inner ear. *The Journal of experimental biology* **2009**, *212*, 639-647, doi:10.1242/jeb.022798.
23. Go, W.; Bessarab, D.; Korzh, V. atp2b1a regulates Ca(2+) export during differentiation and regeneration of mechanosensory hair cells in zebrafish. *Cell calcium* **2010**, *48*, 302-313, doi:10.1016/j.ceca.2010.09.012.

24. Go, W.; Korzh, V. Plasma membrane Ca(2+) ATPase Atp2b1a regulates bone mineralization in zebrafish. *Bone* **2013**, *54*, 48-57, doi:10.1016/j.bone.2013.01.026.
25. Liao, B.K.; Deng, A.N.; Chen, S.C.; Chou, M.Y.; Hwang, P.P. Expression and water calcium dependence of calcium transporter isoforms in zebrafish gill mitochondrion-rich cells. *BMC genomics* **2007**, *8*, 354, doi:10.1186/1471-2164-8-354.
26. Xia, Z.; Wei, J.; Li, Y.; Wang, J.; Li, W.; Wang, K.; Hong, X.; Zhao, L.; Chen, C.; Min, J., et al. Zebrafish slc30a10 deficiency revealed a novel compensatory mechanism of Atp2c1 in maintaining manganese homeostasis. *PLoS genetics* **2017**, *13*, e1006892, doi:10.1371/journal.pgen.1006892.
27. Chung, A.Y.; Kim, M.J.; Kim, D.; Bang, S.; Hwang, S.W.; Lim, C.S.; Lee, S.; Park, H.C.; Huh, T.L. Neuron-specific expression of atp6v0c2 in zebrafish CNS. *Developmental dynamics : an official publication of the American Association of Anatomists* **2010**, *239*, 2501-2508, doi:10.1002/dvdy.22383.
28. Hwang, J.; Kim, H.S.; Seok, J.W.; Kim, J.D.; Koun, S.; Park, S.Y.; Lee, J.; Kim, K.S.; Chang, K.T.; Ryoo, Z.Y., et al. Transcriptome analysis of the zebrafish mind bomb mutant. *Molecular genetics and genomics : MGG* **2009**, *281*, 77-85, doi:10.1007/s00438-008-0395-5.
29. Lee, K.C.; Goh, W.L.; Xu, M.; Kua, N.; Lunny, D.; Wong, J.S.; Coomber, D.; Vojtesek, B.; Lane, E.B.; Lane, D.P. Detection of the p53 response in zebrafish embryos using new monoclonal antibodies. *Oncogene* **2008**, *27*, 629-640, doi:10.1038/sj.onc.1210695.
30. Kratz, E.; Eimon, P.M.; Mukhyala, K.; Stern, H.; Zha, J.; Strasser, A.; Hart, R.; Ashkenazi, A. Functional characterization of the Bcl-2 gene family in the zebrafish. *Cell death and differentiation* **2006**, *13*, 1631-1640, doi:10.1038/sj.cdd.4402016.
31. Di Donato, V.; Auer, T.O.; Duroure, K.; Del Bene, F. Characterization of the calcium binding protein family in zebrafish. *PloS one* **2013**, *8*, e53299, doi:10.1371/journal.pone.0053299.
32. Low, S.E.; Woods, I.G.; Lachance, M.; Ryan, J.; Schier, A.F.; Saint-Amant, L. Touch responsiveness in zebrafish requires voltage-gated calcium channel 2.1b. *Journal of neurophysiology* **2012**, *108*, 148-159, doi:10.1152/jn.00839.2011.
33. Wen, H.; Linhoff, M.W.; Hubbard, J.M.; Nelson, N.R.; Stensland, D.; Dallman, J.; Mandel, G.; Brehm, P. Zebrafish calls for reinterpretation for the roles of P/Q calcium channels in neuromuscular transmission. *The Journal of neuroscience : the official journal of the Society for Neuroscience* **2013**, *33*, 7384-7392, doi:10.1523/jneurosci.5839-12.2013.
34. Jelen, N.; Ule, J.; Zivin, M.; Darnell, R.B. Evolution of Nova-dependent splicing regulation in the brain. *PLoS genetics* **2007**, *3*, 1838-1847, doi:10.1371/journal.pgen.0030173.
35. Ramachandran, K.V.; Hennessey, J.A.; Barnett, A.S.; Yin, X.; Stadt, H.A.; Foster, E.; Shah, R.A.; Yazawa, M.; Dolmetsch, R.E.; Kirby, M.L., et al. Calcium influx through L-type CaV1.2 Ca2+ channels regulates mandibular development. *The Journal of clinical investigation* **2013**, *123*, 1638-1646, doi:10.1172/JCI66903.
36. Muntean, B.S.; Jin, X.; Williams, F.E.; Nauli, S.M. Primary cilium regulates CaV1.2 expression through Wnt signaling. *Journal of cellular physiology* **2014**, *229*, 1926-1934, doi:10.1002/jcp.24642.
37. Jin, X.; Muntean, B.S.; Aal-Aaboda, M.S.; Duan, Q.; Zhou, J.; Nauli, S.M. L-type calcium channel modulates cystic kidney phenotype. *Biochimica et biophysica acta* **2014**, *1842*, 1518-1526, doi:10.1016/j.bbadis.2014.06.001.
38. Andersen, N.D.; Ramachandran, K.V.; Bao, M.M.; Kirby, M.L.; Pitt, G.S.; Hutson, M.R. Calcium signaling regulates ventricular hypertrophy during development independent of contraction or blood flow. *Journal of molecular and cellular cardiology* **2015**, *80*, 1-9, doi:10.1016/j.yjmcc.2014.12.016.
39. Rottbauer, W.; Baker, K.; Wo, Z.G.; Mohideen, M.A.; Cantiello, H.F.; Fishman, M.C. Growth and function of the embryonic heart depend upon the cardiac-specific L-type calcium channel alpha1 subunit. *Developmental cell* **2001**, *1*, 265-275.
40. Trapani, J.G.; Nicolson, T. Mechanism of spontaneous activity in afferent neurons of the zebrafish lateral-line organ. *The Journal of neuroscience : the official journal of the Society for Neuroscience* **2011**, *31*, 1614-1623, doi:10.1523/JNEUROSCI.3369-10.2011.
41. Sheets, L.; Kindt, K.S.; Nicolson, T. Presynaptic CaV1.3 channels regulate synaptic ribbon size and are required for synaptic maintenance in sensory hair cells. *The Journal of neuroscience : the official journal of the Society for Neuroscience* **2012**, *32*, 17273-17286, doi:10.1523/JNEUROSCI.3005-12.2012.
42. Sanhueza, D.; Montoya, A.; Sierralta, J.; Kukuljan, M. Expression of voltage-activated calcium channels in the early zebrafish embryo. *Zygote* **2009**, *17*, 131-135, doi:10.1017/S0967199408005108.
43. Sheets, L.; Trapani, J.G.; Mo, W.; Obholzer, N.; Nicolson, T. Ribeye is required for presynaptic Ca(V)1.3a channel localization and afferent innervation of sensory hair cells. *Development* **2011**, *138*, 1309-1319, doi:10.1242/dev.059451.
44. Lv, C.; Stewart, W.J.; Akanyeti, O.; Frederick, C.; Zhu, J.; Santos-Sacchi, J.; Sheets, L.; Liao, J.C.; Zenisek, D. Synaptic Ribbons Require Ribeye for Electron Density, Proper Synaptic Localization, and Recruitment of Calcium Channels. *Cell reports* **2016**, *15*, 2784-2795, doi:10.1016/j.celrep.2016.05.045.
45. Jia, S.; Muto, A.; Orisme, W.; Henson, H.E.; Parupalli, C.; Ju, B.; Baier, H.; Taylor, M.R. Zebrafish Cacna1fa is required for cone photoreceptor function and synaptic ribbon formation. *Human molecular genetics* **2014**, *23*, 2981-2994, doi:10.1093/hmg/ddu009.

46. Pujic, Z.; Omori, Y.; Tsujikawa, M.; Thisse, B.; Thisse, C.; Malicki, J. Reverse genetic analysis of neurogenesis in the zebrafish retina. *Developmental biology* **2006**, *293*, 330-347, doi:10.1016/j.ydbio.2005.12.056.
47. Chernyavskaya, Y.; Ebert, A.M.; Milligan, E.; Garrity, D.M. Voltage-gated calcium channel CACNB2 (beta2.1) protein is required in the heart for control of cell proliferation and heart tube integrity. *Developmental dynamics : an official publication of the American Association of Anatomists* **2012**, *241*, 648-662, doi:10.1002/dvdy.23746.
48. Ebert, A.M.; McAnelly, C.A.; Srinivasan, A.; Mueller, R.L.; Garrity, D.B.; Garrity, D.M. The calcium channel beta2 (CACNB2) subunit repertoire in teleosts. *BMC molecular biology* **2008**, *9*, 38, doi:10.1186/1471-2199-9-38.
49. Zhou, W.; Horstick, E.J.; Hirata, H.; Kuwada, J.Y. Identification and expression of voltage-gated calcium channel beta subunits in Zebrafish. *Developmental dynamics : an official publication of the American Association of Anatomists* **2008**, *237*, 3842-3852, doi:10.1002/dvdy.21776.
50. Roy, B.; Ahmed, K.T.; Cunningham, M.E.; Ferdous, J.; Mukherjee, R.; Zheng, W.; Chen, X.Z.; Ali, D.W. Zebrafish TARP Cacng2 is required for the expression and normal development of AMPA receptors at excitatory synapses. *Developmental neurobiology* **2016**, *76*, 487-506, doi:10.1002/dneu.22327.
51. Colombo, A.; Palma, K.; Armijo, L.; Mione, M.; Signore, I.A.; Morales, C.; Guerrero, N.; Meynard, M.M.; Perez, R.; Suazo, J., et al. Daam1a mediates asymmetric habenular morphogenesis by regulating dendritic and axonal outgrowth. *Development (Cambridge, England)* **2013**, *140*, 3997-4007, doi:10.1242/dev.091934.
52. Husken, U.; Stickney, H.L.; Gestri, G.; Bianco, I.H.; Faro, A.; Young, R.M.; Roussigne, M.; Hawkins, T.A.; Beretta, C.A.; Brinkmann, I., et al. Tcf7l2 is required for left-right asymmetric differentiation of habenular neurons. *Current biology : CB* **2014**, *24*, 2217-2227, doi:10.1016/j.cub.2014.08.006.
53. Bhoyar, R.C.; Jadhao, A.G.; Sivasubbu, S.; Singh, A.R.; Sabharwal, A.; Palande, N.V.; Biswas, S. Neuroanatomical demonstration of calbindin 2a- and calbindin 2b-like calcium binding proteins in the early embryonic development of zebrafish: mRNA study. *International journal of developmental neuroscience : the official journal of the International Society for Developmental Neuroscience* **2017**, *60*, 26-33, doi:10.1016/j.ijdevneu.2017.03.012.
54. Duggan, C.D.; DeMaria, S.; Baudhuin, A.; Stafford, D.; Ngai, J. Foxg1 is required for development of the vertebrate olfactory system. *The Journal of neuroscience : the official journal of the Society for Neuroscience* **2008**, *28*, 5229-5239, doi:10.1523/jneurosci.1134-08.2008.
55. Yokoi, H.; Yan, Y.L.; Miller, M.R.; BreMiller, R.A.; Catchen, J.M.; Johnson, E.A.; Postlethwait, J.H. Expression profiling of zebrafish sox9 mutants reveals that Sox9 is required for retinal differentiation. *Developmental biology* **2009**, *329*, 1-15, doi:10.1016/j.ydbio.2009.01.002.
56. Bae, Y.K.; Kani, S.; Shimizu, T.; Tanabe, K.; Nojima, H.; Kimura, Y.; Higashijima, S.; Hibi, M. Anatomy of zebrafish cerebellum and screen for mutations affecting its development. *Developmental biology* **2009**, *330*, 406-426, doi:10.1016/j.ydbio.2009.04.013.
57. Castro, A.; Becerra, M.; Manso, M.J.; Anadon, R. Calretinin immunoreactivity in the brain of the zebrafish, Danio rerio: distribution and comparison with some neuropeptides and neurotransmitter-synthesizing enzymes. I. Olfactory organ and forebrain. *The Journal of comparative neurology* **2006**, *494*, 435-459, doi:10.1002/cne.20782.
58. Sahu, S.U.; Visetsouk, M.R.; Garde, R.J.; Hennes, L.; Kwas, C.; Gutzman, J.H. Calcium signals drive cell shape changes during zebrafish midbrain-hindbrain boundary formation. *Molecular biology of the cell* **2017**, *28*, 875-882, doi:10.1091/mbc.E16-08-0561.
59. Hung, I.C.; Cherng, B.W.; Hsu, W.M.; Lee, S.J. Calnexin is required for zebrafish posterior lateral line development. *The International journal of developmental biology* **2013**, *57*, 427-438, doi:10.1387/ijdb.120166sl.
60. Kudoh, T.; Tsang, M.; Hukriede, N.A.; Chen, X.; Dedekian, M.; Clarke, C.J.; Kiang, A.; Schultz, S.; Epstein, J.A.; Toyama, R., et al. A gene expression screen in zebrafish embryogenesis. ZFIN Direct Data Submission: 2001.
61. Bochner, R.; Samuelov, L.; Sarig, O.; Li, Q.; Adase, C.A.; Isakov, O.; Malchin, N.; Vodo, D.; Shayevitch, R.; Peled, A., et al. Calpain 12 Function Revealed through the Study of an Atypical Case of Autosomal Recessive Congenital Ichthyosis. *The Journal of investigative dermatology* **2017**, *137*, 385-393, doi:10.1016/j.jid.2016.07.043.
62. Gan-Or, Z.; Bouslam, N.; Birouk, N.; Lissouba, A.; Chambers, D.B.; Veriepe, J.; Androschuk, A.; Laurent, S.B.; Rochefort, D.; Spiegelman, D., et al. Mutations in CAPN1 Cause Autosomal-Recessive Hereditary Spastic Paraplegia. *American journal of human genetics* **2016**, *98*, 1038-1046, doi:10.1016/j.ajhg.2016.04.002.
63. Lepage, S.E.; Bruce, A.E. Characterization and comparative expression of zebrafish calpain system genes during early development. *Developmental dynamics : an official publication of the American Association of Anatomists* **2008**, *237*, 819-829, doi:10.1002/dvdy.21459.
64. Tao, T.; Shi, H.; Guan, Y.; Huang, D.; Chen, Y.; Lane, D.P.; Chen, J.; Peng, J. Def defines a conserved nucleolar pathway that leads p53 to proteasome-independent degradation. *Cell research* **2013**, *23*, 620-634, doi:10.1038/cr.2013.16.
65. Herberger, A.L.; Loretz, C.A. Morpholino oligonucleotide knockdown of the extracellular calcium-sensing receptor impairs early skeletal development in zebrafish. *Comparative biochemistry and physiology. Part A, Molecular & integrative physiology* **2013**, *166*, 470-481, doi:10.1016/j.cbpa.2013.07.027.

66. Lin, C.H.; Su, C.H.; Hwang, P.P. Calcium-sensing receptor mediates Ca(2+) homeostasis by modulating expression of PTH and stanniocalcin. *Endocrinology* **2014**, *155*, 56-67, doi:10.1210/en.2013-1608.
67. Lin, C.H.; Hu, H.J.; Hwang, P.P. Molecular Physiology of the Hypocalcemic Action of Fibroblast Growth Factor 23 in Zebrafish (Danio rerio). *Endocrinology* **2017**, *158*, 1347-1358, doi:10.1210/en.2016-1883.
68. Jain, R.A.; Wolman, M.A.; Marsden, K.C.; Nelson, J.C.; Shoenhard, H.; Echeverry, F.A.; Szi, C.; Bell, H.; Skinner, J.; Cobbs, E.N., et al. A Forward Genetic Screen in Zebrafish Identifies the G-Protein-Coupled Receptor CaSR as a Modulator of Sensorimotor Decision Making. *Current biology : CB* **2018**, *28*, 1357-1369 e1355, doi:10.1016/j.cub.2018.03.025.
69. Kwong, R.W.; Auprix, D.; Perry, S.F. Involvement of the calcium-sensing receptor in calcium homeostasis in larval zebrafish exposed to low environmental calcium. *American journal of physiology. Regulatory, integrative and comparative physiology* **2014**, *306*, R211-221, doi:10.1152/ajpregu.00350.2013.
70. Astin, J.W.; Haggerty, M.J.; Okuda, K.S.; Le Guen, L.; Misa, J.P.; Tromp, A.; Hogan, B.M.; Crosier, K.E.; Crosier, P.S. Vegfd can compensate for loss of Vegfc in zebrafish facial lymphatic sprouting. *Development (Cambridge, England)* **2014**, *141*, 2680-2690, doi:10.1242/dev.106591.
71. Jaffe, K.M.; Grimes, D.T.; Schottenfeld-Roames, J.; Werner, M.E.; Ku, T.S.; Kim, S.K.; Pelliccia, J.L.; Morante, N.F.; Mitchell, B.J.; Burdine, R.D. c21orf59/kurly Controls Both Cilia Motility and Polarization. *Cell reports* **2016**, *14*, 1841-1849, doi:10.1016/j.celrep.2016.01.069.
72. Norden, C.; Young, S.; Link, B.A.; Harris, W.A. Actomyosin is the main driver of interkinetic nuclear migration in the retina. *Cell* **2009**, *138*, 1195-1208, doi:10.1016/j.cell.2009.06.032.
73. Delaval, B.; Covassin, L.; Lawson, N.D.; Doxsey, S. Centrin depletion causes cyst formation and other ciliopathy-related phenotypes in zebrafish. *Cell cycle* **2011**, *10*, 3964-3972, doi:10.4161/cc.10.22.18150.
74. Marra, A.N.; Wingert, R.A. Epithelial cell fate in the nephron tubule is mediated by the ETS transcription factors etv5a and etv4 during zebrafish kidney development. *Developmental biology* **2016**, *411*, 231-245, doi:10.1016/j.ydbio.2016.01.035.
75. Yu, X.; Ng, C.P.; Habacher, H.; Roy, S. Foxj1 transcription factors are master regulators of the motile ciliogenic program. *Nature genetics* **2008**, *40*, 1445-1453, doi:10.1038/ng.263.
76. Mendoza-Ferreira, N.; Coutelier, M.; Janzen, E.; Hosseinibarkooie, S.; Lohr, H.; Schneider, S.; Milbradt, J.; Karakaya, M.; Riessland, M.; Pichlo, C., et al. Biallelic CHP1 mutation causes human autosomal recessive ataxia by impairing NHE1 function. *Neurology. Genetics* **2018**, *4*, e209, doi:10.1212/nxg.0000000000000209.
77. Ponomareva, O.Y.; Holmen, I.C.; Sperry, A.J.; Eliceiri, K.W.; Halloran, M.C. Calsyntenin-1 regulates axon branching and endosomal trafficking during sensory neuron development in vivo. *The Journal of neuroscience : the official journal of the Society for Neuroscience* **2014**, *34*, 9235-9248, doi:10.1523/jneurosci.0561-14.2014.
78. Ortiz-Medina, H.; Emond, M.R.; Jontes, J.D. Zebrafish calsyntenins mediate homophilic adhesion through their amino-terminal cadherin repeats. *Neuroscience* **2015**, *286*, 87-96, doi:10.1016/j.neuroscience.2014.11.030.
79. Lee, T.J.; Lee, J.W.; Haynes, E.M.; Eliceiri, K.W.; Halloran, M.C. The Kinesin Adaptor Calsyntenin-1 Organizes Microtubule Polarity and Regulates Dynamics during Sensory Axon Arbor Development. *Frontiers in cellular neuroscience* **2017**, *11*, 107, doi:10.3389/fncel.2017.00107.
80. Dworkin, S.; Heath, J.K.; deJong-Curtain, T.A.; Hogan, B.M.; Lieschke, G.J.; Malaterre, J.; Ramsay, R.G.; Mantamadiotis, T. CREB activity modulates neural cell proliferation, midbrain-hindbrain organization and patterning in zebrafish. *Developmental biology* **2007**, *307*, 127-141, doi:10.1016/j.ydbio.2007.04.026.
81. Melville, D.B.; Montero-Balaguer, M.; Levic, D.S.; Bradley, K.; Smith, J.R.; Hatzopoulos, A.K.; Knapik, E.W. The feelgood mutation in zebrafish dysregulates COPII-dependent secretion of select extracellular matrix proteins in skeletal morphogenesis. *Disease models & mechanisms* **2011**, *4*, 763-776, doi:10.1242/dmm.007625.
82. Thisse, C.; Thisse, B. Expression from: Unexpected Novel Relational Links Uncovered by Extensive Developmental Profiling of Nuclear Receptor Expression. . ZFIN Direct Data Submission: 2008.
83. Batut, J.; Duboe, C.; Vandel, L. Expression patterns of CREB binding protein (CREBBP) and its methylated species during zebrafish development. *The International journal of developmental biology* **2015**, *59*, 229-234, doi:10.1387/ijdb.140197LV.
84. Manchenkov, T.; Pasillas, M.P.; Haddad, G.G.; Imam, F.B. Novel Genes Critical for Hypoxic Preconditioning in Zebrafish Are Regulators of Insulin and Glucose Metabolism. *G3 (Bethesda, Md.)* **2015**, *5*, 1107-1116, doi:10.1534/g3.115.018010.
85. Feitosa, N.M.; Zhang, J.; Carney, T.J.; Metzger, M.; Korzh, V.; Bloch, W.; Hammerschmidt, M. Hemicentin 2 and Fibulin 1 are required for epidermal-dermal junction formation and fin mesenchymal cell migration during zebrafish development. *Developmental biology* **2012**, *369*, 235-248, doi:10.1016/j.ydbio.2012.06.023.
86. Baxendale, S.; Chen, C.K.; Tang, H.; Davison, C.; Hateren, L.V.; Croning, M.D.; Humphray, S.J.; Hubbard, S.J.; Ingham, P.W. Expression screening and annotation of a zebrafish myoblast cDNA library. *Gene expression patterns : GEP* **2009**, *9*, 73-82, doi:10.1016/j.gep.2008.10.003.

87. Schmidt, M.; De Maziere, A.; Smyczek, T.; Gray, A.; Parker, L.; Filvaroff, E.; French, D.; van Dijk, S.; Klumperman, J.; Ye, W. The role of Egfl7 in vascular morphogenesis. *Novartis Foundation symposium* **2007**, *283*, 18-28; discussion 28-36, 238-241.
88. De Maziere, A.; Parker, L.; Van Dijk, S.; Ye, W.; Klumperman, J. Egfl7 knockdown causes defects in the extension and junctional arrangements of endothelial cells during zebrafish vasculogenesis. *Developmental dynamics : an official publication of the American Association of Anatomists* **2008**, *237*, 580-591, doi:10.1002/dvdy.21441.
89. Rossi, A.; Kontarakis, Z.; Gerri, C.; Nolte, H.; Holper, S.; Kruger, M.; Stainier, D.Y. Genetic compensation induced by deleterious mutations but not gene knockdowns. *Nature* **2015**, *524*, 230-233, doi:10.1038/nature14580.
90. He, X.; Yan, Y.L.; DeLaurier, A.; Postlethwait, J.H. Observation of miRNA gene expression in zebrafish embryos by in situ hybridization to microRNA primary transcripts. *Zebrafish* **2011**, *8*, 1-8, doi:10.1089/zeb.2010.0680.
91. Zou, J.; Li, W.Q.; Li, Q.; Li, X.Q.; Zhang, J.T.; Liu, G.Q.; Chen, J.; Qiu, X.X.; Tian, F.J.; Wang, Z.Z., et al. Two functional microRNA-126s repress a novel target gene p21-activated kinase 1 to regulate vascular integrity in zebrafish. *Circulation research* **2011**, *108*, 201-209, doi:10.1161/circresaha.110.225045.
92. Lu, Q.; Insinna, C.; Ott, C.; Stauffer, J.; Pintado, P.A.; Rahajeng, J.; Baxa, U.; Walia, V.; Cuenca, A.; Hwang, Y.S., et al. Early steps in primary cilium assembly require EHD1/EHD3-dependent ciliary vesicle formation. *Nature cell biology* **2015**, *17*, 228-240, doi:10.1038/ncb3109.
93. Seiler, C.; Gebhart, N.; Zhang, Y.; Shinton, S.A.; Li, Y.S.; Ross, N.L.; Liu, X.; Li, Q.; Bilbee, A.N.; Varshney, G.K., et al. Mutagenesis Screen Identifies agtpbp1 and eps15L1 as Essential for T lymphocyte Development in Zebrafish. *PloS one* **2015**, *10*, e0131908, doi:10.1371/journal.pone.0131908.
94. Russell, M.W.; Raeker, M.O.; Geisler, S.B.; Thomas, P.E.; Simmons, T.A.; Bernat, J.A.; Thorsson, T.; Innis, J.W. Functional analysis of candidate genes in 2q13 deletion syndrome implicates FBLN7 and TMEM87B deficiency in congenital heart defects and FBLN7 in craniofacial malformations. *Human molecular genetics* **2014**, *23*, 4272-4284, doi:10.1093/hmg/ddu144.
95. Giustiniani, J.; Chambraud, B.; Sardin, E.; Dounane, O.; Guillemeau, K.; Nakatani, H.; Paquet, D.; Kamah, A.; Landrieu, I.; Lippens, G., et al. Immunophilin FKBP52 induces Tau-P301L filamentous assembly in vitro and modulates its activity in a model of tauopathy. *Proceedings of the National Academy of Sciences of the United States of America* **2014**, *111*, 4584-4589, doi:10.1073/pnas.1402645111.
96. Benato, F.; Colletti, E.; Skobo, T.; Moro, E.; Colombo, L.; Argenton, F.; Dalla Valle, L. A living biosensor model to dynamically trace glucocorticoid transcriptional activity during development and adult life in zebrafish. *Molecular and cellular endocrinology* **2014**, *392*, 60-72, doi:10.1016/j.mce.2014.04.015.
97. Vettori, A.; Greenald, D.; Wilson, G.K.; Peron, M.; Facchinello, N.; Markham, E.; Sinnakaruppan, M.; Matthews, L.C.; McKeating, J.A.; Argenton, F., et al. Glucocorticoids promote Von Hippel Lindau degradation and Hif-1alpha stabilization. *Proceedings of the National Academy of Sciences of the United States of America* **2017**, *114*, 9948-9953, doi:10.1073/pnas.1705338114.
98. Westcot, S.E.; Hatzold, J.; Urban, M.D.; Richetti, S.K.; Skuster, K.J.; Harm, R.M.; Lopez Cervera, R.; Umemoto, N.; McNulty, M.S.; Clark, K.J., et al. Protein-Trap Insertional Mutagenesis Uncovers New Genes Involved in Zebrafish Skin Development, Including a Neuregulin 2a-Based ErbB Signaling Pathway Required during Median Fin Fold Morphogenesis. *PloS one* **2015**, *10*, e0130688, doi:10.1371/journal.pone.0130688.
99. Yakkundi, A.; Bennett, R.; Hernandez-Negrete, I.; Delalande, J.M.; Hanna, M.; Lyubomska, O.; Arthur, K.; Short, A.; McKeen, H.; Nelson, L., et al. FKBPL is a critical antiangiogenic regulator of developmental and pathological angiogenesis. *Arteriosclerosis, thrombosis, and vascular biology* **2015**, *35*, 845-854, doi:10.1161/ATVBAHA.114.304539.
100. Lin, W.H.; Wu, C.H.; Chen, Y.C.; Chow, W.Y. Embryonic expression of zebrafish AMPA receptor genes: zygotic gria2alpha expression initiates at the midblastula transition. *Brain research* **2006**, *1110*, 46-54, doi:10.1016/j.brainres.2006.06.054.
101. Hoppmann, V.; Wu, J.J.; Soviknes, A.M.; Helvik, J.V.; Becker, T.S. Expression of the eight AMPA receptor subunit genes in the developing central nervous system and sensory organs of zebrafish. *Developmental dynamics : an official publication of the American Association of Anatomists* **2008**, *237*, 788-799, doi:10.1002/dvdy.21447.
102. Li, I.C.; Chen, Y.C.; Wang, Y.Y.; Tzeng, B.W.; Ou, C.W.; Lau, Y.Y.; Wu, K.M.; Chan, T.M.; Lin, W.H.; Hwang, S.P., et al. Zebrafish Adar2 Edits the Q/R site of AMPA receptor Subunit gria2alpha transcript to ensure normal development of nervous system and cranial neural crest cells. *PloS one* **2014**, *9*, e97133, doi:10.1371/journal.pone.0097133.
103. Fuller, C.L.; Villanueva, R.; Byrd, C.A. Changes in glutamate receptor subunit 4 expression in the deafferented olfactory bulb of zebrafish. *Brain research* **2005**, *1044*, 251-261, doi:10.1016/j.brainres.2005.03.012.
104. Scheldeman, C.; Mills, J.D.; Siekierska, A.; Serra, I.; Copmans, D.; Iyer, A.M.; Whalley, B.J.; Maes, J.; Jansen, A.C.; Lagae, L., et al. mTOR-related neuropathology in mutant tsc2 zebrafish: Phenotypic, transcriptomic and pharmacological analysis. *Neurobiology of disease* **2017**, *108*, 225-237, doi:10.1016/j.nbd.2017.09.004.

105. Cox, J.A.; Kucenas, S.; Voigt, M.M. Molecular characterization and embryonic expression of the family of N-methyl-D-aspartate receptor subunit genes in the zebrafish. *Developmental dynamics : an official publication of the American Association of Anatomists* **2005**, *234*, 756-766, doi:10.1002/dvdy.20532.
106. Tzeng, D.W.; Lin, M.H.; Chen, B.Y.; Chen, Y.C.; Chang, Y.C.; Chow, W.Y. Molecular and functional studies of tilapia (Oreochromis mossambicus) NMDA receptor NR1 subunits. *Comparative biochemistry and physiology. Part B, Biochemistry & molecular biology* **2007**, *146*, 402-411, doi:10.1016/j.cbpb.2006.11.026.
107. Xie, Y.; Kaufmann, D.; Moulton, M.J.; Panahi, S.; Gaynes, J.A.; Watters, H.N.; Zhou, D.; Xue, H.H.; Fung, C.M.; Levine, E.M., et al. Lef1-dependent hypothalamic neurogenesis inhibits anxiety. *PLoS biology* **2017**, *15*, e2002257, doi:10.1371/journal.pbio.2002257.
108. Rojas-Rivera, D.; Armisen, R.; Colombo, A.; Martinez, G.; Eguiguren, A.L.; Diaz, A.; Kiviluoto, S.; Rodriguez, D.; Patron, M.; Rizzuto, R., et al. TMBIM3/GRINA is a novel unfolded protein response (UPR) target gene that controls apoptosis through the modulation of ER calcium homeostasis. *Cell death and differentiation* **2012**, *19*, 1013-1026, doi:10.1038/cdd.2011.189.
109. Cheng, W.; Guo, L.; Zhang, Z.; Soo, H.M.; Wen, C.; Wu, W.; Peng, J. HNF factors form a network to regulate liver-enriched genes in zebrafish. *Developmental biology* **2006**, *294*, 482-496, doi:10.1016/j.ydbio.2006.03.018.
110. Henke, K.; Daane, J.M.; Hawkins, M.B.; Dooley, C.M.; Busch-Nentwich, E.M.; Stemple, D.L.; Harris, M.P. Genetic Screen for Postembryonic Development in the Zebrafish (Danio rerio): Dominant Mutations Affecting Adult Form. *Genetics* **2017**, *207*, 609-623, doi:10.1534/genetics.117.300187.
111. Petko, J.A.; Kabbani, N.; Frey, C.; Woll, M.; Hickey, K.; Craig, M.; Canfield, V.A.; Levenson, R. Proteomic and functional analysis of NCS-1 binding proteins reveals novel signaling pathways required for inner ear development in zebrafish. *BMC neuroscience* **2009**, *10*, 27, doi:10.1186/1471-2202-10-27.
112. Choksi, S.P.; Babu, D.; Lau, D.; Yu, X.; Roy, S. Systematic discovery of novel ciliary genes through functional genomics in the zebrafish. *Development* **2014**, *141*, 3410-3419, doi:10.1242/dev.108209.
113. Tsai, C.T.; Hsieh, C.S.; Chang, S.N.; Chuang, E.Y.; Ueng, K.C.; Tsai, C.F.; Lin, T.H.; Wu, C.K.; Lee, J.K.; Lin, L.Y., et al. Genome-wide screening identifies a KCNIP1 copy number variant as a genetic predictor for atrial fibrillation. *Nature communications* **2016**, *7*, 10190, doi:10.1038/ncomms10190.
114. Stetsyuk, V.; Peers, B.; Mavropoulos, A.; Verbruggen, V.; Thisse, B.; Thisse, C.; Motte, P.; Duvillie, B.; Scharfmann, R. Calsenilin is required for endocrine pancreas development in zebrafish. *Developmental dynamics : an official publication of the American Association of Anatomists* **2007**, *236*, 1517-1525, doi:10.1002/dvdy.21149.
115. Rohmann, K.N.; Tripp, J.A.; Genova, R.M.; Bass, A.H. Manipulation of BK channel expression is sufficient to alter auditory hair cell thresholds in larval zebrafish. *The Journal of experimental biology* **2014**, *217*, 2531-2539, doi:10.1242/jeb.103093.
116. Cabo, R.; Zichichi, R.; Vina, E.; Guerrera, M.C.; Vazquez, G.; Garcia-Suarez, O.; Vega, J.A.; Germana, A. Calcium-activated potassium channel SK1 is widely expressed in the peripheral nervous system and sensory organs of adult zebrafish. *Neuroscience letters* **2013**, *555*, 62-67, doi:10.1016/j.neulet.2013.09.026.
117. Chen, C.H.; Sun, Y.H.; Pei, D.S.; Zhu, Z.Y. Comparative expression of zebrafish lats1 and lats2 and their implication in gastrulation movements. *Developmental dynamics : an official publication of the American Association of Anatomists* **2009**, *238*, 2850-2859, doi:10.1002/dvdy.22105.
118. Zhou, Y.; Cashman, T.J.; Nevis, K.R.; Obregon, P.; Carney, S.A.; Liu, Y.; Gu, A.; Mosimann, C.; Sondalle, S.; Peterson, R.E., et al. Latent TGF-beta binding protein 3 identifies a second heart field in zebrafish. *Nature* **2011**, *474*, 645-648, doi:10.1038/nature10094.
119. Gays, D.; Hess, C.; Camporeale, A.; Ala, U.; Provero, P.; Mosimann, C.; Santoro, M.M. An exclusive cellular and molecular network governs intestinal smooth muscle cell differentiation in vertebrates. *Development (Cambridge, England)* **2017**, *144*, 464-478, doi:10.1242/dev.133926.
120. Nevis, K.; Obregon, P.; Walsh, C.; Guner-Ataman, B.; Burns, C.G.; Burns, C.E. Tbx1 is required for second heart field proliferation in zebrafish. *Developmental dynamics : an official publication of the American Association of Anatomists* **2013**, *242*, 550-559, doi:10.1002/dvdy.23928.
121. Kao, R.M.; Rurik, J.G.; Farr, G.H., 3rd; Dong, X.R.; Majesky, M.W.; Maves, L. Pbx4 is Required for the Temporal Onset of Zebrafish Myocardial Differentiation. *Journal of developmental biology* **2015**, *3*, 93-111, doi:10.3390/jdb3040093.
122. Burrows, J.T.; Pearson, B.J.; Scott, I.C. An in vivo requirement for the mediator subunit med14 in the maintenance of stem cell populations. *Stem cell reports* **2015**, *4*, 670-684, doi:10.1016/j.stemcr.2015.02.006.
123. Jahangiri, L.; Sharpe, M.; Novikov, N.; Gonzalez-Rosa, J.M.; Borikova, A.; Nevis, K.; Paffett-Lugassy, N.; Zhao, L.; Adams, M.; Guner-Ataman, B., et al. The AP-1 transcription factor component Fosl2 potentiates the rate of myocardial differentiation from the zebrafish second heart field. *Development (Cambridge, England)* **2016**, *143*, 113-122, doi:10.1242/dev.126136.

124. Johnson, H.J.; Gandhi, M.J.; Shafizadeh, E.; Langer, N.B.; Pierce, E.L.; Paw, B.H.; Gilligan, D.M.; Drachman, J.G. In vivo inactivation of MASTL kinase results in thrombocytopenia. *Experimental hematology* **2009**, *37*, 901-908, doi:10.1016/j.exphem.2009.05.005.
125. Prudent, J.; Popgeorgiev, N.; Bonneau, B.; Thibaut, J.; Gadet, R.; Lopez, J.; Gonzalo, P.; Rimokh, R.; Manon, S.; Houart, C., et al. Bcl-wav and the mitochondrial calcium uniporter drive gastrula morphogenesis in zebrafish. *Nature communications* **2013**, *4*, 2330, doi:10.1038/ncomms3330.
126. Soman, S.; Keatinge, M.; Moein, M.; Da Costa, M.; Mortiboys, H.; Skupin, A.; Sugunan, S.; Bazala, M.; Kuznicki, J.; Bandmann, O. Inhibition of the mitochondrial calcium uniporter rescues dopaminergic neurons in pink1(-/-) zebrafish. *The European journal of neuroscience* **2017**, *45*, 528-535, doi:10.1111/ejn.13473.
127. Shimizu, H.; Schredelseker, J.; Huang, J.; Lu, K.; Naghdi, S.; Lu, F.; Franklin, S.; Fiji, H.D.; Wang, K.; Zhu, H., et al. Mitochondrial Ca(2+) uptake by the voltage-dependent anion channel 2 regulates cardiac rhythmicity. *eLife* **2015**, *4*, doi:10.7554/eLife.04801.
128. Chen, Z.; Huang, W.; Dahme, T.; Rottbauer, W.; Ackerman, M.J.; Xu, X. Depletion of zebrafish essential and regulatory myosin light chains reduces cardiac function through distinct mechanisms. *Cardiovascular research* **2008**, *79*, 97-108, doi:10.1093/cvr/cvn073.
129. Jackson, H.E.; Ono, Y.; Wang, X.; Elworthy, S.; Cunliffe, V.T.; Ingham, P.W. The role of Sox6 in zebrafish muscle fiber type specification. *Skeletal muscle* **2015**, *5*, 2, doi:10.1186/s13395-014-0026-2.
130. Seguchi, O.; Takashima, S.; Yamazaki, S.; Asakura, M.; Asano, Y.; Shintani, Y.; Wakeno, M.; Minamino, T.; Kondo, H.; Furukawa, H., et al. A cardiac myosin light chain kinase regulates sarcomere assembly in the vertebrate heart. *The Journal of clinical investigation* **2007**, *117*, 2812-2824, doi:10.1172/JCI30804.
131. Feng, S.; Wang, S.; Wang, Y.; Yang, Q.; Wang, D.; Li, H. Identification and expression of carbonic anhydrase 2, myosin regulatory light chain 2 and selenium-binding protein 1 in zebrafish Danio rerio: Implication for age-related biomarkers. *Gene expression patterns : GEP* **2018**, *29*, 47-58, doi:10.1016/j.gep.2018.04.007.
132. Stainier, D.Y.; Fouquet, B.; Chen, J.N.; Warren, K.S.; Weinstein, B.M.; Meiler, S.E.; Mohideen, M.A.; Neuhauss, S.C.; Solnica-Krezel, L.; Schier, A.F., et al. Mutations affecting the formation and function of the cardiovascular system in the zebrafish embryo. *Development (Cambridge, England)* **1996**, *123*, 285-292.
133. Matsui, T.; Thitamadee, S.; Murata, T.; Kakinuma, H.; Nabetani, T.; Hirabayashi, Y.; Hirate, Y.; Okamoto, H.; Bessho, Y. Canopy1, a positive feedback regulator of FGF signaling, controls progenitor cell clustering during Kupffer's vesicle organogenesis. *Proceedings of the National Academy of Sciences of the United States of America* **2011**, *108*, 9881-9886, doi:10.1073/pnas.1017248108.
134. Ding, Y.; Liu, W.; Deng, Y.; Jomok, B.; Yang, J.; Huang, W.; Clark, K.J.; Zhong, T.P.; Lin, X.; Ekker, S.C., et al. Trapping cardiac recessive mutants via expression-based insertional mutagenesis screening. *Circulation research* **2013**, *112*, 606-617, doi:10.1161/circresaha.112.300603.
135. Missinato, M.A.; Tobita, K.; Romano, N.; Carroll, J.A.; Tsang, M. Extracellular component hyaluronic acid and its receptor Hmmr are required for epicardial EMT during heart regeneration. *Cardiovascular research* **2015**, *107*, 487-498, doi:10.1093/cvr/cvv190.
136. El-Rass, S.; Eisa-Beygi, S.; Khong, E.; Brand-Arzamendi, K.; Mauro, A.; Zhang, H.; Clark, K.J.; Ekker, S.C.; Wen, X.Y. Disruption of pdgfra alters endocardial and myocardial fusion during zebrafish cardiac assembly. *Biology open* **2017**, *6*, 348-357, doi:10.1242/bio.021212.
137. Chiba, A.; Watanabe-Takano, H.; Terai, K.; Fukui, H.; Miyazaki, T.; Uemura, M.; Hashimoto, H.; Hibi, M.; Fukuhara, S.; Mochizuki, N. Osteocrin, a peptide secreted from the heart and other tissues, contributes to cranial osteogenesis and chondrogenesis in zebrafish. *Development (Cambridge, England)* **2017**, *144*, 334-344, doi:10.1242/dev.143354.
138. Chang, H.W.; Wang, W.D.; Chiu, C.C.; Chen, C.H.; Wang, Y.S.; Chen, Z.Y.; Liu, W.; Tai, M.H.; Wen, Z.H.; Wu, C.Y. Ftr82 Is Critical for Vascular Patterning during Zebrafish Development. *International journal of molecular sciences* **2017**, *18*, doi:10.3390/ijms18010156.
139. Bloomekatz, J.; Singh, R.; Prall, O.W.; Dunn, A.C.; Vaughan, M.; Loo, C.S.; Harvey, R.P.; Yelon, D. Platelet-derived growth factor (PDGF) signaling directs cardiomyocyte movement toward the midline during heart tube assembly. *eLife* **2017**, *6*, doi:10.7554/eLife.21172.
140. Tan, J.; Zhao, L.; Wang, G.; Li, T.; Li, D.; Xu, Q.; Chen, X.; Shang, Z.; Wang, J.; Zhou, J. Human MLL-AF9 Overexpression Induces Aberrant Hematopoietic Expansion in Zebrafish. *BioMed research international* **2018**, *2018*, 6705842, doi:10.1155/2018/6705842.
141. Guerra, A.; Germano, R.F.; Stone, O.; Arnaout, R.; Guenther, S.; Ahuja, S.; Uribe, V.; Vanhollebeke, B.; Stainier, D.Y.; Reischauer, S. Distinct myocardial lineages break atrial symmetry during cardiogenesis in zebrafish. *eLife* **2018**, *7*, doi:10.7554/eLife.32833.
142. Gomez, G.; Lee, J.H.; Veldman, M.B.; Lu, J.; Xiao, X.; Lin, S. Identification of vascular and hematopoietic genes downstream of etsrp by deep sequencing in zebrafish. *PloS one* **2012**, *7*, e31658, doi:10.1371/journal.pone.0031658.
143. Yin, J.W.; Liang, Y.; Park, J.Y.; Chen, D.; Yao, X.; Xiao, Q.; Liu, Z.; Jiang, B.; Fu, Y.; Bao, M., et al. Mediator MED23 plays opposing roles in directing smooth muscle cell and adipocyte differentiation. *Genes & development* **2012**, *26*, 2192-2205, doi:10.1101/gad.192666.112.

144. Blasiole, B.; Kabbani, N.; Boehmler, W.; Thisse, B.; Thisse, C.; Canfield, V.; Levenson, R. Neuronal calcium sensor-1 gene ncs-1a is essential for semicircular canal formation in zebrafish inner ear. *Journal of neurobiology* **2005**, *64*, 285-297, doi:10.1002/neu.20138.
145. Kim, H.T.; Kim, E.H.; Yoo, K.W.; Lee, M.S.; Choi, J.H.; Park, H.C.; Yeo, S.Y.; Lee, D.S.; Kim, C.H. Isolation and expression analysis of Alzheimer's disease-related gene xb51 in zebrafish. *Developmental dynamics : an official publication of the American Association of Anatomists* **2008**, *237*, 3921-3926, doi:10.1002/dvdy.21806.
146. Weaver, C.J.; Leung, Y.F.; Suter, D.M. Expression dynamics of NADPH oxidases during early zebrafish development. *The Journal of comparative neurology* **2016**, *524*, 2130-2141, doi:10.1002/cne.23938.
147. Hatef, A.; Shajan, S.; Unniappan, S. Nutrient status modulates the expression of nesfatin-1 encoding nucleobindin 2A and 2B mRNAs in zebrafish gut, liver and brain. *General and comparative endocrinology* **2015**, *215*, 51-60, doi:10.1016/j.ygcen.2014.09.009.
148. Large-scale discovery of novel genetic causes of developmental disorders. *Nature* **2015**, *519*, 223-228, doi:10.1038/nature14135.
149. Piotrowski, T.; Schilling, T.F.; Brand, M.; Jiang, Y.J.; Heisenberg, C.P.; Beuchle, D.; Grandel, H.; van Eeden, F.J.; Furutani-Seiki, M.; Granato, M., et al. Jaw and branchial arch mutants in zebrafish II: anterior arches and cartilage differentiation. *Development (Cambridge, England)* **1996**, *123*, 345-356.
150. Kimmel, C.B.; Miller, C.T.; Kruze, G.; Ullmann, B.; BreMiller, R.A.; Larison, K.D.; Snyder, H.C. The shaping of pharyngeal cartilages during early development of the zebrafish. *Developmental biology* **1998**, *203*, 245-263, doi:10.1006/dbio.1998.9016.
151. Kimmel, C.B.; Ullmann, B.; Walker, M.; Miller, C.T.; Crump, J.G. Endothelin 1-mediated regulation of pharyngeal bone development in zebrafish. *Development (Cambridge, England)* **2003**, *130*, 1339-1351.
152. Walker, M.B.; Miller, C.T.; Swartz, M.E.; Eberhart, J.K.; Kimmel, C.B. phospholipase C, beta 3 is required for Endothelin1 regulation of pharyngeal arch patterning in zebrafish. *Developmental biology* **2007**, *304*, 194-207, doi:10.1016/j.ydbio.2006.12.027.
153. Amsterdam, A.; Nissen, R.M.; Sun, Z.; Swindell, E.C.; Farrington, S.; Hopkins, N. Identification of 315 genes essential for early zebrafish development. *Proceedings of the National Academy of Sciences of the United States of America* **2004**, *101*, 12792-12797, doi:10.1073/pnas.0403929101.
154. Covassin, L.D.; Siekmann, A.F.; Kacergis, M.C.; Laver, E.; Moore, J.C.; Villefranc, J.A.; Weinstein, B.M.; Lawson, N.D. A genetic screen for vascular mutants in zebrafish reveals dynamic roles for Vegf/Plcg1 signaling during artery development. *Developmental biology* **2009**, *329*, 212-226, doi:10.1016/j.ydbio.2009.02.031.
155. Burns, C.E.; Galloway, J.L.; Smith, A.C.; Keefe, M.D.; Cashman, T.J.; Paik, E.J.; Mayhall, E.A.; Amsterdam, A.H.; Zon, L.I. A genetic screen in zebrafish defines a hierarchical network of pathways required for hematopoietic stem cell emergence. *Blood* **2009**, *113*, 5776-5782, doi:10.1182/blood-2008-12-193607.
156. Zygmunt, T.; Gay, C.M.; Blondelle, J.; Singh, M.K.; Flaherty, K.M.; Means, P.C.; Herwig, L.; Krudewig, A.; Belting, H.G.; Affolter, M., et al. Semaphorin-PlexinD1 signaling limits angiogenic potential via the VEGF decoy receptor sFlt1. *Developmental cell* **2011**, *21*, 301-314, doi:10.1016/j.devcel.2011.06.033.
157. Jing, C.B.; Chen, Y.; Dong, M.; Peng, X.L.; Jia, X.E.; Gao, L.; Ma, K.; Deng, M.; Liu, T.X.; Zon, L.I., et al. Phospholipase C gamma-1 is required for granulocyte maturation in zebrafish. *Developmental biology* **2013**, *374*, 24-31, doi:10.1016/j.ydbio.2012.11.032.
158. Buhrdel, J.B.; Hirth, S.; Kessler, M.; Westphal, S.; Forster, M.; Manta, L.; Wiche, G.; Schoser, B.; Schessl, J.; Schroder, R., et al. In vivo characterization of human myofibrillar myopathy genes in zebrafish. *Biochemical and biophysical research communications* **2015**, *461*, 217-223, doi:10.1016/j.bbrc.2015.03.149.
159. Chen, J.; Zhu, R.F.; Li, F.F.; Liang, Y.L.; Wang, C.; Qin, Y.W.; Huang, S.; Zhao, X.X.; Jing, Q. MicroRNA-126a Directs Lymphangiogenesis Through Interacting With Chemokine and Flt4 Signaling in Zebrafish. *Arteriosclerosis, thrombosis, and vascular biology* **2016**, *36*, 2381-2393, doi:10.1161/atvbaha.116.308120.
160. Rottbauer, W.; Just, S.; Wessels, G.; Trano, N.; Most, P.; Katus, H.A.; Fishman, M.C. VEGF-PLCgamma1 pathway controls cardiac contractility in the embryonic heart. *Genes & development* **2005**, *19*, 1624-1634, doi:10.1101/gad.1319405.
161. Ma, A.C.; Liang, R.; Leung, A.Y. The role of phospholipase C gamma 1 in primitive hematopoiesis during zebrafish development. *Experimental hematology* **2007**, *35*, 368-373, doi:10.1016/j.exphem.2006.11.010.
162. Takada, N.; Appel, B. Identification of genes expressed by zebrafish oligodendrocytes using a differential microarray screen. *Developmental dynamics : an official publication of the American Association of Anatomists* **2010**, *239*, 2041-2047, doi:10.1002/dvdy.22338.
163. Kalen, M.; Wallgard, E.; Asker, N.; Nasevicius, A.; Athley, E.; Billgren, E.; Larson, J.D.; Wadman, S.A.; Norseng, E.; Clark, K.J., et al. Combination of reverse and chemical genetic screens reveals angiogenesis inhibitors and targets. *Chemistry & biology* **2009**, *16*, 432-441, doi:10.1016/j.chembiol.2009.02.010.
164. Bedell, V.M.; Wang, Y.; Campbell, J.M.; Poshusta, T.L.; Starker, C.G.; Krug, R.G., 2nd; Tan, W.; Penheiter, S.G.; Ma, A.C.; Leung, A.Y., et al. In vivo genome editing using a high-efficiency TALEN system. *Nature* **2012**, *491*, 114-118, doi:10.1038/nature11537.

165. Huang, H.; Ruan, H.; Aw, M.Y.; Hussain, A.; Guo, L.; Gao, C.; Qian, F.; Leung, T.; Song, H.; Kimelman, D., et al. Mypt1-mediated spatial positioning of Bmp2-producing cells is essential for liver organogenesis. *Development (Cambridge, England)* **2008**, *135*, 3209-3218, doi:10.1242/dev.024406.
166. Jayashankar, V.; Nguyen, M.J.; Carr, B.W.; Zheng, D.C.; Rosales, J.B.; Weiser, D.C. Protein phosphatase 1 beta paralogs encode the zebrafish myosin phosphatase catalytic subunit. *PloS one* **2013**, *8*, e75766, doi:10.1371/journal.pone.0075766.
167. Haug, M.F.; Gesemann, M.; Berger, M.; Neuhauss, S.C.F. Phylogeny and distribution of protein kinase C variants in the zebrafish. *The Journal of comparative neurology* **2018**, *526*, 1097-1109, doi:10.1002/cne.24395.
168. Eckfeldt, C.E.; Mendenhall, E.M.; Flynn, C.M.; Wang, T.F.; Pickart, M.A.; Grindle, S.M.; Ekker, S.C.; Verfaillie, C.M. Functional analysis of human hematopoietic stem cell gene expression using zebrafish. *PLoS biology* **2005**, *3*, e254, doi:10.1371/journal.pbio.0030254.
169. Williams, C.M.; Feng, Y.; Martin, P.; Poole, A.W. Protein kinase C alpha and beta are positive regulators of thrombus formation in vivo in a zebrafish (Danio rerio) model of thrombosis. *Journal of thrombosis and haemostasis : JTH* **2011**, *9*, 2457-2465, doi:10.1111/j.1538-7836.2011.04520.x.
170. Patten, S.A.; Sihra, R.K.; Dhami, K.S.; Coutts, C.A.; Ali, D.W. Differential expression of PKC isoforms in developing zebrafish. *International journal of developmental neuroscience : the official journal of the International Society for Developmental Neuroscience* **2007**, *25*, 155-164, doi:10.1016/j.ijdevneu.2007.02.003.
171. Nakaya, N.; Lee, H.S.; Takada, Y.; Tzchori, I.; Tomarev, S.I. Zebrafish olfactomedin 1 regulates retinal axon elongation in vivo and is a modulator of Wnt signaling pathway. *The Journal of neuroscience : the official journal of the Society for Neuroscience* **2008**, *28*, 7900-7910, doi:10.1523/jneurosci.0617-08.2008.
172. Schiffer, M.; Teng, B.; Gu, C.; Shchedrina, V.A.; Kasaikina, M.; Pham, V.A.; Hanke, N.; Rong, S.; Gueler, F.; Schroder, P., et al. Pharmacological targeting of actin-dependent dynamin oligomerization ameliorates chronic kidney disease in diverse animal models. *Nature medicine* **2015**, *21*, 601-609, doi:10.1038/nm.3843.
173. Patten, S.A.; Roy, B.; Cunningham, M.E.; Stafford, J.L.; Ali, D.W. Protein kinase Cgamma is a signaling molecule required for the developmental speeding of alpha-amino-3-hydroxyl-5-methyl-4-isoxazole-propionate receptor kinetics. *The European journal of neuroscience* **2010**, *31*, 1561-1573, doi:10.1111/j.1460-9568.2010.07216.x.
174. Patten, S.A.; Ali, D.W. PKCgamma-induced trafficking of AMPA receptors in embryonic zebrafish depends on NSF and PICK1. *Proceedings of the National Academy of Sciences of the United States of America* **2009**, *106*, 6796-6801, doi:10.1073/pnas.0811171106.
175. Malicki, J.; Neuhauss, S.C.; Schier, A.F.; Solnica-Krezel, L.; Stemple, D.L.; Stainier, D.Y.; Abdelilah, S.; Zwartkruis, F.; Rangini, Z.; Driever, W. Mutations affecting development of the zebrafish retina. *Development (Cambridge, England)* **1996**, *123*, 263-273.
176. Cui, S.; Otten, C.; Rohr, S.; Abdelilah-Seyfried, S.; Link, B.A. Analysis of aPKClambda and aPKCzeta reveals multiple and redundant functions during vertebrate retinogenesis. *Molecular and cellular neurosciences* **2007**, *34*, 431-444, doi:10.1016/j.mcn.2006.11.016.
177. Baye, L.M.; Link, B.A. Interkinetic nuclear migration and the selection of neurogenic cell divisions during vertebrate retinogenesis. *The Journal of neuroscience : the official journal of the Society for Neuroscience* **2007**, *27*, 10143-10152, doi:10.1523/jneurosci.2754-07.2007.
178. Sakaguchi, T.F.; Sadler, K.C.; Crosnier, C.; Stainier, D.Y. Endothelial signals modulate hepatocyte apicobasal polarization in zebrafish. *Current biology : CB* **2008**, *18*, 1565-1571, doi:10.1016/j.cub.2008.08.065.
179. Rohr, S.; Otten, C.; Abdelilah-Seyfried, S. Asymmetric involution of the myocardial field drives heart tube formation in zebrafish. *Circulation research* **2008**, *102*, e12-19, doi:10.1161/circresaha.107.165241.
180. Ohata, S.; Aoki, R.; Kinoshita, S.; Yamaguchi, M.; Tsuruoka-Kinoshita, S.; Tanaka, H.; Wada, H.; Watabe, S.; Tsuboi, T.; Masai, I., et al. Dual roles of Notch in regulation of apically restricted mitosis and apicobasal polarity of neuroepithelial cells. *Neuron* **2011**, *69*, 215-230, doi:10.1016/j.neuron.2010.12.026.
181. Wiweger, M.I.; Zhao, Z.; van Merkesteyn, R.J.; Roehl, H.H.; Hogendoorn, P.C. HSPG-deficient zebrafish uncovers dental aspect of multiple osteochondromas. *PloS one* **2012**, *7*, e29734, doi:10.1371/journal.pone.0029734.
182. Hudish, L.I.; Blasky, A.J.; Appel, B. miR-219 regulates neural precursor differentiation by direct inhibition of apical par polarity proteins. *Developmental cell* **2013**, *27*, 387-398, doi:10.1016/j.devcel.2013.10.015.
183. Krock, B.L.; Perkins, B.D. The Par-PrkC polarity complex is required for cilia growth in zebrafish photoreceptors. *PloS one* **2014**, *9*, e104661, doi:10.1371/journal.pone.0104661.
184. Gerlach, G.F.; Wingert, R.A. Zebrafish pronephros tubulogenesis and epithelial identity maintenance are reliant on the polarity proteins Prkc iota and zeta. *Developmental biology* **2014**, *396*, 183-200, doi:10.1016/j.ydbio.2014.08.038.
185. Raman, R.; Damle, I.; Rote, R.; Banerjee, S.; Dingare, C.; Sonawane, M. aPKC regulates apical localization of Lgl to restrict elongation of microridges in developing zebrafish epidermis. *Nature communications* **2016**, *7*, 11643, doi:10.1038/ncomms11643.

186. Hava, D.; Forster, U.; Matsuda, M.; Cui, S.; Link, B.A.; Eichhorst, J.; Wiesner, B.; Chitnis, A.; Abdelilah-Seyfried, S. Apical membrane maturation and cellular rosette formation during morphogenesis of the zebrafish lateral line. *Journal of cell science* **2009**, *122*, 687-695, doi:10.1242/jcs.032102.
187. Bit-Avragim, N.; Rohr, S.; Rudolph, F.; Van der Ven, P.; Furst, D.; Eichhorst, J.; Wiesner, B.; Abdelilah-Seyfried, S. Nuclear localization of the zebrafish tight junction protein nagie oko. *Developmental dynamics : an official publication of the American Association of Anatomists* **2008**, *237*, 83-90, doi:10.1002/dvdy.21389.
188. Panizzi, J.R.; Jessen, J.R.; Drummond, I.A.; Solnica-Krezel, L. New functions for a vertebrate Rho guanine nucleotide exchange factor in ciliated epithelia. *Development (Cambridge, England)* **2007**, *134*, 921-931, doi:10.1242/dev.02776.
189. Park, H.C.; Shin, J.; Roberts, R.K.; Appel, B. An olig2 reporter gene marks oligodendrocyte precursors in the postembryonic spinal cord of zebrafish. *Developmental dynamics : an official publication of the American Association of Anatomists* **2007**, *236*, 3402-3407, doi:10.1002/dvdy.21365.
190. Hsu, Y.C.; Willoughby, J.J.; Christensen, A.K.; Jensen, A.M. Mosaic Eyes is a novel component of the Crumbs complex and negatively regulates photoreceptor apical size. *Development (Cambridge, England)* **2006**, *133*, 4849-4859, doi:10.1242/dev.02685.
191. Oubaha, M.; Lin, M.I.; Margaron, Y.; Filion, D.; Price, E.N.; Zon, L.I.; Cote, J.F.; Gratton, J.P. Formation of a PKCzeta/beta-catenin complex in endothelial cells promotes angiopoietin-1-induced collective directional migration and angiogenic sprouting. *Blood* **2012**, *120*, 3371-3381, doi:10.1182/blood-2012-03-419721.
192. Zigman, M.; Trinh le, A.; Fraser, S.E.; Moens, C.B. Zebrafish neural tube morphogenesis requires Scribble-dependent oriented cell divisions. *Current biology : CB* **2011**, *21*, 79-86, doi:10.1016/j.cub.2010.12.005.
193. Recher, G.; Jouralet, J.; Brombin, A.; Heuze, A.; Mugniery, E.; Hermel, J.M.; Desnoulez, S.; Savy, T.; Herbomel, P.; Bourrat, F., et al. Zebrafish midbrain slow-amplifying progenitors exhibit high levels of transcripts for nucleotide and ribosome biogenesis. *Development (Cambridge, England)* **2013**, *140*, 4860-4869, doi:10.1242/dev.099010.
194. Jung, J.J.; Inamdar, S.M.; Tiwari, A.; Ye, D.; Lin, F.; Choudhury, A. Syntaxin 16 regulates lumen formation during epithelial morphogenesis. *PloS one* **2013**, *8*, e61857, doi:10.1371/journal.pone.0061857.
195. Fitzpatrick, P.; Shattil, S.J.; Ablooglu, A.J. C-terminal COOH of integrin beta1 is necessary for beta1 association with the kindlin-2 adapter protein. *The Journal of biological chemistry* **2014**, *289*, 11183-11193, doi:10.1074/jbc.M113.535369.
196. Nery, L.R.; Silva, N.E.; Fonseca, R.; Vianna, M.R.M. Presenilin-1 Targeted Morpholino Induces Cognitive Deficits, Increased Brain Abeta1-42 and Decreased Synaptic Marker PSD-95 in Zebrafish Larvae. *Neurochemical research* **2017**, *42*, 2959-2967, doi:10.1007/s11064-017-2327-4.
197. Sundvik, M.; Chen, Y.C.; Panula, P. Presenilin1 regulates histamine neuron development and behavior in zebrafish, danio rerio. *The Journal of neuroscience : the official journal of the Society for Neuroscience* **2013**, *33*, 1589-1597, doi:10.1523/JNEUROSCI.1802-12.2013.
198. Geudens, I.; Herpers, R.; Hermans, K.; Segura, I.; Ruiz de Almodovar, C.; Bussmann, J.; De Smet, F.; Vandevelde, W.; Hogan, B.M.; Siekmann, A., et al. Role of delta-like-4/Notch in the formation and wiring of the lymphatic network in zebrafish. *Arteriosclerosis, thrombosis, and vascular biology* **2010**, *30*, 1695-1702, doi:10.1161/ATVBAHA.110.203034.
199. Nornes, S.; Newman, M.; Wells, S.; Verdile, G.; Martins, R.N.; Lardelli, M. Independent and cooperative action of Psen2 with Psen1 in zebrafish embryos. *Experimental cell research* **2009**, *315*, 2791-2801, doi:10.1016/j.yexcr.2009.06.023.
200. Lee, J.; Peterson, S.M.; Freeman, J.L. Alzheimer's disease risk genes in wild-type adult zebrafish exhibit gender-specific expression changes during aging. *Neurogenetics* **2016**, *17*, 197-199, doi:10.1007/s10048-016-0485-1.
201. Nornes, S.; Groth, C.; Camp, E.; Ey, P.; Lardelli, M. Developmental control of Presenilin1 expression, endoproteolysis, and interaction in zebrafish embryos. *Experimental cell research* **2003**, *289*, 124-132.
202. Groth, C.; Nornes, S.; McCarty, R.; Tamme, R.; Lardelli, M. Identification of a second presenilin gene in zebrafish with similarity to the human Alzheimer's disease gene presenilin2. *Development genes and evolution* **2002**, *212*, 486-490, doi:10.1007/s00427-002-0269-5.
203. Moussavi Nik, S.H.; Wilson, L.; Newman, M.; Croft, K.; Mori, T.A.; Musgrave, I.; Lardelli, M. The BACE1-PSEN-AbetaPP regulatory axis has an ancient role in response to low oxygen/oxidative stress. *Journal of Alzheimer's disease : JAD* **2012**, *28*, 515-530, doi:10.3233/jad-2011-110533.
204. Shih, Y.H.; Zhang, Y.; Ding, Y.; Ross, C.A.; Li, H.; Olson, T.M.; Xu, X. Cardiac transcriptome and dilated cardiomyopathy genes in zebrafish. *Circulation. Cardiovascular genetics* **2015**, *8*, 261-269, doi:10.1161/CIRCGENETICS.114.000702.
205. Lee, J.; Peterson, S.M.; Freeman, J.L. Alzheimer's disease risk genes in wild-type adult zebrafish exhibit gender-specific expression changes during aging. In *Neurogenetics*, United States, 2016; Vol. 17, pp. 197-199.

206. Hortopan, G.A.; Dinday, M.T.; Baraban, S.C. Spontaneous seizures and altered gene expression in GABA signaling pathways in a mind bomb mutant zebrafish. *The Journal of neuroscience : the official journal of the Society for Neuroscience* **2010**, *30*, 13718-13728, doi:10.1523/jneurosci.1887-10.2010.
207. Huang, M.S.; Wang, T.K.; Liu, Y.W.; Li, Y.T.; Chi, T.H.; Chou, C.W.; Hsieh, M. Roles of carbonic anhydrase 8 in neuronal cells and zebrafish. *Biochimica et biophysica acta* **2014**, *1840*, 2829-2842, doi:10.1016/j.bbagen.2014.04.017.
208. Kyostila, K.; Syrja, P.; Jagannathan, V.; Chandrasekar, G.; Jokinen, T.S.; Seppala, E.H.; Becker, D.; Drogemuller, M.; Dietschi, E.; Drogemuller, C., et al. A missense change in the ATG4D gene links aberrant autophagy to a neurodegenerative vacuolar storage disease. *PLoS genetics* **2015**, *11*, e1005169, doi:10.1371/journal.pgen.1005169.
209. Hsiao, C.D.; Tsai, W.Y.; Tsai, H.J. Isolation and expression of two zebrafish homologues of parvalbumin genes related to chicken CPV3 and mammalian oncomodulin. *Mechanisms of development* **2002**, *119 Suppl 1*, S161-166.
210. Yang, L.; Kemadjou, J.R.; Zinsmeister, C.; Bauer, M.; Legradi, J.; Muller, F.; Pankratz, M.; Jakel, J.; Strahle, U. Transcriptional profiling reveals barcode-like toxicogenomic responses in the zebrafish embryo. *Genome biology* **2007**, *8*, R227, doi:10.1186/gb-2007-8-10-r227.
211. Cannon, J.E.; Place, E.S.; Eve, A.M.; Bradshaw, C.R.; Sesay, A.; Morrell, N.W.; Smith, J.C. Global analysis of the haematopoietic and endothelial transcriptome during zebrafish development. *Mechanisms of development* **2013**, *130*, 122-131, doi:10.1016/j.mod.2012.10.002.
212. Jurynec, M.J.; Xia, R.; Mackrill, J.J.; Gunther, D.; Crawford, T.; Flanigan, K.M.; Abramson, J.J.; Howard, M.T.; Grunwald, D.J. Selenoprotein N is required for ryanodine receptor calcium release channel activity in human and zebrafish muscle. *Proceedings of the National Academy of Sciences of the United States of America* **2008**, *105*, 12485-12490, doi:10.1073/pnas.0806015105.
213. Little, A.G.; Seebacher, F. Thyroid hormone regulates muscle function during cold acclimation in zebrafish (Danio rerio). *The Journal of experimental biology* **2013**, *216*, 3514-3521, doi:10.1242/jeb.089136.
214. Wu, H.H.; Brennan, C.; Ashworth, R. Ryanodine receptors, a family of intracellular calcium ion channels, are expressed throughout early vertebrate development. *BMC research notes* **2011**, *4*, 541, doi:10.1186/1756-0500-4-541.
215. Naganawa, Y.; Hirata, H. Developmental transition of touch response from slow muscle-mediated coilings to fast muscle-mediated burst swimming in zebrafish. *Developmental biology* **2011**, *355*, 194-204, doi:10.1016/j.ydbio.2011.04.027.
216. Hirata, H.; Wen, H.; Kawakami, Y.; Naganawa, Y.; Ogino, K.; Yamada, K.; Saint-Amant, L.; Low, S.E.; Cui, W.W.; Zhou, W., et al. Connexin 39.9 protein is necessary for coordinated activation of slow-twitch muscle and normal behavior in zebrafish. *The Journal of biological chemistry* **2012**, *287*, 1080-1089, doi:10.1074/jbc.M111.308205.
217. Hirata, H.; Watanabe, T.; Hatakeyama, J.; Sprague, S.M.; Saint-Amant, L.; Nagashima, A.; Cui, W.W.; Zhou, W.; Kuwada, J.Y. Zebrafish relatively relaxed mutants have a ryanodine receptor defect, show slow swimming and provide a model of multi-minicore disease. *Development (Cambridge, England)* **2007**, *134*, 2771-2781, doi:10.1242/dev.004531.
218. Dowling, J.J.; Arbogast, S.; Hur, J.; Nelson, D.D.; McEvoy, A.; Waugh, T.; Marty, I.; Lunardi, J.; Brooks, S.V.; Kuwada, J.Y., et al. Oxidative stress and successful antioxidant treatment in models of RYR1-related myopathy. *Brain : a journal of neurology* **2012**, *135*, 1115-1127, doi:10.1093/brain/aws036.
219. Francescatto, L.; Rothschild, S.C.; Myers, A.L.; Tombes, R.M. The activation of membrane targeted CaMK-II in the zebrafish Kupffer's vesicle is required for left-right asymmetry. *Development* **2010**, *137*, 2753-2762, doi:10.1242/dev.049627.
220. Darbandi, S.; Franck, J.P. A comparative study of ryanodine receptor (RyR) gene expression levels in a basal ray-finned fish, bichir (Polypterus ornatipinnis) and the derived euteleost zebrafish (Danio rerio). *Comparative biochemistry and physiology. Part B, Biochemistry & molecular biology* **2009**, *154*, 443-448, doi:10.1016/j.cbpb.2009.09.003.
221. Germana, A.; Marino, F.; Guerrera, M.C.; Campo, S.; de Girolamo, P.; Montalbano, G.; Germana, G.P.; Ochoa-Erena, F.J.; Ciriaco, E.; Vega, J.A. Expression and distribution of S100 protein in the nervous system of the adult zebrafish (Danio rerio). *Microscopy research and technique* **2008**, *71*, 248-255, doi:10.1002/jemt.20544.
222. Kraemer, A.M.; Saraiva, L.R.; Korsching, S.I. Structural and functional diversification in the teleost S100 family of calcium-binding proteins. *BMC evolutionary biology* **2008**, *8*, 48, doi:10.1186/1471-2148-8-48.
223. Deciphering Developmental Disorders, S. Large-scale discovery of novel genetic causes of developmental disorders. *Nature* **2015**, *519*, 223-228, doi:10.1038/nature14135.
224. Amali, A.A.; Lin, C.J.; Chen, Y.H.; Wang, W.L.; Gong, H.Y.; Rekha, R.D.; Lu, J.K.; Chen, T.T.; Wu, J.L. Overexpression of Myostatin2 in zebrafish reduces the expression of dystrophin associated protein complex (DAPC) which leads to muscle dystrophy. *Journal of biomedical science* **2008**, *15*, 595-604, doi:10.1007/s11373-008-9250-2.
225. Vogel, B.; Meder, B.; Just, S.; Laufer, C.; Berger, I.; Weber, S.; Katus, H.A.; Rottbauer, W. In-vivo characterization of human dilated cardiomyopathy genes in zebrafish. *Biochemical and biophysical research communications* **2009**, *390*, 516-522, doi:10.1016/j.bbrc.2009.09.129.

226. Guyon, J.R.; Mosley, A.N.; Jun, S.J.; Montanaro, F.; Steffen, L.S.; Zhou, Y.; Nigro, V.; Zon, L.I.; Kunkel, L.M. Delta-sarcoglycan is required for early zebrafish muscle organization. *Experimental cell research* **2005**, *304*, 105-115, doi:10.1016/j.yexcr.2004.10.032.
227. Cheng, L.; Guo, X.F.; Yang, X.Y.; Chong, M.; Cheng, J.; Li, G.; Gui, Y.H.; Lu, D.R. Delta-sarcoglycan is necessary for early heart and muscle development in zebrafish. *Biochemical and biophysical research communications* **2006**, *344*, 1290-1299, doi:10.1016/j.bbrc.2006.03.234.
228. Guyon, J.R.; Goswami, J.; Jun, S.J.; Thorne, M.; Howell, M.; Pusack, T.; Kawahara, G.; Steffen, L.S.; Galdzicki, M.; Kunkel, L.M. Genetic isolation and characterization of a splicing mutant of zebrafish dystrophin. *Human molecular genetics* **2009**, *18*, 202-211, doi:10.1093/hmg/ddn337.
229. Reischauer, S.; Levesque, M.P.; Nusslein-Volhard, C.; Sonawane, M. Lgl2 executes its function as a tumor suppressor by regulating ErbB signaling in the zebrafish epidermis. *PLoS genetics* **2009**, *5*, e1000720, doi:10.1371/journal.pgen.1000720.
230. Yang, L.; Ho, N.Y.; Muller, F.; Strahle, U. Methyl mercury suppresses the formation of the tail primordium in developing zebrafish embryos. *Toxicological sciences : an official journal of the Society of Toxicology* **2010**, *115*, 379-390, doi:10.1093/toxsci/kfq053.
231. Mouti, M.A.; Dee, C.; Coupland, S.E.; Hurlstone, A.F. Minimal contribution of ERK1/2-MAPK signalling towards the maintenance of oncogenic GNAQQ209P-driven uveal melanomas in zebrafish. *Oncotarget* **2016**, *7*, 39654-39670, doi:10.18632/oncotarget.9207.
232. Lamason, R.L.; Mohideen, M.A.; Mest, J.R.; Wong, A.C.; Norton, H.L.; Aros, M.C.; Jurynec, M.J.; Mao, X.; Humphreville, V.R.; Humbert, J.E., et al. SLC24A5, a putative cation exchanger, affects pigmentation in zebrafish and humans. *Science* **2005**, *310*, 1782-1786, doi:10.1126/science.1116238.
233. Grillo, A.S.; SantaMaria, A.M.; Kafina, M.D.; Cioffi, A.G.; Huston, N.C.; Han, M.; Seo, Y.A.; Yien, Y.Y.; Nardone, C.; Menon, A.V., et al. Restored iron transport by a small molecule promotes absorption and hemoglobinization in animals. *Science* **2017**, *356*, 608-616, doi:10.1126/science.aah3862.
234. Cooper, T.K.; Murray, K.N.; Spagnoli, S.; Spitsbergen, J.M. Primary intestinal and vertebral chordomas in laboratory zebrafish (Danio rerio). *Veterinary pathology* **2015**, *52*, 388-392, doi:10.1177/0300985814537531.
235. Shaw, G.C.; Cope, J.J.; Li, L.; Corson, K.; Hersey, C.; Ackermann, G.E.; Gwynn, B.; Lambert, A.J.; Wingert, R.A.; Traver, D., et al. Mitoferrin is essential for erythroid iron assimilation. *Nature* **2006**, *440*, 96-100, doi:10.1038/nature04512.
236. Nilsson, R.; Schultz, I.J.; Pierce, E.L.; Soltis, K.A.; Naranuntarat, A.; Ward, D.M.; Baughman, J.M.; Paradkar, P.N.; Kingsley, P.D.; Culotta, V.C., et al. Discovery of genes essential for heme biosynthesis through large-scale gene expression analysis. *Cell metabolism* **2009**, *10*, 119-130, doi:10.1016/j.cmet.2009.06.012.
237. Shamseldin, H.E.; Smith, L.L.; Kentab, A.; Alkhalidi, H.; Summers, B.; Alsedairy, H.; Xiong, Y.; Gupta, V.A.; Alkuraya, F.S. Mutation of the mitochondrial carrier SLC25A42 causes a novel form of mitochondrial myopathy in humans. *Human genetics* **2016**, *135*, 21-30, doi:10.1007/s00439-015-1608-8.
238. Shimizu, H.; Langenbacher, A.D.; Huang, J.; Wang, K.; Otto, G.; Geisler, R.; Wang, Y.; Chen, J.N. The Calcineurin-FoxO-MuRF1 signaling pathway regulates myofibril integrity in cardiomyocytes. *eLife* **2017**, *6*, doi:10.7554/eLife.27955.
239. Takeuchi, J.K.; Lou, X.; Alexander, J.M.; Sugizaki, H.; Delgado-Olguin, P.; Holloway, A.K.; Mori, A.D.; Wylie, J.N.; Munson, C.; Zhu, Y., et al. Chromatin remodelling complex dosage modulates transcription factor function in heart development. *Nature communications* **2011**, *2*, 187, doi:10.1038/ncomms1187.
240. Langenbacher, A.D.; Dong, Y.; Shu, X.; Choi, J.; Nicoll, D.A.; Goldhaber, J.I.; Philipson, K.D.; Chen, J.N. Mutation in sodium-calcium exchanger 1 (NCX1) causes cardiac fibrillation in zebrafish. *Proceedings of the National Academy of Sciences of the United States of America* **2005**, *102*, 17699-17704, doi:10.1073/pnas.0502679102.
241. Shu, X.; Huang, J.; Dong, Y.; Choi, J.; Langenbacher, A.; Chen, J.N. Na,K-ATPase alpha2 and Ncx4a regulate zebrafish left-right patterning. *Development (Cambridge, England)* **2007**, *134*, 1921-1930, doi:10.1242/dev.02851.
242. Doganli, C.; Kjaer-Sorensen, K.; Knoeckel, C.; Beck, H.C.; Nyengaard, J.R.; Honore, B.; Nissen, P.; Ribera, A.; Oxvig, C.; Lykke-Hartmann, K. The alpha2Na+/K+-ATPase is critical for skeletal and heart muscle function in zebrafish. *Journal of cell science* **2012**, *125*, 6166-6175, doi:10.1242/jcs.115808.
243. On, C.; Marshall, C.R.; Perry, S.F.; Le, H.D.; Yurkov, V.; Omelchenko, A.; Hnatowich, M.; Hryshko, L.V.; Tibbits, G.F. Characterization of zebrafish (Danio rerio) NCX4: a novel NCX with distinct electrophysiological properties. *American journal of physiology. Cell physiology* **2009**, *296*, C173-181, doi:10.1152/ajpcell.00455.2008.
244. Abouzeid, H.; Boisset, G.; Favez, T.; Youssef, M.; Marzouk, I.; Shakankiry, N.; Bayoumi, N.; Descombes, P.; Agosti, C.; Munier, F.L., et al. Mutations in the SPARC-related modular calcium-binding protein 1 gene, SMOC1, cause waardenburg anophthalmia syndrome. *American journal of human genetics* **2011**, *88*, 92-98, doi:10.1016/j.ajhg.2010.12.002.
245. Bloch-Zupan, A.; Jamet, X.; Etard, C.; Laugel, V.; Muller, J.; Geoffroy, V.; Strauss, J.P.; Pelletier, V.; Marion, V.; Poch, O., et al. Homozygosity mapping and candidate prioritization identify mutations, missed by whole-exome sequencing, in SMOC2, causing major dental developmental defects. *American journal of human genetics* **2011**, *89*, 773-781, doi:10.1016/j.ajhg.2011.11.002.

246. Melvin, V.S.; Feng, W.; Hernandez-Lagunas, L.; Artinger, K.B.; Williams, T. A morpholino-based screen to identify novel genes involved in craniofacial morphogenesis. *Developmental dynamics : an official publication of the American Association of Anatomists* **2013**, *242*, 817-831, doi:10.1002/dvdy.23969.
247. Mommaerts, H.; Esguerra, C.V.; Hartmann, U.; Luyten, F.P.; Tylzanowski, P. Smoc2 modulates embryonic myelopoiesis during zebrafish development. *Developmental dynamics : an official publication of the American Association of Anatomists* **2014**, *243*, 1375-1390, doi:10.1002/dvdy.24164.
248. Yao, Z.; Farr, G.H., 3rd; Tapscott, S.J.; Maves, L. Pbx and Prdm1a transcription factors differentially regulate subsets of the fast skeletal muscle program in zebrafish. *Biology open* **2013**, *2*, 546-555, doi:10.1242/bio.20133921.
249. Lam, S.H.; Mathavan, S.; Tong, Y.; Li, H.; Karuturi, R.K.; Wu, Y.; Vega, V.B.; Liu, E.T.; Gong, Z. Zebrafish whole-adult-organism chemogenomics for large-scale predictive and discovery chemical biology. *PLoS genetics* **2008**, *4*, e1000121, doi:10.1371/journal.pgen.1000121.
250. Pashay Ahi, E.; Walker, B.S.; Lassiter, C.S.; Jonsson, Z.O. Investigation of the effects of estrogen on skeletal gene expression during zebrafish larval head development. *PeerJ* **2016**, *4*, e1878, doi:10.7717/peerj.1878.
251. Liu, J.X.; Xu, Q.H.; Li, S.; Yu, X.; Liu, W.; Ouyang, G.; Zhang, T.; Chen, L.L. Transcriptional factors Eaf1/2 inhibit endoderm and mesoderm formation via suppressing TGF-beta signaling. *Biochimica et biophysica acta. Gene regulatory mechanisms* **2017**, *1860*, 1103-1116, doi:10.1016/j.bbagrm.2017.09.001.
252. Montague, T.G.; Schier, A.F. Vg1-Nodal heterodimers are the endogenous inducers of mesendoderm. *eLife* **2017**, *6*, doi:10.7554/eLife.28183.
253. Liu, Z.; Ning, G.; Xu, R.; Cao, Y.; Meng, A.; Wang, Q. Fscn1 is required for the trafficking of TGF-beta family type I receptors during endoderm formation. *Nature communications* **2016**, *7*, 12603, doi:10.1038/ncomms12603.
254. Chen, H.; Babino, D.; Schoenbichler, S.A.; Arkhipova, V.; Tochterle, S.; Martin, F.; Huck, C.W.; von Lintig, J.; Meyer, D. Nmnat1-Rbp7 Is a Conserved Fusion-Protein That Combines NAD+ Catalysis of Nmnat1 with Subcellular Localization of Rbp7. *PloS one* **2015**, *10*, e0143825, doi:10.1371/journal.pone.0143825.
255. Kobitski, A.Y.; Otte, J.C.; Takamiya, M.; Schafer, B.; Mertes, J.; Stegmaier, J.; Rastegar, S.; Rindone, F.; Hartmann, V.; Stotzka, R., et al. An ensemble-averaged, cell density-based digital model of zebrafish embryo development derived from light-sheet microscopy data with single-cell resolution. *Scientific reports* **2015**, *5*, 8601, doi:10.1038/srep08601.
256. Ma, Y.; Liu, X.; Liu, Z.; Wei, S.; Shang, H.; Xue, Y.; Cao, Y.; Meng, A.; Wang, Q. The Chromatin Remodeling Protein Bptf Promotes Posterior Neuroectodermal Fate by Enhancing Smad2-Activated wnt8a Expression. *The Journal of neuroscience : the official journal of the Society for Neuroscience* **2015**, *35*, 8493-8506, doi:10.1523/JNEUROSCI.0377-15.2015.
257. Ma, P.; Swartz, M.R.; Kindt, L.M.; Kangas, A.M.; Liang, J.O. Temperature Sensitivity of Neural Tube Defects in Zoep Mutants. *Zebrafish* **2015**, *12*, 448-456, doi:10.1089/zeb.2015.1113.
258. Araya, C.; Tawk, M.; Girdler, G.C.; Costa, M.; Carmona-Fontaine, C.; Clarke, J.D. Mesoderm is required for coordinated cell movements within zebrafish neural plate in vivo. *Neural development* **2014**, *9*, 9, doi:10.1186/1749-8104-9-9.
259. Terashima, A.V.; Mudumana, S.P.; Drummond, I.A. Odd skipped related 1 is a negative feedback regulator of nodal-induced endoderm development. *Developmental dynamics : an official publication of the American Association of Anatomists* **2014**, *243*, 1571-1580, doi:10.1002/dvdy.24191.
260. Huang, C.J.; Wilson, V.; Pennings, S.; MacRae, C.A.; Mullins, J. Sequential effects of spadetail, one-eyed pinhead and no tail on midline convergence of nephric primordia during zebrafish embryogenesis. *Developmental biology* **2013**, *384*, 290-300, doi:10.1016/j.ydbio.2013.07.002.
261. Slagle, C.E.; Aoki, T.; Burdine, R.D. Nodal-dependent mesendoderm specification requires the combinatorial activities of FoxH1 and Eomesodermin. *PLoS genetics* **2011**, *7*, e1002072, doi:10.1371/journal.pgen.1002072.
262. Arboleda-Estudillo, Y.; Krieg, M.; Stuhmer, J.; Licata, N.A.; Muller, D.J.; Heisenberg, C.P. Movement directionality in collective migration of germ layer progenitors. *Current biology : CB* **2010**, *20*, 161-169, doi:10.1016/j.cub.2009.11.036.
263. Kim, J.D.; Chun, H.S.; Kim, S.H.; Kim, H.S.; Kim, Y.S.; Kim, M.J.; Shin, J.; Rhee, M.; Yeo, S.Y.; Huh, T.L. Normal forebrain development may require continual Wnt antagonism until mid-somitogenesis in zebrafish. *Biochemical and biophysical research communications* **2009**, *381*, 717-721, doi:10.1016/j.bbrc.2009.02.135.
264. Latimer, A.J.; Jessen, J.R. Hgf/c-met expression and functional analysis during zebrafish embryogenesis. *Developmental dynamics : an official publication of the American Association of Anatomists* **2008**, *237*, 3904-3915, doi:10.1002/dvdy.21794.
265. Biemar, F.; Argenton, F.; Schmidtke, R.; Epperlein, S.; Peers, B.; Driever, W. Pancreas development in zebrafish: early dispersed appearance of endocrine hormone expressing cells and their convergence to form the definitive islet. *Developmental biology* **2001**, *230*, 189-203, doi:10.1006/dbio.2000.0103.
266. Deshwar, A.R.; Chng, S.C.; Ho, L.; Reversade, B.; Scott, I.C. The Apelin receptor enhances Nodal/TGFbeta signaling to ensure proper cardiac development. *eLife* **2016**, *5*, doi:10.7554/eLife.13758.

267. Kindt, L.M.; Coughlin, A.R.; Perosino, T.R.; Ersfeld, H.N.; Hampton, M.; Liang, J.O. Identification of transcripts potentially involved in neural tube closure using RNA sequencing. *Genesis* **2018**, *56*, e23096, doi:10.1002/dvg.23096.
268. Burdine, R.D.; Grimes, D.T. Antagonistic interactions in the zebrafish midline prior to the emergence of asymmetric gene expression are important for left-right patterning. *Philosophical transactions of the Royal Society of London. Series B, Biological sciences* **2016**, *371*, doi:10.1098/rstb.2015.0402.
269. Xu, P.F.; Zhu, K.Y.; Jin, Y.; Chen, Y.; Sun, X.J.; Deng, M.; Chen, S.J.; Chen, Z.; Liu, T.X. Setdb2 restricts dorsal organizer territory and regulates left-right asymmetry through suppressing fgf8 activity. *Proceedings of the National Academy of Sciences of the United States of America* **2010**, *107*, 2521-2526, doi:10.1073/pnas.0914396107.
270. Antinucci, P.; Nikolaou, N.; Meyer, M.P.; Hindges, R. Teneurin-3 specifies morphological and functional connectivity of retinal ganglion cells in the vertebrate visual system. *Cell reports* **2013**, *5*, 582-592, doi:10.1016/j.celrep.2013.09.045.
271. Mieda, M.; Kikuchi, Y.; Hirate, Y.; Aoki, M.; Okamoto, H. Compartmentalized expression of zebrafish ten-m3 and ten-m4, homologues of the Drosophila ten(m)/odd Oz gene, in the central nervous system. *Mechanisms of development* **1999**, *87*, 223-227.
272. Hortopan, G.A.; Dinday, M.T.; Baraban, S.C. Zebrafish as a model for studying genetic aspects of epilepsy. *Disease models & mechanisms* **2010**, *3*, 144-148, doi:10.1242/dmm.002139.
273. Hor, H.; Francescatto, L.; Bartesaghi, L.; Ortega-Cubero, S.; Kousi, M.; Lorenzo-Betancor, O.; Jimenez-Jimenez, F.J.; Gironell, A.; Clarimon, J.; Drechsel, O., et al. Missense mutations in TENM4, a regulator of axon guidance and central myelination, cause essential tremor. *Human molecular genetics* **2015**, *24*, 5677-5686, doi:10.1093/hmg/ddv281.
274. Sogah, V.M.; Serluca, F.C.; Fishman, M.C.; Yelon, D.L.; Macrae, C.A.; Mably, J.D. Distinct troponin C isoform requirements in cardiac and skeletal muscle. *Developmental dynamics : an official publication of the American Association of Anatomists* **2010**, *239*, 3115-3123, doi:10.1002/dvdy.22445.
275. Ho, Y.L.; Lin, Y.H.; Tsai, W.Y.; Hsieh, F.J.; Tsai, H.J. Conditional antisense-knockdown of zebrafish cardiac troponin C as a new animal model for dilated cardiomyopathy. *Circulation journal : official journal of the Japanese Circulation Society* **2009**, *73*, 1691-1697.
276. Genge, C.E.; Davidson, W.S.; Tibbits, G.F. Adult teleost heart expresses two distinct troponin C paralogs: cardiac TnC and a novel and teleost-specific ssTnC in a chamber- and temperature-dependent manner. *Physiological genomics* **2013**, *45*, 866-875, doi:10.1152/physiolgenomics.00074.2013.
277. Junker, J.P.; Noel, E.S.; Guryev, V.; Peterson, K.A.; Shah, G.; Huisken, J.; McMahon, A.P.; Berezikov, E.; Bakkers, J.; van Oudenaarden, A. Genome-wide RNA Tomography in the zebrafish embryo. *Cell* **2014**, *159*, 662-675, doi:10.1016/j.cell.2014.09.038.
278. Orr, N.; Arnaout, R.; Gula, L.J.; Spears, D.A.; Leong-Sit, P.; Li, Q.; Tarhuni, W.; Reischauer, S.; Chauhan, V.S.; Borkovich, M., et al. A mutation in the atrial-specific myosin light chain gene (MYL4) causes familial atrial fibrillation. *Nature communications* **2016**, *7*, 11303, doi:10.1038/ncomms11303.
279. Elworthy, S.; Hargrave, M.; Knight, R.; Mebus, K.; Ingham, P.W. Expression of multiple slow myosin heavy chain genes reveals a diversity of zebrafish slow twitch muscle fibres with differing requirements for Hedgehog and Prdm1 activity. *Development (Cambridge, England)* **2008**, *135*, 2115-2126, doi:10.1242/dev.015719.
280. Holterhoff, C.K.; Saunders, R.H.; Brito, E.E.; Wagner, D.S. Sequence and expression of the zebrafish alpha-actinin gene family reveals conservation and diversification among vertebrates. *Developmental dynamics : an official publication of the American Association of Anatomists* **2009**, *238*, 2936-2947, doi:10.1002/dvdy.22123.
281. Wang, X.; Ono, Y.; Tan, S.C.; Chai, R.J.; Parkin, C.; Ingham, P.W. Prdm1a and miR-499 act sequentially to restrict Sox6 activity to the fast-twitch muscle lineage in the zebrafish embryo. *Development (Cambridge, England)* **2011**, *138*, 4399-4404, doi:10.1242/dev.070516.
282. Kelu, J.J.; Webb, S.E.; Galione, A.; Miller, A.L. TPC2-mediated Ca(2+) signaling is required for the establishment of synchronized activity in developing zebrafish primary motor neurons. *Developmental biology* **2018**, *438*, 57-68, doi:10.1016/j.ydbio.2018.02.011.
283. Kelu, J.J.; Webb, S.E.; Parrington, J.; Galione, A.; Miller, A.L. Ca(2+) release via two-pore channel type 2 (TPC2) is required for slow muscle cell myofibrillogenesis and myotomal patterning in intact zebrafish embryos. *Developmental biology* **2017**, *425*, 109-129, doi:10.1016/j.ydbio.2017.03.031.
284. Kelu, J.J.; Chan, H.L.; Webb, S.E.; Cheng, A.H.; Ruas, M.; Parrington, J.; Galione, A.; Miller, A.L. Two-Pore Channel 2 activity is required for slow muscle cell-generated Ca(2+) signaling during myogenesis in intact zebrafish. *The International journal of developmental biology* **2015**, *59*, 313-325, doi:10.1387/ijdb.150206am.
285. Prober, D.A.; Zimmerman, S.; Myers, B.R.; McDermott, B.M., Jr.; Kim, S.H.; Caron, S.; Rihel, J.; Solnica-Krezel, L.; Julius, D.; Hudspeth, A.J., et al. Zebrafish TRPA1 channels are required for chemosensation but not for thermosensation or mechanosensory hair cell function. *The Journal of neuroscience : the official journal of the Society for Neuroscience* **2008**, *28*, 10102-10110, doi:10.1523/JNEUROSCI.2740-08.2008.
286. Faucherre, A.; Nargeot, J.; Mangoni, M.E.; Jopling, C. piezo2b regulates vertebrate light touch response. *The Journal of neuroscience : the official journal of the Society for Neuroscience* **2013**, *33*, 17089-17094, doi:10.1523/JNEUROSCI.0522-13.2013.

287. Gau, P.; Curtright, A.; Condon, L.; Raible, D.W.; Dhaka, A. An ancient neurotrophin receptor code; a single Runx/Cbfbeta complex determines somatosensory neuron fate specification in zebrafish. *PLoS genetics* **2017**, *13*, e1006884, doi:10.1371/journal.pgen.1006884.
288. Yu, P.C.; Gu, S.Y.; Bu, J.W.; Du, J.L. TRPC1 is essential for in vivo angiogenesis in zebrafish. *Circulation research* **2010**, *106*, 1221-1232, doi:10.1161/CIRCRESAHA.109.207670.
289. Graham, D.M.; Huang, L.; Robinson, K.R.; Messerli, M.A. Epidermal keratinocyte polarity and motility require Ca(2)(+) influx through TRPV1. *Journal of cell science* **2013**, *126*, 4602-4613, doi:10.1242/jcs.122192.
290. Von Niederhausern, V.; Kastenhuber, E.; Stauble, A.; Gesemann, M.; Neuhauss, S.C. Phylogeny and expression of canonical transient receptor potential (TRPC) genes in developing zebrafish. *Developmental dynamics : an official publication of the American Association of Anatomists* **2013**, *242*, 1427-1441, doi:10.1002/dvdy.24041.
291. Moller, C.C.; Mangos, S.; Drummond, I.A.; Reiser, J. Expression of trpC1 and trpC6 orthologs in zebrafish. *Gene expression patterns : GEP* **2008**, *8*, 291-296, doi:10.1016/j.gep.2008.02.002.
292. Suzuki, H.; Nikaido, M.; Hagino-Yamagishi, K.; Okada, N. Distinct functions of two olfactory marker protein genes derived from teleost-specific whole genome duplication. *BMC evolutionary biology* **2015**, *15*, 245, doi:10.1186/s12862-015-0530-y.
293. Ahuja, G.; Bozorg Nia, S.; Zapilko, V.; Shiriagin, V.; Kowatschew, D.; Oka, Y.; Korsching, S.I. Kappe neurons, a novel population of olfactory sensory neurons. *Scientific reports* **2014**, *4*, 4037, doi:10.1038/srep04037.
294. Sato, Y.; Miyasaka, N.; Yoshihara, Y. Mutually exclusive glomerular innervation by two distinct types of olfactory sensory neurons revealed in transgenic zebrafish. *The Journal of neuroscience : the official journal of the Society for Neuroscience* **2005**, *25*, 4889-4897, doi:10.1523/JNEUROSCI.0679-05.2005.
295. Kastenhuber, E.; Gesemann, M.; Mickoleit, M.; Neuhauss, S.C. Phylogenetic analysis and expression of zebrafish transient receptor potential melastatin family genes. *Developmental dynamics : an official publication of the American Association of Anatomists* **2013**, *242*, 1236-1249, doi:10.1002/dvdy.24020.
296. Yoshida, Y.; Saitoh, K.; Aihara, Y.; Okada, S.; Misaka, T.; Abe, K. Transient receptor potential channel M5 and phospholipaseC-beta2 colocalizing in zebrafish taste receptor cells. *Neuroreport* **2007**, *18*, 1517-1520, doi:10.1097/WNR.0b013e3282ec6874.
297. Arjona, F.J.; Chen, Y.X.; Flik, G.; Bindels, R.J.; Hoenderop, J.G. Tissue-specific expression and in vivo regulation of zebrafish orthologues of mammalian genes related to symptomatic hypomagnesemia. *Pflugers Archiv : European journal of physiology* **2013**, *465*, 1409-1421, doi:10.1007/s00424-013-1275-3.
298. Elizondo, M.R.; Arduini, B.L.; Paulsen, J.; MacDonald, E.L.; Sabel, J.L.; Henion, P.D.; Cornell, R.A.; Parichy, D.M. Defective skeletogenesis with kidney stone formation in dwarf zebrafish mutant for trpm7. *Current biology : CB* **2005**, *15*, 667-671, doi:10.1016/j.cub.2005.02.050.
299. Elizondo, M.R.; Budi, E.H.; Parichy, D.M. trpm7 regulation of in vivo cation homeostasis and kidney function involves stanniocalcin 1 and fgf23. *Endocrinology* **2010**, *151*, 5700-5709, doi:10.1210/en.2010-0853.
300. Yee, N.S.; Zhou, W.; Liang, I.C. Transient receptor potential ion channel Trpm7 regulates exocrine pancreatic epithelial proliferation by Mg2+-sensitive Socs3a signaling in development and cancer. *Disease models & mechanisms* **2011**, *4*, 240-254, doi:10.1242/dmm.004564.
301. Low, S.E.; Amburgey, K.; Horstick, E.; Linsley, J.; Sprague, S.M.; Cui, W.W.; Zhou, W.; Hirata, H.; Saint-Amant, L.; Hume, R.I., et al. TRPM7 is required within zebrafish sensory neurons for the activation of touch-evoked escape behaviors. *The Journal of neuroscience : the official journal of the Society for Neuroscience* **2011**, *31*, 11633-11644, doi:10.1523/jneurosci.4950-10.2011.
302. Sah, R.; Mesirca, P.; Van den Boogert, M.; Rosen, J.; Mably, J.; Mangoni, M.E.; Clapham, D.E. Ion channel-kinase TRPM7 is required for maintaining cardiac automaticity. *Proceedings of the National Academy of Sciences of the United States of America* **2013**, *110*, E3037-3046, doi:10.1073/pnas.1311865110.
303. Decker, A.R.; McNeill, M.S.; Lambert, A.M.; Overton, J.D.; Chen, Y.C.; Lorca, R.A.; Johnson, N.A.; Brockerhoff, S.E.; Mohapatra, D.P.; MacArthur, H., et al. Abnormal differentiation of dopaminergic neurons in zebrafish trpm7 mutant larvae impairs development of the motor pattern. *Developmental biology* **2014**, *386*, 428-439, doi:10.1016/j.ydbio.2013.11.015.
304. Wingert, R.A.; Selleck, R.; Yu, J.; Song, H.D.; Chen, Z.; Song, A.; Zhou, Y.; Thisse, B.; Thisse, C.; McMahon, A.P., et al. The cdx genes and retinoic acid control the positioning and segmentation of the zebrafish pronephros. *PLoS genetics* **2007**, *3*, 1922-1938, doi:10.1371/journal.pgen.0030189.
305. Vasilyev, A.; Liu, Y.; Mudumana, S.; Mangos, S.; Lam, P.Y.; Majumdar, A.; Zhao, J.; Poon, K.L.; Kondrychyn, I.; Korzh, V., et al. Collective cell migration drives morphogenesis of the kidney nephron. *PLoS biology* **2009**, *7*, e9, doi:10.1371/journal.pbio.1000009.
306. Gau, P.; Poon, J.; Ufret-Vincenty, C.; Snelson, C.D.; Gordon, S.E.; Raible, D.W.; Dhaka, A. The zebrafish ortholog of TRPV1 is required for heat-induced locomotion. *The Journal of neuroscience : the official journal of the Society for Neuroscience* **2013**, *33*, 5249-5260, doi:10.1523/JNEUROSCI.5403-12.2013.

307. Heckel, E.; Boselli, F.; Roth, S.; Krudewig, A.; Belting, H.G.; Charvin, G.; Vermot, J. Oscillatory Flow Modulates Mechanosensitive klf2a Expression through trpv4 and trpp2 during Heart Valve Development. *Current biology : CB* **2015**, *25*, 1354-1361, doi:10.1016/j.cub.2015.03.038.
308. Kottgen, M.; Buchholz, B.; Garcia-Gonzalez, M.A.; Kotsis, F.; Fu, X.; Doerken, M.; Boehlke, C.; Steffl, D.; Tauber, R.; Wegierski, T., et al. TRPP2 and TRPV4 form a polymodal sensory channel complex. *The Journal of cell biology* **2008**, *182*, 437-447, doi:10.1083/jcb.200805124.
309. Amato, V.; Vina, E.; Calavia, M.G.; Guerrera, M.C.; Laura, R.; Navarro, M.; De Carlos, F.; Cobo, J.; Germana, A.; Vega, J.A. TRPV4 in the sensory organs of adult zebrafish. *Microscopy research and technique* **2012**, *75*, 89-96, doi:10.1002/jemt.21029.
310. Sanchez-Ramos, C.; Guerrera, M.C.; Bonnin-Arias, C.; Calavia, M.G.; Laura, R.; Germana, A.; Vega, J.A. Expression of TRPV4 in the zebrafish retina during development. *Microscopy research and technique* **2012**, *75*, 743-748, doi:10.1002/jemt.21120.
311. Mangos, S.; Liu, Y.; Drummond, I.A. Dynamic expression of the osmosensory channel trpv4 in multiple developing organs in zebrafish. *Gene expression patterns : GEP* **2007**, *7*, 480-484, doi:10.1016/j.modgep.2006.10.011.
312. Vanoevelen, J.; Janssens, A.; Huitema, L.F.; Hammond, C.L.; Metz, J.R.; Flik, G.; Voets, T.; Schulte-Merker, S. Trpv5/6 is vital for epithelial calcium uptake and bone formation. *FASEB journal : official publication of the Federation of American Societies for Experimental Biology* **2011**, *25*, 3197-3207, doi:10.1096/fj.11-183145.
313. Shu, Y.; Lou, Q.; Dai, Z.; Dai, X.; He, J.; Hu, W.; Yin, Z. The basal function of teleost prolactin as a key regulator on ion uptake identified with zebrafish knockout models. *Scientific reports* **2016**, *6*, 18597, doi:10.1038/srep18597.
314. Pan, T.C.; Liao, B.K.; Huang, C.J.; Lin, L.Y.; Hwang, P.P. Epithelial Ca(2+) channel expression and Ca(2+) uptake in developing zebrafish. *American journal of physiology. Regulatory, integrative and comparative physiology* **2005**, *289*, R1202-1211, doi:10.1152/ajpregu.00816.2004.
315. Gebriel, M.; Prabhudesai, S.; Uleberg, K.E.; Larssen, E.; Piston, D.; Bjornstad, A.H.; Moller, S.G. Zebrafish brain proteomics reveals central proteins involved in neurodegeneration. *Journal of neuroscience research* **2014**, *92*, 104-115, doi:10.1002/jnr.23297.
316. Yin, A.; Korzh, V.; Gong, Z. Perturbation of zebrafish swimbladder development by enhancing Wnt signaling in Wif1 morphants. *Biochimica et biophysica acta* **2012**, *1823*, 236-244, doi:10.1016/j.bbamcr.2011.09.018.
317. Szeto, D.P.; Kimelman, D. The regulation of mesodermal progenitor cell commitment to somitogenesis subdivides the zebrafish body musculature into distinct domains. *Genes & development* **2006**, *20*, 1923-1932, doi:10.1101/gad.1435306.
318. Row, R.H.; Kimelman, D. Bmp inhibition is necessary for post-gastrulation patterning and morphogenesis of the zebrafish tailbud. *Developmental biology* **2009**, *329*, 55-63, doi:10.1016/j.ydbio.2009.02.016.
319. Smith, C.J.; Morris, A.D.; Welsh, T.G.; Kucenas, S. Contact-mediated inhibition between oligodendrocyte progenitor cells and motor exit point glia establishes the spinal cord transition zone. *PLoS biology* **2014**, *12*, e1001961, doi:10.1371/journal.pbio.1001961.
320. Lush, M.E.; Piotrowski, T. ErbB expressing Schwann cells control lateral line progenitor cells via non-cell-autonomous regulation of Wnt/beta-catenin. *eLife* **2014**, *3*, e01832, doi:10.7554/eLife.01832.
321. Samarut, E.; Bekri, A.; Drapeau, P. Transcriptomic Analysis of Purified Embryonic Neural Stem Cells from Zebrafish Embryos Reveals Signaling Pathways Involved in Glycine-Dependent Neurogenesis. *Frontiers in molecular neuroscience* **2016**, *9*, 22, doi:10.3389/fnmol.2016.00022.
